# Sparse identification of nonlinear dynamics and Koopman operators with Shallow Recurrent Decoder Networks

**DOI:** 10.1073/pnas.2508144123

**Published:** 2026-04-17

**Authors:** Mars Liyao Gao, Jan P. Williams, J. Nathan Kutz

**Affiliations:** ^a^Paul G. Allen School of Computer Science & Engineering, University of Washington, Seattle, WA 98195; ^b^Department of Mechanical Engineering, University of Washington, Seattle, WA 98195; ^c^Department of Applied Mathematics, University of Washington, Seattle, WA 98195; ^d^Department of Electrical and Computer Engineering, University of Washington, Seattle, WA 98195

**Keywords:** model discovery, dynamical systems, machine learning, deep learning, reduced order models

## Abstract

Modeling real-world spatiotemporal data is exceptionally difficult due to inherent high dimensionality, measurement noise, partial observations, and often expensive data collection procedures. In this paper, we present Sparse Identification of Nonlinear Dynamics with SHallow REcurrent Decoder networks (SINDy-SHRED), a method to jointly solve the sensing and model identification problems with simple implementation, efficient computation, and robust performance. SINDy-SHRED uses Gated Recurrent Units to model the temporal sequence of sparse sensor measurements along with a shallow decoder network to reconstruct the full spatiotemporal field from the latent state space. Our algorithm introduces a SINDy-based regularization for which the latent space progressively converges to a SINDy-class functional, provided the projection remains within the set. In restricting SINDy to a linear model, a Koopman-SHRED model is generated. SINDy-SHRED i) learns a symbolic and interpretable generative model of a parsimonious and low-dimensional latent space for the complex spatiotemporal dynamics, ii) discovers governing equations even for well-known physical systems, iii) achieves an observed convex loss landscape, and iv) achieves superior accuracy, data efficiency, and training cost, all with fewer model parameters, than competing state-of-the-art methods. We conduct systematic experimental studies on Partial differential equation data such as turbulent flows, real-world sensor measurements for sea surface temperature, and direct video data. The interpretable SINDy and Koopman models of latent state dynamics enable stable and accurate long-term video predictions, outperforming all current baseline deep learning models in accuracy, training cost, and data requirements, including Convolutional LSTM, PredRNN, ResNet, and SimVP.

## Significance

We present sparse identification of nonlinear dynamics with shallow recurrent decoders (SINDy-SHRED), which jointly solves the sensing, model reduction and model identification problem with simple implementation, efficient computation, and robust performance. Shallow recurrent decoders map the temporal sequence of a limited number of sensor measurements to the full state space through a latent temporal sequence representation. In SINDy-SHRED, the latent space is constrained to an interpretable and parsimonious dynamical system which can be linear or nonlinear. This allows SINDy-SHRED to learn physics models even for well-known systems. Remarkably, the loss landscape is empirically convex globally, which allows for state-of-the-art performance while simultaneously maintaining the lowest computational cost, smallest model size and minimal training effort compared to leading methods.

Partial differential equations (PDEs) derived from first principles or qualitative behavior remain the most ubiquitous class of models used to describe physical, spatiotemporal phenomena. However, for complex dynamics it is often the case that the simplifying assumptions necessary to construct a PDE model can render it ineffectual for real data where the physics is high-dimensional, multiscale in nature, only partially known, or where first principles models currently do not exist. In such cases, machine learning (ML) methods offer an attractive alternative for learning both the physics and coordinates (fundamental variables) necessary to quantify the observed spatiotemporal phenomena. Many recent efforts utilizing ML techniques seek to relax the computational burden for PDE simulation by learning surrogate models to forward-simulate or predict spatiotemporal systems. However, this new machine learning paradigm frequently exhibits instabilities during the training process, unstable roll-outs when modeling future state predictions, and often yields minimal computational speedups (1). The *Shallow REcurrent Decoder* (SHRED) network (2) is a recently introduced architecture that utilizes data from sparse sensors to reconstruct and predict the entire spatiotemporal domain. Similar to Takens’ embedding theorem, SHRED models trade spatial information at a single time point for a trajectory of sensor measurements across time. Previous work has shown SHRED can achieve excellent performance in sensing and reduced order modeling examples ranging from weather and atmospheric forecasting (2), plasma physics (3), nuclear reactors (4), and turbulent flow reconstructions (5). Theoretically rooted in the classic PDE method of separation of variables (see detailed derivations in refs. 2 and 5), the decoding-only strategy of SHRED circumvents the computation of inverse pairs, i.e. an encoder and the corresponding decoder. It has been well known for decades that the computation of the inverse of a matrix is highly unstable and ill-posed (6–8). By decoding only, SHRED avoids this problem and learns a single embedding without the corresponding inversion.

In this paper, we introduce Sparse Identification of Nonlinear Dynamics with SHallow REcurrent Decoder networks (SINDy-SHRED). SINDy is a computational method that applies sparse regression techniques to perform nonlinear system identification. The resulting model is a set of ordinary differential equations (ODEs), well-suited for interpretable modeling. SINDy-SHRED exploits the latent space of recurrent neural networks for sparse sensor modeling, and enforces interpretability via a SINDy-class functional regularization, where the SINDy-class denotes functions formed by sparse linear combinations of candidate basis functions (e.g., polynomial or trigonometric) from the chosen SINDy library. Moreover, our theoretical analysis (*Theorems* 1 and 2) rigorously demonstrates key advantages of latent space modeling with SINDy over conventional neural networks, i.e. under mild conditions it is guaranteed to converge with a bounded error. In this way, SINDy-SHRED enables a robust and sample-efficient joint discovery of governing equation and coordinate system. With the correct governing equation, SINDy-SHRED can perform an accurate long-term prediction in a learned, low-dimensional latent space, and in turn allows for long-term forecasting in the original spatiotemporal (pixel) space. In further restricting SINDy to a linear model, a Koopman approximation (9) can be constructed to produce a Koopman-SHRED architecture.

**Fig. 1.**
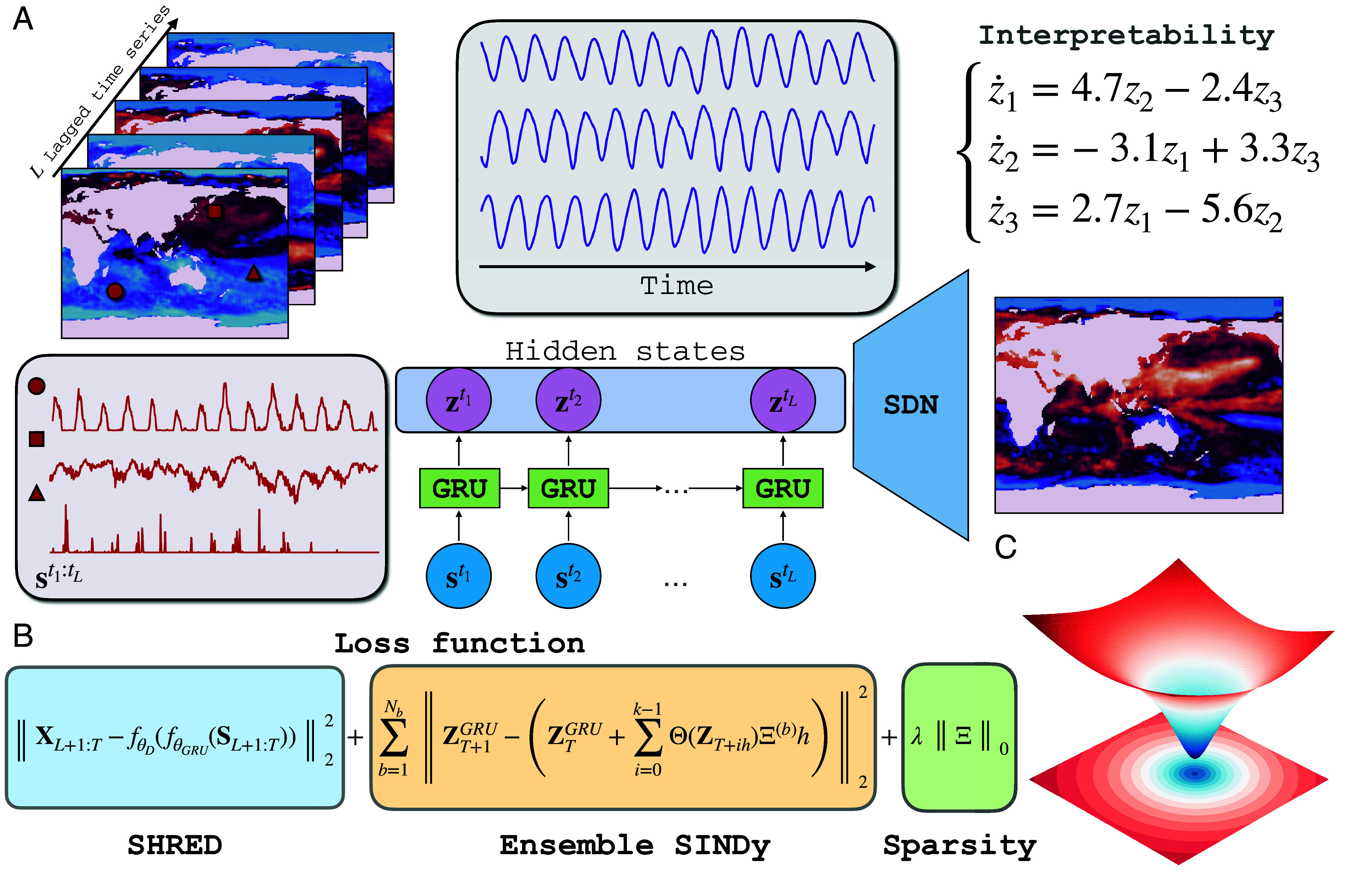
(*A*) Illustration of the SINDy-SHRED and Koopman-SHRED architecture. SINDy-SHRED transfers the original sparse sensor signal (red) to an interpretable latent representation (purple) that falls into the SINDy-class functional. This framework can be adapted into Koopman-SHRED by restricting the library Θ(·) to be linear. The shallow decoder performs a reconstruction in the state space. We obtain an interpretable linear model for the sea-surface temperature data considered (details in Section 3.3). (*B*) The loss function consists of three parts: i) the SHRED loss controls the reconstruction accuracy of the state space, ii) the Ensemble SINDy loss helps to model the parsimonious dynamics of the latent space, and iii) the sparsity constraint identifies the governing equation within this optimization framework. (*C*) We visualize the globally convex loss landscape of SINDy-SHRED as in ref. 11.

SINDy-SHRED and Koopman-SHRED are lightweight models which can perform low-rank recovery with as few as three active sensors for the high-dimensional spatiotemporal fields, which is critical for large-scale scientific data modeling and real-time control. Specifically, for *D*-dimensional fields, D+1 sensors are required for reconstructing the spatiotemporal field from sparse observations, much like localization for cellular networks (10). Moreover, the proposed architecture does not require large amounts of data during training, thereby avoiding a common pitfall in existing ML techniques for accelerating physics simulations and enabling rapid training on a single laptop. SINDy-SHRED/Koopman-SHRED are also highly reproducible with minimal effort in hyperparameter tuning due to the globally convex optimization landscape, as illustrated in the *Lower Right* panel of Fig. 1. The recommended network structure with the same set of hyperparameters, and training setting can generalize to many different datasets.^*^ In short, we demonstrate SINDy-SHRED/Koopman-SHRED to be robust and highly applicable in many modern scientific modeling problems. In what follows, we will refer generally to the proposed architecture as SINDy-SHRED, with Koopman-SHRED as a special case.

We perform a wide range of studies to demonstrate the effectiveness of SINDy-SHRED. We apply the model on the sea surface temperature data, which is a complex real-world problem. We also consider data from complex simulations of atmospheric chemistry, 2D Kolmogorov flow, and isotropic turbulence, video data of flow over a cylinder, and video data of a pendulum. The ability of SINDy-SHRED to perform well on video data is an important result for so-called “GoPro physics,” whereby physics is learned directly from video recordings. With extremely small sample size and noisy environments, SINDy-SHRED achieves governing equation identification with stable long-term predictions, thus overcoming the critical limitations for long-term forecasts of spatiotemporal phenomena that include instabilities and massive computational requirements. To summarize, the contributions of our paper are the following:

- We propose SINDy-SHRED to learn a symbolic and interpretable generative model of a parsimonious and low-dimensional latent space (fundamental variables) of recurrent neural networks for complex spatiotemporal dynamics.
- Even for the well-known physical systems considered, we discover in each case a new and hitherto unknown physics model characterizing the spatiotemporal dynamics directly from measurement or video streams. These benchmark systems include turbulent flows, videos of physical systems, and PDE simulations.
- Under weak assumptions, we establish rigorous error bounds on the robust convergence of the SINDy-SHRED algorithm which allows us to guarantee model performance, a rarity in deep learning. Moreover, we systematically observe that the algorithm has a global convex loss landscape, preventing the need for any hyperparameter tuning (except for the SINDy parameters of dimension and sparsity).
- We compare SINDy-SHRED to state-of-the-art deep learning algorithms in spatiotemporal prediction, showing that the architecture achieves superior accuracy, data efficiency, training time, and long term prediction, all with fewer model parameters.

## 1. Related Works

Traditionally, spatiotemporal physical phenomena are modeled by Partial Differential Equations (PDEs). To accelerate PDE simulations, recent efforts have leveraged neural networks to model physics. By explicitly assuming knowledge of the underlying PDE, physics-informed neural networks (12) utilize the PDE structure as a constraint for small sample learning. However, assuming the exact form of governing PDE for real data can be a strong limitation. There have been many recent works on learning and predicting PDEs directly using neural networks(13–18). Alternatively, variants of SINDy (19–21) offer a data-driven approach to identify PDEs from the spatial-temporal domain. High-dimensionality and data requirements can be prohibitive for many applications.

In parallel, efforts have been directed toward the discovery of physical laws through dimensionality reduction techniques (22–24), providing yet another perspective on the modeling of scientific data. The Mori–Zwanzig formalism (25–28) also provides a theoretical foundation for reduced-order modeling of spatiotemporal systems. The discovery of physics from a learned latent spaces has previously been explored by (29–39), yet none of these methods consider a regularization on the latent space with no explicit encoder. Yu et al. (40) proposed the idea of physics-guided learning which combines physics simulations and neural network approximations. Directly modeling physics from video is also the subject of much research in the field of robotics (41–43), computer vision (44–47) and computer graphics (48–51), as these fields all require better physics models.

From the deep learning side, combining the structure of differential equations into neural networks (52, 53) has been investigated in many tasks. As an example, when spatial-temporal modeling is framed as a video prediction problem, He et al. found (54) that random masking can be an efficient spatiotemporal learner, and deep neural networks can provide very good predictions for the next 10 to 20 frames (55–58). Generative models have also been found to be useful for scientific data modeling (12, 59–66) and data assimilation methods similarly aim to combine imperfect forecast models with sparse sensor measurements to improve running state estimates of high-dimensional systems (64–67). Unlike many of the previous works above, SINDy-SHRED i) provides a computationally efficient white-box approach for scientific data modeling; and ii) discovers the governing equations directly from data without assuming prior physics. These advantages are important for real-world scientific applications.

### 1.1. Sparse Identification of Nonlinear Dynamics (SINDy).

SINDy is a computational method that applies sparse regression techniques to perform nonlinear system identification. Given *n* snapshot data of a dynamical system $z\left(t\right)={\left[z_{1}\left(t\right),z_{2}\left(t\right),...,z_{d}\left(t\right)\right]}^{⊤}\in^{d}$, SINDy identifies the symbolic form of the following dynamical system

[1] $$\begin{array}{c} \dot{z}\left(t\right)=f\left(x\left(t\right)\right)=\Theta \left(z\left(t\right)\right)\Xi , \end{array}$$

where Θ(z) is a library of candidate nonlinear functions. The sparse coefficient matrix Ξ determines which terms in the candidate library are active, which encodes both the structure and coefficient values of the dynamical system. The candidate function library Θ(z) typically includes commonly used functions for ODE modeling such as polynomial, and trigonometric terms $\Theta \left(z\right)=[1,z,z^{2},...,\sin\left(z\right),\cos\left(z\right)]$.

Since we do not have direct access to $\frac{dz}{\mathrm{dt}}$ in real life, $\dot{z}$ is practically estimated via numerical differentiation algorithms. By solving the following sparse regression problem, the sparse coefficient Ξ identifies the governing equation from data $\dot{z}\left(t\right)=\Theta \left(z\right)\Xi +\epsilon$ with noise *ϵ*. The recovery of Ξ is theoretically guaranteed with sufficient sample sizes (68).

Despite the success of SINDy in scientific discovery in a wide range of applications (69–75), it does not scale well with high-dimensional problems. Many high-dimensional systems have a compact representation in a lower-dimensional subspace. This subspace is accessible via a coordinate transformation which contains a compact representation that follows a governing law. Take a video of a moving rod as an example, the physics of the high-dimensional pixel space is governed by a single variable *θ* which corresponds to the angular displacement. The latent variable *θ* will follow the classical ODE for pendulum moving $\ddot{\theta }=-\kappa \sin\left(\theta \right)$ where *κ* is a constant, but the values of the pixels in the original high-dimensional state space will not follow any simple forms of an ODE. To perform a joint discovery of the coordinate system and governing equations, prior works have been focusing on adapting autoencoders with SINDy either within training (22, 24) or after (29).

## 2. Methods

The shallow recurrent decoder network (SHRED) is a computational technique that utilizes recurrent neural networks to estimate high-dimensional states from limited measurements (2). The method functions by trading high-fidelity spatial information for trajectories of sparse sensor measurements at given spatial locations. Mathematically, consider a high-dimensional data series ${\left\{X_{i}\right\}}_{i=1}^{T}$ where $X_{i}\in^{N_{x}\times N_{y}}$ that represents the evolution of a spatiotemporal dynamical system, where $N_{x}$, $N_{y}$, and *T* denote number of discretized spatial grid points along two spatial dimensions, and total time steps of the system, respectively. The total time steps are $t={\left\{t_{i}\right\}}_{i=1}^{T}$. In SHRED, $N_{s}$ sensors record measurements of the evolving physical system ${\left\{X_{i}\right\}}_{i=1}^{T}$ at discrete time steps t. Denoting the time-series data of SHRED as ${\left\{s_{i}\right\}}_{i=1}^{T}$, where each $s_{i}\in R^{N_{s}}$ contains measurements of all sensors at time $t_{i}$. To incorporate the temporal information from the sensors, SHRED employs an *L*-lagged temporal sensor history, such that the input–output pair is defined as $D={\left\{\left(s_{t_{i-L}:t_{i}},X_{t_{i}}\right)\right\}}_{t_{i}=L+1}^{T}$, where $t_{i-L}:t_{i}$ is a compact notation for discrete index set $\left\{t_{i-L},t_{i-L+1},\cdots ,t_{i}\right\}$.

Provided the underlying PDE allows spatial information to propagate, these spatial effects will appear in the time history of the sensor measurements, enabling the sensing of the entire field using sparse sensor history. In vanilla SHRED, a recurrent network module is used to map the sparse sensor trajectory data into a latent space, followed by a shallow decoder to reconstruct the entire spatiotemporal domain at the current time step. SHRED generalizes the separation of variables method for solving PDEs that the GRU $f_{\theta_{\text{GRU}}}$ captures temporal evolution from sparse sensor histories, and the decoder $f_{\theta_{\text{D}}}$ reconstructs the spatial field. For a detailed discussion of this connection, please see refs. 2 and 5.

SHRED enables efficient sparse sensing that is widely applicable to many scientific problems (3–5, 76, 77). The advantage of SHRED comes from three aspects. First, SHRED only requires minimal sensor measurements. Under practical constraints, collecting full-state measurements for data prediction and control can be prohibitively expensive. Second, SHRED does not require grid-like data collection, which allows for generalization to more complex data structures. For example, it is easy to apply SHRED to graph data with an unknown underlying structure, such as human motion data on joints, robotic sensor data, and financial market data. Furthermore, SHRED is grounded in the classical technique of separation of variables (5).

### 2.1. Learning Representations for Physics Discovery Using SHRED.

To achieve a parsimonious representation of physics, it is important to find a coordinate system that effectively captures the underlying dynamics and structure of the system. In SINDy-SHRED (shown in Fig. 1), we extend the advantages of SHRED, and perform a joint discovery of coordinate systems and governing equations. This is accomplished by enforcing the latent space to follow an ODE in the SINDy class of functions.

#### 2.1.1. Finding better representations.

SHRED has a natural advantage in modeling latent governing physics due to its small model size. SHRED is based on a shallow decoder with a relatively small recurrent network structure. The relative simplicity of the model allows the latent representation to maintain many advantageous properties such as smoothness and Lipschitzness. Experimentally, we observe that the hidden state space of a SHRED model is generally very smooth. Second, SHRED does not have an explicit encoder, which avoids the potential problem of spectral bias (78). Many reduced-order modeling methods that rely on an encoder architecture struggle to learn physics and instead focus only on modeling the low-frequency information (background) (22, 24, 79). Building upon SHRED, we further incorporate SINDy to regularize the learned recurrence with a well-characterized and simple form of governing equation. This approach is inspired by the principle that, under an ideal coordinate system, physical phenomena can be described by a parsimonious dynamics model (22, 24). When the latent representation and the governing law are well aligned, this configuration is likely to capture the true underlying physics. This joint discovery results in a latent space that is both interpretable and physically meaningful, enabling robust and stable future prediction from the learned dynamics.

#### 2.1.2. Physics discovery and prediction.

By regularizing the latent dynamics to follow an ODE model, SINDy-SHRED automatically performs physics discovery straight from high-dimensional data. The resulting SINDy model captures the real physical law that governs the observed dynamics. This enables a physics discovery in the intrinsic latent coordinate system induced from the high-dimensional observation. Notably, even though the model is trained only on sensor trajectories up to a finite time horizon, the physics discovered with SINDy regularization endows it with strong extrapolative capability beyond the training window. With the discovered physics, SINDy-SHRED is able to predict future spatiotemporal evolution without requiring any further sensor inputs. After initialization with a short stream of sensor measurements, the discovered SINDy model can unroll the latent dynamics forward in time, and the decoder will map these latent trajectories back to the high-dimensional state space. Across all computational experiments, we found the discovered models robustly produce accurate long-term rollouts, which validates the discovered physics.

### 2.2. Latent Space Regularization via SINDy and Koopman.

As a compressive sensing procedure, there exist infinitely many equally valid solutions for the latent representation. Therefore, it is not necessary for the latent representation induced by SHRED to follow a well-structured differential equation. For instance, even if the exhibited dynamics are fundamentally linear, the latent representation may exhibit completely unexplainable dynamics, making the model challenging to interpret and extrapolate. Therefore, in SINDy-SHRED, our goal is to further constrain the latent representations to lie within the SINDy-class functional. This regularization promotes models that are fundamentally explainable by a SINDy-based ODE, allowing us to identify a parsimonious governing equation. The SINDy class of functions consists of a library of commonly used terms, including polynomials and Fourier series. The expressiveness of these terms allow the model to capture complex dynamics.

#### 2.2.1. SINDy as a recurrent neural network.

Incorporating SINDy within the neural network training is highly nontrivial. All prior works (22, 24, 80) require to compute temporal derivatives on the latent space $\dot{z}$ during training. However, estimating time derivatives within neural network training is inherently unstable and computationally expensive. For example in refs. 22 and 24, the derivative $\dot{z}$ is computed via the explicit solution (c.f. (22) S1.3). This operation fundamentally requires the neural network weights and the time derivative of the original state space $\dot{x}$. As a result, this estimation is highly sensitive to network fluctuations during training, causing loss spikes and training instability, while also being unstable under noise and computationally expensive when evaluating $\dot{x}$ for large-scale scientific datasets.

To resolve the problems above, we reformulate SINDy in its variational form (81) without computing the time derivatives. Inspired by ResNet (52) and Neural ODE (53), we utilize skip connections to model residual and temporal derivatives. It allows us to rewrite SINDy into a Recurrent Neural Network (RNN) which has the following form $z_{t\;+1}=z_{t}+f\left(z_{t}\right)$ where f(·) is some function of the input, and $z_{t}$ denotes z(t). From the Euler method, the ODE forward simulation via SINDy effectively falls into the category of Recurrent Neural Networks (RNNs) which has the form

[2] $$\begin{array}{c} z_{t\;+\;1}=z_{t}\;+\;f_{\Theta }\left(z_{t},\Xi ,\Delta t\right), \end{array}$$

where $f_{\Theta }\left(z_{t},\Xi ,\Delta t\right)=\Theta \left(z_{t}\right)\Xi \Delta t$ is a nonlinear function. Notice that this $f_{\Theta }\left(\cdot \right)$ follows the SINDy formulation of dynamical systems (82) rewritten in a variational form (81). We demonstrate a visual form of this design in *SI Appendix*. The application of function libraries with sparsity constraints is a manner of automatic neural architecture search (NAS) (83). Compared to all prior works (22, 29, 32), the usage of the variational form of SINDy is similar to a RNN training, which fits better as a neural network model with gradient-based training. Additionally, applying the variational form in the latent space, we avoid the need to estimate vector fields in the original high-dimensional state space, which are both computationally expensive and numerically unstable, and were required in prior works (22, 24). We utilize trajectory data $Z_{T}={\left\{z_{i}\right\}}_{i=1}^{T-1}$ and forward simulate the SINDy-based ODE using a trainable parameter Ξ. To achieve better stability and accuracy for forward integration, we use Euler integration with *k* mini-steps (with time step $\frac{\Delta t}{k}$) to obtain $Z_{T+1}$. In summary, defining $h=\frac{\Delta t}{k}$, we optimize Ξ with the following:

$$\begin{array}{c} \text{Ξ}=\;\arg\;\min{\left\|Z_{T+1}-\left(Z_{T}+\sum_{i=0}^{k-1}\text{Θ}(Z_{T+ih})\text{Ξ}h\right)\;\right\|}_{2}^{2},\min{\text{Ξ}}_{0}, \end{array}$$

where $Z_{T\;+\;ih}=Z_{T+(i-1)h}+\Theta \left(Z_{T+(i-1)h}\right)\Xi h$.

To achieve $\ell_{0}$ optimization, we perform pruning with $\ell_{2}$ regularization which is known to approximate $\ell_{0}$ regularization under regularity conditions (68, 84, 85). Applying SINDy unit has the following benefits: a) The SINDy-function library contains frequently used functions in physics modeling (e.g. polynomials and Fourier series). b) With sparse system identification, the neural network is more likely to identify governing physics, which is fundamentally important for extrapolation and long-term stability.

#### 2.2.2. Latent space regularization via ensemble SINDy.

We first note that we deviate from the original SHRED architecture by using a GRU as opposed to an LSTM. This choice was made because we generally found that GRU provides a smoother latent space and propagates only a single hidden state. Further details are provided in *SI Appendix*.

Our goal is to find a SHRED model with a latent state that is within the SINDy-class functional. However, there is no guarantee that the initial latent representation from SHRED will follow any ODE structure. On the one hand, if we naively apply SINDy to the initial latent representation, the discovery is unlikely to fit the latent representation trajectory. On the other hand, if we directly replace the GRU unit to SINDy and force the latent space to follow the discovered SINDy model, it might lose information spatial domain reconstruction. Therefore, it is important to let the two latent spaces align smoothly and progressively.

In *SI Appendix*, Algorithm 1, we describe our training procedure that allows the two trajectories to progressively align with each other. To further ensure a smooth alignment and avoid overregularization, we introduce ensemble SINDy units with varying levels of sparsity constraints, ranging from a full model (all terms in the library are active) to a null model (where no dynamics are represented). From the initial latent representation $Z^{\text{iter}\text{0}}$ from SHRED, the SINDy model first provides an initial estimate of an ensemble of SINDy coefficients ${\left\{{\hat{\Xi }}^{b}\right\}}_{b=1}^{N_{b}}$ with $N_{b}$ members. Then, the parameters of SHRED will be updated toward the dynamics simulated by ${\left\{{\hat{\Xi }}^{b}\right\}}_{b=1}^{N_{b}}$ learned from the first iteration, which generates a new latent representation trajectory $Z^{\text{iter}\text{1}}$. We iterate this procedure and jointly optimize the following loss function to let the SHRED latent representation trajectory approximate the SINDy generated trajectory:

[3] $$\begin{array}{cc} L= & {∥X_{L+1:T}-f_{\theta_{D}}\left(f_{\theta_{\text{GRU}}}\left(S_{L+1:T}\right)\right)∥}_{2}^{2}+\lambda {∥\Xi ∥}_{0}\\ & +\sum_{b=1}^{N_{b}}{∥Z_{T+1}^{\text{GRU}}-\left(Z_{T}^{\text{GRU}}+\sum_{i=0}^{k-1}\Theta \left(Z_{T+ih}\right)\Xi^{\left(b\right)}h\right)∥}_{2}^{2}, \end{array}$$

where $X_{L+1:T}={\left\{X_{i}\right\}}_{i=L+1}^{T}$ and $S_{L+1:T}={\left\{s_{t_{i-L}:t_{i}}\right\}}_{t_{i}=L+1}^{T}$. Here, $Z_{T+ih}=Z_{T}+\Theta \left(Z_{T+(i-1)h}\right)\Xi h$, $h=\frac{\Delta t}{k}$, and $Z^{\text{GRU}}$ denotes the GRU-inferred latent space computed from the real observation data. We note here that during neural network training, the total loss is computed as the summation of L over the entire dataset ${\left\{X_{i}\right\}}_{i=1}^{T}$.

#### 2.2.3. Latent space linearization via the Koopman operator.

Koopman operator theory (86, 87) provides an alternative approach to solving these problems by linearizing the underlying dynamics. The linearized embedding is theoretically grounded to be able to represent nonlinear dynamics in a linear framework, which is desirable for many applications in science and engineering, including control, robotics, weather modeling, and so on. From the transformed measurements z=g(X) from the true system X, the Koopman operator K is an infinite-dimensional linear operator given by Kg:=g°F where F(·) describes the law of the dynamical system in its original space that $X_{t+1}=F\left(X_{t}\right)$. The Koopman operator enables a coordinate transformation from X to a linearized latent space with dynamics $Kg\left(X_{t}\right)=g\left(X_{t+1}\right)$.

However, it is generally impossible to obtain the exact form of this infinite-dimensional operator, so a typical strategy is to find a finite-dimensional approximation of the Koopman operator by means of data-driven approaches (9). In Koopman-SHRED, we utilize the GRU unit to approximate the eigenfunctions. In the latent space, we enforce and learn the linear dynamics, represented by a matrix K, which corresponds to the latent space evolution derived from the eigenfunctions. We keep the interpretability of the model via a parsimonious latent space. In implementation, a simple strategy is to adapt the SINDy-unit with only the linear terms. This models a continuous version of Koopman generator that $\frac{d}{\mathrm{dt}}z\left(x\left(t\right)\right)=G_{t}z\left(x\left(t\right)\right)$ where the corresponding Koopman operator $K_{t}=e^{tG}$. Then, we follow a similar practice in SINDy-SHRED, updating the Koopman loss function:

$$\begin{array}{c} L={∥X_{L+1:T}-f_{\theta_{D}}\left(f_{\theta_{\text{GRU}}}\left(S_{L+1:T}\right)\right)∥}_{2}^{2}+{∥Z_{T+1}^{\text{GRU}}\;-\;KZ_{T}^{\text{GRU}}∥}_{2}^{2}. \end{array}$$

To enable continuous spectrum for better approximation for the Koopman operator, one could adapt an additional neural network to learn the eigenvalues $\lambda_{i}$’s and form the linear dynamics *K* from the learned eigenvalues (23). We also provide detailed implementation and experimental results in *SI Appendix*. This flexibility allows the system to learn dynamical systems in a more general setting, accommodating various initial conditions and experimental setups. In our experimental study, we observe that this strategy may perform comparably to learning a fixed K for linear dynamics under fixed experimental environments.

To summarize, the principal advantage of Koopman-SHRED over SINDy-SHRED lies in its enforcement that the latent dynamics of the recurrent unit are approximately linear. This linearity enables stability analysis and simulation-free predictions. However, this constraint also limits the expressive capacity of the learned model, and in some cases, the flexibility of a nonlinear representation remains essential for accurate modeling. As a practical guideline, Koopman-SHRED should be employed when its performance is adequate, with SINDy-SHRED serving as a more expressive alternative when additional modeling complexity is required.

### 2.3. Theoretical Analysis on Dynamical System Learning.

Suppose that the dynamical system has the form $\dot{z}=f\left(z\right)$, and we have measurements of $z=\{z_{1},z_{2},...,z_{t},z_{t+1},...,z_{T}\}$ with time gap Δt. The empirical loss function $L_{n}\left(\cdot \right)$ of a function *f* given the empirical distribution $P_{n}$ which consists of data samples $D={\left\{\left(z_{i},\dot{z_{i}}\right)\right\}}_{i=1}^{n}$ is $L_{n}\left(f\right)=\frac{1}{n}\sum_{i=1}^{n}\ell \left(f\left(z_{i}\right),z_{i}\right)$. The true loss is $L\left(f\right)=E\left[\ell \left(f(z),y\right)\right]$.

From empirical process theory, there is a generalization gap between $L_{n}\left(f\right)$ and L(f), which is correlated with the expressive power of the functional class (88, 89). When the expressive power of the estimator is excessive, we expect that local perturbations can cause significant shifts in performance, leading to a larger generalization error. Therefore, although neural networks are universal approximators (90), they can perform poorly in extrapolation. SINDy effectively avoids this issue because the functional class is moderately sized, which will help to control the generalization error. Additionally, the continuous formulation of SINDy also enables prediction at arbitrary temporal resolutions. It allows physically meaningful forecasts even beyond the sampling rate of the training data.

The following theorems (assumptions in *SI Appendix*) present the error bounds for dynamical system learning with the SINDy-class and the neural network class respectively. We consider Gaussian residuals on z to reflect independent measurement noise commonly arising from sensing devices.

Theorem 1. *For a SINDy-class model with a library of functions* Θ(z)*, and we estimate the coefficient vector* *ξ* *by* $\hat{\xi }$ *using least squares. Let* $\xi^{*}$ *be the true coefficient vector. Under the assumptions in the SI Appendix, we have the expected error in predicting the dynamical system after time* *T* *is bounded by* $E||\hat{z}\left(T\right)-z\left(T\right)\left|\right|\leq O\left(e^{\mathrm{LT}}s\sqrt{\frac{p}{n}}\right)$ *where* *L* *is the Lipschitz constant of the system,* *s* *is the level of noise,* *p* *is the number of functions in the library, and* *n* *is the number of samples.*

*Furthermore, with probability* 1−δ*, the error in predicting the dynamical system after time* *T* *is bounded by*

[4] $$\begin{array}{c} \left|\right|\hat{z}\left(T\right)-z\left(T\right){\left|\right|}_{2}\leq O\left(e^{\mathrm{LT}}s\sqrt{\frac{p}{n}\log\left(\frac{1}{\delta }\right)}\right). \end{array}$$

Theorem 2. *For a neural network model with* *k* *layers of ReLU activation functions and parameters* $\theta =\left(W_{1},\cdots ,W_{k}\right)$*, which computes functions* $f\left(x;\;\theta \right)=\sigma_{k}\left(W_{k}\sigma_{k-1}\left(W_{k-1}\cdots \sigma_{1}\left(W_{1}x\right)\right)\right)$.

*Under the assumptions in* *SI Appendix**, with probability at least* 1−δ*, the error in predicting the dynamical system after* H=T/Δt *steps is bounded by*

$$\begin{array}{c} E\left[\left|\right|\hat{z}\left(T\right)-z\left(T\right){\left|\right|}_{2}^{2}\right]\leq O\left({\left(\log n\right)}^{4}B^{k(H+1)}\sqrt{\frac{k}{n}}+\frac{\log(1/\delta )}{n}\right), \end{array}$$

*where* *n* *is the number of samples.*

From the error bounds above, we notice that compared to traditional learning problems (e.g. classification), dynamical system learning is more challenging as the error grows exponentially with increasing time horizon even in linear settings. From the error bound, it becomes evident why neural networks may encounter challenges. First, for the constant term $B^{\mathrm{kH}}$, the norm of weights *B* and the number of layers *k* are expected to increase significantly for complex systems. Second, the rate of convergence of neural network learning is generally slower than that of linear least-squares methods ($n^{-\frac{1}{4}}$ vs. $n^{-\frac{1}{2}}$), which requires substantially more training samples. Moreover, the theoretical bounds on neural networks can primarily control the expected trajectory error, but has a limited route to bound the supremum error (as in *Theorem* 1), which can quickly amplify and lead to divergence in dynamical system predictions. The usage of popular transformer networks tends to memorize instead of learning the mechanism, which can produce poor extrapolations from unseen initial conditions (91–93).

## 3. Computational Experiments

In the following, we perform case studies across a range of scientific and engineering problems. We begin with building models directly from video data, which includes flow around a cylinder and a pendulum. We then consider real-world sea-surface temperature data and conclude with turbulent flow examples. The experiments are all executed on a Linux system with a single NVIDIA GeForce RTX 2080 Ti GPU.

### 3.1. GoPro Physics Video Data: Flow Over a Cylinder.

In this subsection, we demonstrate the performance of SINDy-SHRED on an example of so-called “GoPro physics modeling.” The considered data are collected from a dyed water channel to visualize a flow over a cylinder (94). The Reynolds number is 171 in the experiment. The dataset contains 11 s of video taken at 30 frames per second (FPS). We transfer the original RGB channel to gray scale and remove the background by subtracting the mean of all frames. After the prior processing step, the video data have only one channel (gray) within the range (0,1) with a height of 400 pixels and a width of 1,000 pixels. We randomly select and fix 200 pixels as sensor measurements from the entire 400,000 space, which is equivalent to only 0.05% of the data. We set the lag parameter to 60 frames. We include the details of the experimental settings of the dataset and SINDy-SHRED in *SI Appendix* (Page 17).

#### 3.1.1. SINDy-SHRED discovery.

We define the representation of the latent state space as $\left(z_{1},z_{2},z_{3},z_{4}\right)$. We discover the following dynamical system:

[5] $$\begin{array}{c} \left\{\begin{array}{cc} {\dot{z}}_{1} & =-0.69z_{2}+0.98z_{3}-0.40z_{4},\\ {\dot{z}}_{2} & =1.00z_{1}-0.78z_{3}1-0.31z_{2}z_{3}^{2},\\ {\dot{z}}_{3} & =-1.029z_{1}+0.59z_{2}+0.41z_{4}.\\ {\dot{z}}_{4} & =-0.26z_{1}^{2}-0.29z_{2}^{2}z_{3}-0.39z_{3}^{3}. \end{array}\right. \end{array}$$

The identified nonlinear system has two fixed points, z=0 and $z={\left(\begin{array}{cccc} -0.11 & -0.23 & -0.14 & 0.05 \end{array}\right)}^{T}$ which are unstable.

#### 3.1.2. Koopman-SHRED discovery.

We define the latent states as $\left(z_{1},z_{2},z_{3},z_{4},z_{5},z_{6}\right)$. We discover the following dynamics:

[6] $$\begin{array}{c} \left\{\begin{array}{cc} {\dot{z}}_{1} & =-0.920z_{4}+0.620z_{5},\\ {\dot{z}}_{2} & =-0.163z_{1}+0.817z_{4}+0.778z_{6},\\ {\dot{z}}_{3} & =-0.462z_{4}-1.026z_{6},\\ {\dot{z}}_{4} & =1.791z_{1}+0.307z_{6},\\ {\dot{z}}_{5} & =-0.969z_{1}+0.548z_{6},\\ {\dot{z}}_{6} & =-0.800z_{2}-0.915z_{5}. \end{array}\right. \end{array}$$

The analytic solution has the form

$$\begin{array}{cc} z(t)= & c_{1}v_{1}\cos\left(\omega_{1}t\right)e^{-\lambda_{1}t}+c_{2}v_{2}\sin\left(\omega_{1}t\right)e^{-\lambda_{1}t}\\ & +c_{3}v_{3}\cos\left(\omega_{3}t\right)e^{\lambda_{3}t}+c_{4}v_{4}\sin\left(\omega_{3}t\right)e^{\lambda_{3}t}+c_{5}v_{5}e^{-\lambda_{5}t}, \end{array}$$

where $\omega_{1}=1.52$, $\omega_{3}=1.05$, $\lambda_{1}=0.01$, $\lambda_{3}=0.11$, and $\lambda_{5}=-0.20$. The explicit solution is given in *SI Appendix*.

**Fig. 2.**
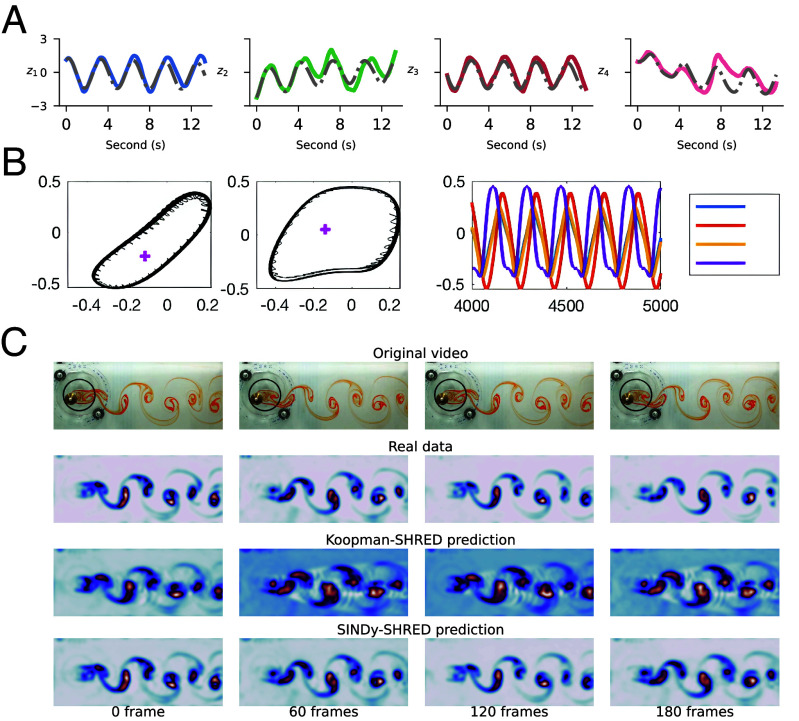
(*A*) Extrapolation of latent representation in SINDy-SHRED from the discovered dynamical system for flow over a cylinder data. Colored: latent representation. Gray: SINDy extrapolation from derived latent dynamical system. (*B*) Evolution of dynamical system ((Eq. **5**)). The *Left* two panels are the phase plane $z_{1}$ vs. $z_{2}$ and $z_{3}$ vs. $z_{4}$ respectively. The magenta plus symbols are the fixed points. The nonlinear limit cycle behavior is shown in the *Right* panel for $z_{j}\left(t\right)$. (*C*) Long-term pixel space video prediction via SINDy-SHRED. We demonstrate the forward prediction outcome up to 180 frames.

The flow over a cylinder model contains both linear and nonlinear interactions. In Eq. **5**, we find that $z_{1}$ and $z_{3}$ behave like a governing mode of the turbulence swing; $z_{2}$ and $z_{4}$ further depict more detailed nonlinear effects. We further present a linear model derived from Koopman-SHRED in Eq. **6** with its latent space evolution demonstrated in *SI Appendix*, Fig. S22. We show the result of extrapolating this learned representation. We generate the trajectory from the initial condition at time point 0 and perform forward integration for extrapolation. As shown in Fig. 2*A*, the learned ODE closely follows the dynamics of $z_{1}$ and $z_{3}$ up to around 7 s, where the relative mean-squared error (rMSE) remains below 0.1%; $z_{2}$ and $z_{4}$ also have close extrapolation up to 4 s with rMSE remains below 0.1%. Fig. 2*B* shows that the learned nonlinear dynamical system. The discovered latent ODE exhibits a closed limit cycle as demonstrated in *SI Appendix*, Fig. S22. The Koopman-SHRED approximation aligns with the overall trend for each latent coordinate but exhibits phase and amplitude drift.

This learned representation nicely predicts the future frames in pixel space. In SINDy-SHRED, the shallow decoder prediction has an averaged MSE error of 0.030 over the entire available trajectory. Koopman-SHRED has relatively worse prediction error with an averaged MSE error of 0.053. In Fig. 2*C*, we observe that the autoregressively generated prediction frames follow the same temporal progression and vortex patterns as the true data. Furthermore in *SI Appendix*, Fig. S24, we find that the long-term predictions can preserve the vortex pattern without collapsing to an averaged static frame after 1,000 steps, which is out of the size of the original dataset. The sensor-level prediction in *SI Appendix*, Fig. S23 further demonstrates the accuracy of reconstruction in detail.

### 3.2. Prediction and Baseline Study of Single Shot Pendulum Video.

**Fig. 3.**
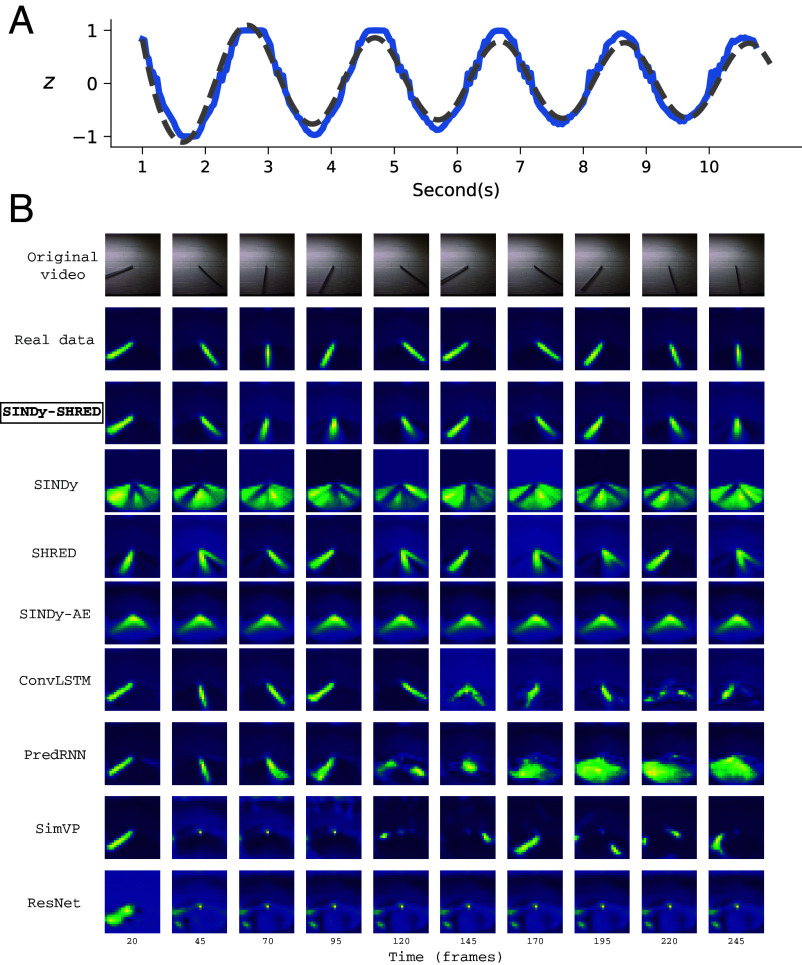
(*A*) Extrapolation of latent representation in SINDy-SHRED from the discovered dynamical system for the pendulum moving data. Blue: true latent representation. Gray: SINDy extrapolation. (*B*) The pendulum video generation outcome from ResNet, SimVP, ConvLSTM, PredRNN, SINDy, SHRED, SINDy-autoencoder, and SINDy-SHRED from frame 20 to frame 245.

In this subsection, we compare the performance of SINDy-SHRED to existing state-of-the-art learning algorithms. In the following, we demonstrate the result of video prediction on the pendulum data using ResNet (52), convolutional LSTM (convLSTM) (55), and PredRNN (56), and SimVP (57). The pendulum in our experiment is not ideal and includes complex damping effects. We use a nail on the wall and place the rod (with a hole) on the nail. We include the details of the experimental settings of the dataset and SINDy-SHRED in *SI Appendix* (Page 17). This creates complex friction, which slows the rod more when passing the lowest point due to the increased pressure caused by gravity. The full model we discovered from the video (shown in Fig. 3) is given by

[7] $$\begin{array}{c} \ddot{z}=0.17{\dot{z}}^{2}-0.06{\dot{z}}^{3}-10.87\sin\left(z\right)+0.48\sin\left(\dot{z}\right), \end{array}$$

which includes complex damping effects via $\sin\left(\dot{z}\right),{\dot{z}}^{2},{\dot{z}}^{3}$, in contrast to the commonly assumed linear damping.

As shown in Table 1, SINDy-SHRED outperforms all baseline methods for total error and long-term predictions. Generally, all baseline deep learning methods perform well for short-term forecasting, but the error quickly accumulates for longer-term predictions. This is also observable from the prediction in the pixel space as shown in Fig. 3*B*. SINDy-SHRED is the only method that does not produce collapsed longer-term predictions. Interestingly, we observe that incorporating nonlinear terms in the SINDy library is crucial in this case, as Koopman-SHRED struggles to reconstruct the finer details. We attribute this to the fact that strong linear regularization may overregularize and oversimplify the complex dynamics, leading to a reduction in predictive accuracy. Similar behaviors for Koopman-SHRED are observed when encoding the continuous spectrum using an additional shallow network. In *SI Appendix*, Fig. S27, the sensor level prediction also demonstrates the robustness of the SINDy-SHRED prediction. PredRNN is the second best method as measured by the total error. However, PredRNN is expensive in computation which includes a complex forward pass with an increased number of parameters. It is also notable that the prediction of PredRNN collapses after 120 frames, after which only an averaged frame over the entire trajectory is predicted. ConvLSTM has a relatively better result in terms of generation, but the long-term prediction it still inferior compared to SINDy-SHRED. Additionally, it should be noted that 2D convolution is much more computationally expensive. For larger spatiotemporal domains (e.g. the SST example and 3D ozone data), the direct combination of 3D convolution and recurrent layers become computationally demanding (95), as both memory and runtime scale cubically with spatial resolution and linearly with temporal history (96). Similar computational issues will occur for diffusion models as a denoising diffusion probabilistic model can take 20 h to sample 50 K images of size 32×32 (97). For scientific datasets that easily reach terabyte scale, these methods can become computationally intensive and challenging to scale to long-horizon, high-resolution scientific applications (98–103). By constraining the library to be linear, a SINDy model could predict the high-dimensional system forward. However, it is inaccurate for short- and long-term forecasts, and loses interpretability due to the required large number of parameters. SHRED also struggles with long-term rollouts, as it is primarily focused on sparse sensing problems rather than full spatiotemporal modeling. SINDy-AE often converges to an incorrect latent dynamical model, leading to degraded rollout performance. The detailed training and model configurations are provided in *SI Appendix*. In summary, we observe that SINDy-SHRED is not only producing long-term predictions with smaller mean squared error, but is also fast to execute and smaller in size.

**Table 1.** Comparison table of SINDy-SHRED to baseline methods for parameter size, training time, and error over different time horizons

Models	Params	Training time	T=[0,100]	T=[100,200]	T=[200,275]	Total
SINDy (linear) (82)	420 K	8 min	$4.04\times 10^{-2}$	$4.20\times 10^{-2}$	$4.21\times 10^{-2}$	$4.14\times 10^{-2}$
SHRED (2)	240 K	8 min	$1.24\times 10^{-2}$	$2.08\times 10^{-2}$	$2.83\times 10^{-2}$	$1.66\times 10^{-2}$
SINDy-AE (22)	200 K	6 min	$1.69\times 10^{-2}$	$1.51\times 10^{-2}$	$1.52\times 10^{-2}$	$1.58\times 10^{-2}$
ResNet (52)	2.7M	24 min	$2.08\times 10^{-2}$	$1.88\times 10^{-2}$	$2.05\times 10^{-2}$	$2.00\times 10^{-2}$
SimVP (57)	460 K	30 min	$2.29\times 10^{-2}$	$2.47\times 10^{-2}$	$2.83\times 10^{-2}$	$2.53\times 10^{-2}$
PredRNN (56)	444 K	178 min	$1.02\times 10^{-2}$	$1.79\times 10^{-2}$	$1.69\times 10^{-2}$	$1.48\times 10^{-2}$
ConvLSTM (55)	260 K	100 min	$9.24\times 10^{-3}$	$1.86\times 10^{-2}$	$1.99\times 10^{-2}$	$1.55\times 10^{-2}$
SINDy-SHRED*	44 K	17 min	$1.70\times 10^{-2}$	$9.36\times 10^{-3}$	$5.31\times 10^{-3}$	$1.05\times 10^{-2}$

### 3.3. NOAA Sea-Surface Temperature Data.

The third example we consider is that of global sea-surface temperature. The SST data contain 1,400 weekly snapshots of the weekly mean sea surface temperature from 1992 to 2019 reported by NOAA (104). The reanalysis dataset combines in situ and satellite data through optimal interpolation, which is represented by a 180×360 grid. Within these grids, 44,219 of its grid points correspond to sea-surface locations. We standardize the data with its own min and max, which transforms the sensor measurements to within the numerical range of (0,1). We randomly select 250 sensors from the possible 44,219 locations and set the lag parameter to 52 wk. Thus, for each input–output pair, the input consists of the 52-wk trajectories of the selected sensors, while the output is a single temperature field across all 44,219 spatial locations. We include the further details of the dataset and the experimental settings of SINDy-SHRED in *SI Appendix* (Page 17). From the discovered coordinate system, we define the latent representation of physics to be $\left(z_{1},z_{2},z_{3}\right)$. The dynamics evolves via the following set of equations:

[8] $$\begin{array}{c} \left\{\begin{array}{cc} {\dot{z}}_{1} & =4.68z_{2}-2.37z_{3},\\ {\dot{z}}_{2} & =-3.10z_{1}+3.25z_{3},\\ {\dot{z}}_{3} & =2.72z_{1}-5.55z_{2}, \end{array}\right. \end{array}$$

where the analytic solution to this system of ODEs is

$$\begin{array}{c} z\left(t\right)=c_{1}v_{1}\cos\left(\omega_{1}t\right)e^{-\lambda_{1}t}+c_{2}v_{2}\sin\left(\omega_{1}t\right)e^{-\lambda_{1}t}+c_{3}v_{3}e^{\lambda_{3}t}. \end{array}$$

The value of $\omega_{1}=1.99\pi$, approximately corresponding to the expected period of 1 y. $\lambda_{1}=0.00763$ indicating a slow decay of the oscillatory mode with half-life 90.84 y. $\lambda_{3}=0.01527$ indicating global increases in temperature with doubling time 45.39 y. The explicit solution is given in *SI Appendix* (Page 25).

**Fig. 4.**
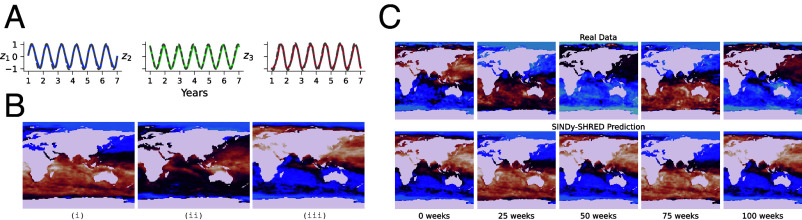
(*A*) Extrapolation of latent representation in SINDy-SHRED from the discovered dynamical system for SST. Colored: true latent representation. Gray: SINDy extrapolation. (*B*) Decoder reconstruction of three independent directions $z_{1},z_{2},z_{3}$ in the latent space. (*C*) Long-term global sea-surface temperature prediction via SINDy-SHRED from week 0 to week 100. We crop the global map for better visualization.

The discovery of a linear system describing the evolution of the latent state is in line with prior work on SST data (105) in which it was assumed that that the underlying physics is an advection-diffusion PDE. In Fig. 4*A* we further present the accuracy of this discovered system by forward simulating the system from an initial condition for a total of 27 y in *SI Appendix*, Fig. S17. It is observable how the discovered law is close to the true evolution of latent states and, critically, there appears to be minimal phase slipping. By extrapolating the latent state space via forward integration, we can apply the shallow decoder to return forecasts of the future spatial domain. We denormalize the temperature values during evaluation with temperature ranges from (−1.8,35.62). Doing so, we find an averaged MSE error of 0.57 ± 0.10 for 318 wk in the test dataset. This corresponds to a mean absolute error of 0.75 °C for each sensor points with relative error 2.01%. The eigenmodes of the SST data are visualized by feeding unit vectors along the independent latent directions $z_{1},z_{2},z_{3}$ into the decoder in Fig. 4*B*. In Fig. 4*C*, we show SINDy-SHRED produces stable long-term predictions for SST data. We further include *SI Appendix*, Fig. S18 to demonstrate the extrapolation from randomly sampled sensor points and *SI Appendix*, Fig. S9 to show the error evolution with time. The sensor level prediction is based on the global prediction of the future frame, and we visualize the signal trajectory of randomly sampled testing sensor locations. We find SINDy-SHRED is robust for out-of-distribution sensors, with reasonable extrapolations even in the presence of anomalous events.

### 3.4. Predicting Isotropic Turbulence Flow.

The isotropic turbulence flow data are a data simulation from the pressure field of a forced isotropic turbulent flow. We obtain 2D snapshots of the original pressure field data in refs. 106 and 107

[9] $$\begin{array}{c} \nabla^{2}\left(p/\rho \right)=\left(\Omega -\epsilon /\nu \right)/2, \end{array}$$

where *p* is the pressure, *ρ* is the density of the fluid, Ω is the vorticity, *ϵ* is the dissipation rate, and *ν* is the kinematic viscosity. The flow was generated with $1,024^{3}$ nodes with the Taylor-scale Reynolds number fluctuating around 433. The pressure field provides a scalar representation that captures the essential multiscale dynamics while simplifying analysis compared to vector velocity fields. We process the data to shrink the spatial resolution to 350×350, while keeping the time step to be 0.002. We standardize the data within the range of (0,1) with 50 sensors out of 122,500 grid points (0.4%). We include details of the dataset and SINDy-SHRED setup in *SI Appendix* (Page 17).

Our approximation of the latent representation has 8 oscillatory modes, and permits the following analytic solution:

[10] $$\begin{array}{cc} z(t)= & \sum_{i=1}^{7}\left(c_{2i-1}v_{2i-1}\cos\left(\omega_{i}t\right)e^{\lambda_{i}t}+c_{2i}v_{2i}\sin\left(\omega_{i}t\right)e^{\lambda_{i}t}\right)\\ & +c_{15}v_{15}e^{\lambda_{8}t}+c_{16}v_{16}e^{\lambda_{9}t}, \end{array}$$

where $\omega_{1}=9.39$, $\lambda_{1}=0.47$, $\omega_{2}=11.90$, $\lambda_{2}=0.05$, $\omega_{3}=13.42$, $\lambda_{3}=0.03$, $\omega_{4}=3.46$, $\lambda_{4}=-0.04$, $\omega_{5}=5.44$, $\lambda_{5}=-0.27$, $\omega_{6}=8.30$, $\lambda_{6}=-0.75$, $\omega_{7}=18.72$, $\lambda_{7}=1.37$, $\lambda_{8}=-0.26$, $\lambda_{9}=-3.39$, with coefficients $c_{i}$’ and eigenvectors $v_{i}$ (*SI Appendix*). Since the model is learned from a local 2D snapshot of a larger flow, positive eigenvalues reflect local amplification and do not imply instability of the full statistically stationary system.

**Fig. 5.**
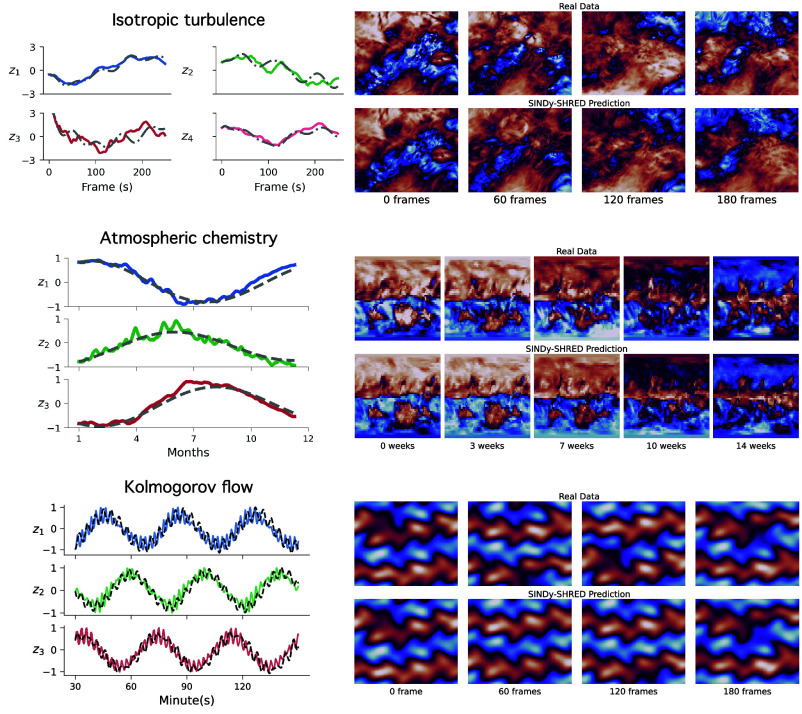
Visualizations of latent spaces and predictions for SINDy-SHRED applied to PDE simulations of isotropic turbulence, atmospheric chemistry, and 2D Kolmogorov flow datasets. *Left*: Extrapolation of latent representations using SINDy-SHRED from the discovered dynamical system for the ozone data, showing the true latent representation (colored lines) and SINDy extrapolation (gray lines). *Right*: Long-term spatiotemporal predictions from SINDy-SHRED compared to ground truth. The details of the 2D Kolmogorov experiment can be found in *SI Appendix*.

The isotropic turbulent flow exhibits chaotic behavior which makes the prediction challenging. However, a linear model still captures the governing dynamics nicely and provides a stable prediction for approximately 200 frames. In Eq. **10**, we find three increasing oscillatory modes and five decreasing oscillatory modes. For pixel space prediction, SINDy-SHRED prediction has an averaged MSE error of 0.03 over the entire dataset. In Fig. 5, we find that the predictions have small MSE up to 180 frames, which closely follows the governing trend of turbulent flow. The sensor-level prediction in *SI Appendix*, Fig. S26 further demonstrates the details of the signal prediction. Three of the oscillatory modes demonstrate growing trends. Four of the oscillatory modes decay, as well as the exponential modes. We note here that due to the complex and chaotic nature of the data, accurate sensor-level prediction is particularly challenging. Despite this complexity, the SINDy-SHRED model still provides a stable extrapolation of the overall trends.

### 3.5. 3D Atmospheric Ozone Concentration.

The atmospheric ozone concentration dataset (108) contains a one-year simulation of the evolution of an ensemble of interacting chemical species through a transport operator using GEOS-Chem. The simulation contains 1,456 temporal samples with a timestep of 6 h over one year for 99,360 (46 by 72 by 30) spatial locations (latitude, longitude, elevation). The data presented in this work has compressed by performing an SVD and retaining only the first 50 POD modes. As with the SST data, we standardize the data within the range of (0,1) and randomly select and fix 3 sensors out of 99,360 spatial locations (0.5%). We include the details of the experimental settings of the dataset and SINDy-SHRED in *SI Appendix* (Page 17). The converged latent representation gives

[11] $$\begin{array}{c} \left\{\begin{array}{cc} {\dot{z}}_{1} & =-0.002-0.013z_{2}+0.007z_{3},\\ {\dot{z}}_{2} & =-0.001z_{1}+0.004z_{2}-0.008z_{3},\\ {\dot{z}}_{3} & =0.002+0.012z_{2}-0.005z_{3}. \end{array}\right. \end{array}$$

The analytic solution to this system of ODEs has the form

$$\begin{array}{cc} z(t)= & c_{1}v_{1}\cos\left(\omega_{1}t\right)e^{-\lambda_{1}t}+c_{2}v_{2}\sin\left(\omega_{1}t\right)e^{-\lambda_{1}t}+c_{3}v_{3}e^{-\lambda_{3}t}\\ & +\int_{0}^{t}\left(c_{4}v_{1}\cos\left(\omega_{1}t\right)e^{-\lambda_{1}t}+c_{5}v_{2}\sin\left(\omega_{1}t\right)e^{-\lambda_{1}t}+c_{6}v_{3}e^{-\lambda_{3}t}\right)d\tau , \end{array}$$

where $\omega_{1}=0.0079,$$\lambda_{1}=0.003$, and $\lambda_{3}=0.003.$ The complete solution is given in *SI Appendix*, Eq. **S64**.

Unlike traditional architectures for similar problems, which includes expensive operations such as the 3D convolution, SINDy-SHRED provides an efficient way of training. Although the quantity of data is insufficient to perform long term-predictions, SINDy-SHRED still exhibits interesting behavior for a longer-term extrapolation which converges to the fixed point at 0 (as shown in *SI Appendix*, Fig. S19). From the extrapolation of the latent state space, the shallow decoder prediction has an averaged MSE error of $1.5e^{-2}$. In Fig. 5, we visualize the shallow decoder prediction up to 14 wk. In Fig. 5, we reconstruct the sensor-level predictions. The observations are much noisier than the SST data, but SINDy-SHRED provides a smoothed extrapolation.

### 3.6. Visualizing the Convex Loss Landscape.

**Fig. 6.**
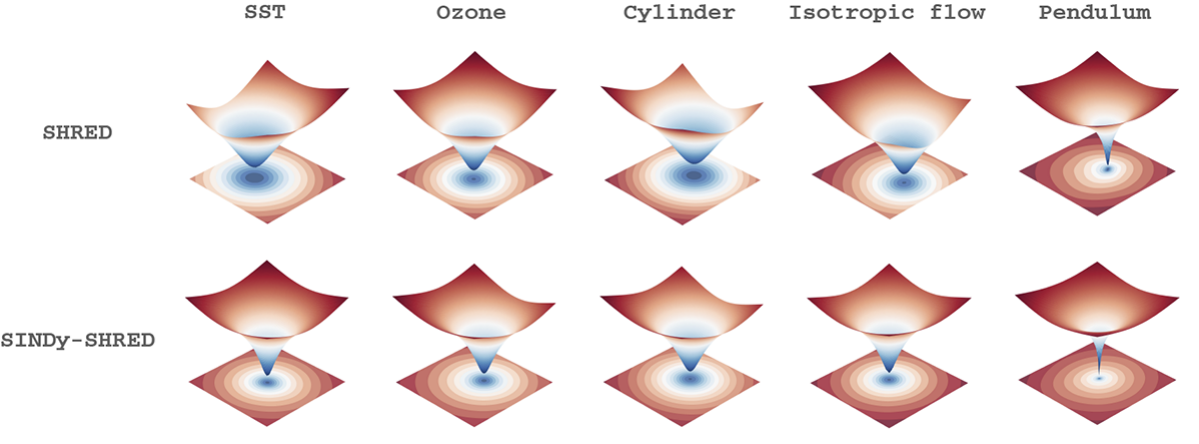
Loss landscape plot of SHRED (*Top* row) and SINDy-SHRED (*Bottom* row) for all datasets presented: sea-surface temperature, Ozone, flow over a cylinder, isotropic flow, and pendulum.

We visualize the loss landscape for each experiment in Fig. 6. Following the methodology of ref. 11, this visualization demonstrates the loss landscape along two random directions. By perturbing the model weights along these directions, we obtain a 3D visualization of the loss surface. We observe that, for both SHRED and SINDy-SHRED, the loss landscape is surprisingly smooth and exhibits perturbative convexity, featuring a clear global minima. The visualizations across all datasets consistently demonstrate these favorable convex loss landscapes. In the Appendix, we further visualize different settings of SHRED under various random directions and scales in *SI Appendix*, Figs. S2 and S3. From these optimization landscapes, we observe that the SHRED architecture encourages flat minimizers and reduces the potential for chaotic training behavior. This property also provides SHRED with properties including hyperparameter stability, reliable statistical inference, and guaranteed data recovery.

## 4. Discussion and Conclusion

In this paper, we present the SINDy-SHRED algorithm, which jointly solves the sensing and model identification problem with simple implementation, efficient computation, and robust performance. Specifically, SINDy-SHRED (Koopman-SHRED) learns a latent coordinate system for which parsimonious, low-dimensional governing equations, either linear or nonlinear, can be constructed. The SINDy-SHRED model has strong predictive capabilities and stable roll outs by identifying the underlying physics. Through numerous experiments on challenging real-world data, we show that our method can produce robust and accurate long-term predictions for a variety of complex problems, including global sea-surface temperature, turbulent flows, and videos of physics, which includes recordings of flow over a cylinder and a moving pendulum. The simplicity and robustness of the architecture, along with its performance on real videos and measurements, make it highly compelling as a new paradigm for learning physics models directly from data.

Learning new physics and obtaining new insights from well-established physics problems is challenging, especially given the extensive efforts and long-standing interest from the broader community. Despite this, SINDy-SHRED has been demonstrated to derive new physics models for every single example considered in this paper. For instance, directly from video, SINDy-SHRED identified two potential models to capture the flow around a cylinder: i) a four-dimensional nonlinear ODE system containing a limit cycle modeling the nonlinear oscillations induced by the von Karman vortex shedding, and ii) a linear six-dimensional Koopman model which can be solved in closed form. Both models are new to the field, a rarity given the decades of research devoted to understanding the bifurcation that leads to the vortex shedding dynamics. Moreover, the physics discovery is done solely from pixel space, a remarkable achievement that stands in contrast to previous efforts requiring access to the full PDE simulation fields. In the pendulum example, SINDy-SHRED learns a nonlinear damping term that captures the complex motion of the system, improving on the typical linear damping assumption. Beyond video data, SINDy-SHRED also demonstrates exceptional performance in modeling turbulent flows. Remarkably, the latent space dynamics reveal that eight modes suffice to accurately capture the dynamics of isotropic turbulence. Even global sea-surface temperature is captured with a three-dimensional linear ODE system that one might solve in a first course on ODEs. These results are astounding given the complex data, highlighting the ability of SINDy-SHRED to extract governing equations from well-known and important scientific problems.

In addition to learning interpretable new physics models, SINDy-SHRED is simple and robust to train. As shown above and in *SI Appendix*, this largely stems from the observed globally convex loss landscape, which is rare among all existing deep learning models as noted in ref. 11. In general, a major goal in designing deep learning models is to engineer the loss landscape to align with the inherent characteristics of the data, thereby facilitating stable training, robust hyperparameter tuning, and generalizable performance (109, 110). SINDy-SHRED naturally exhibits an ideal loss landscape that is globally convex, which is what the deep learning community strives to engineer. Although exploring the underlying mechanisms is beyond the scope of the current study, future work will investigate how the SINDy-SHRED architecture induces this ideal loss landscape. Moreover, SINDy-SHRED is grounded by rigorous convergence guarantees for the SINDy-class of models, offering theoretical advantages over competing neural network-based approaches. These theoretical results offer precise error bounds and the performance for long-term prediction. As a result, SINDy-SHRED is well grounded in theory, providing a solid foundation for further development and widespread practical applications.

Most importantly, in practice, SINDy-SHRED achieves state-of-the-art performance in long-term autoregressive video prediction, outperforming ConvLSTM, PredRNN, ResNet, and SimVP while maintaining the lowest computational cost, smallest model size and minimal training time simultaneously. Notably, SINDy-SHRED outperforms the leading methods on all metrics of interest without any hyperparameter tuning. Moreover, this performance is achieved while producing an interpretable model of the underlying dynamics, effectively capturing the actual governing physics, a contribution that competing methods do not offer. This enables stable, long-time rollouts of video prediction. Such performance advances the goal of GoPro Physics, which aims to discover governing equations directly from few-shot video data streams.

SINDy-SHRED yields interpretable latent dynamics, while using a data-driven surrogate to model the coordinate transformation. The GRU network approximates the mapping from sensor measurements to latent states with limited interpretability on its learned weights. We also note that SINDy-SHRED explicitly enforces the latent dynamics to follow a single ODE uniformly. If the true system exhibits switching dynamics, this assumption is violated unless appropriate regime separation or preprocessing is applied.

To conclude, the SHRED architecture and its variants have proven to be highly effective algorithms for sensing (2, 76), parametric model reduction (3–5) and now, as demonstrated in this work, physics discovery. The SINDy-SHRED and Koopman-SHRED architectures are robust, stable, and computationally efficient. This allows for laptop level computing along with afternoon-level tuning-even on exceptionally challenging datasets. Its ease of use and reliability position it as a compelling algorithm to be the new paradigm for general-purpose physics model discovery.

## Supplementary Material

Appendix 01 (PDF)

## Data Availability

The python implementation of SINDy-SHRED is available both on Github and pyshred package (111). The implementation includes the model architecture, training settings, and code to create plottings. All data are publicly available on Zenodo. All other data are included in the manuscript and/or *SI Appendix*.

## Footnotes

^*^Code implementation: https://github.com/gaoliyao/sindy-shred.
