# Supplementary material for: Sparse identification of nonlinear dynamics and Koopman operators with Shallow Recurrent Decoder Networks
Source: Proc Natl Acad Sci U S A. 2026 Apr 17;123(16):e2508144123. doi: 10.1073/pnas.2508144123 (PMC13099575; doi:10.1073/pnas.2508144123)
Supplement: Supplementary file 1 — Appendix 01 (PDF) [file pnas.2508144123.sapp.pdf]

Supporting Information for

Sparse identification of nonlinear dynamics and Koopman operators with Shallow Recurrent Decoder Networks

Mars Liyao Gao, Jan P. Williams, J. Nathan Kutz

J. Nathan Kutz. E-mail: kutz@uw.edu

This PDF file includes:

Supporting text
Figs. S1 to S28
Table S1
SI References

Supporting Information Text

Challenges in rolling out neural networks for fitting a simple sine function

In the following example, we consider a simple use case in which we fit a simple sine function using recurrent neural networks. Surprisingly, extrapolating a simple sine function can be a difficult task for neural networks. The dynamical system x is generated via the following equation:

$$\ddot{z} = -\sin(z). \quad [1]$$

We implement a GRU network in the following, which contains three stacked GRU layers with size 500, and a fully connected output layer. We employ the Adam optimizer with a learning rate of 0.001 and used the mean squared error (MSE) as the loss function. We train the GRU network with 150 epochs. The input sequences are normalized to [0, 1]. The SINDy library consists of 12 candidate functions and is denoted by $\Theta(x) = [1, z, \dot{z}, z^2, z\dot{z}, \dot{z}^2, z^3, z^2\dot{z}, z\dot{z}^2, \dot{z}^3, \sin(z), \sin(\dot{z})]$. We applya sequentially thresholded least-squares algorithm to perform sparsity promoting regression with threshold 0.5 and ℓ_2 regularization 0.05.

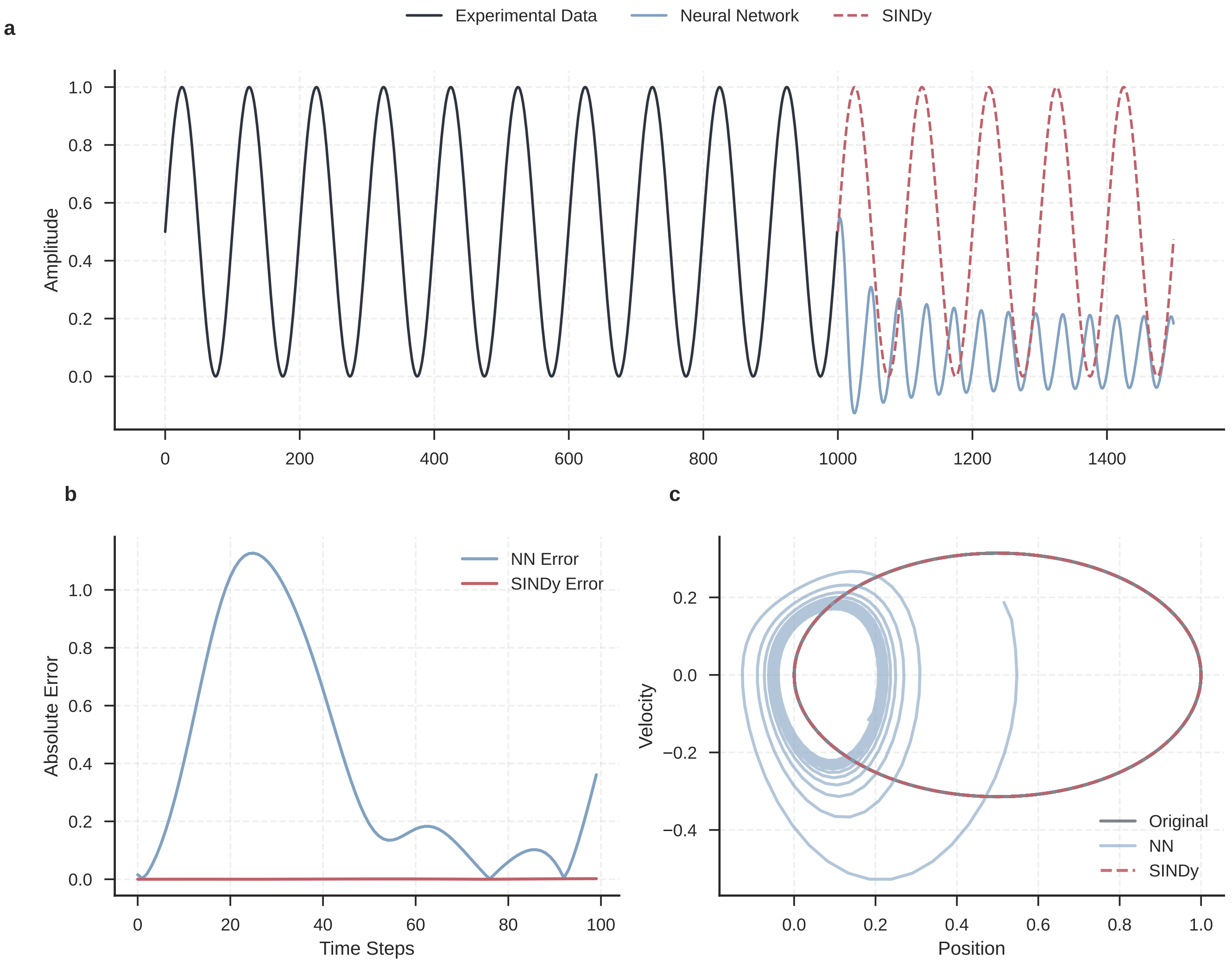

Figure S1. Fitting a simple sine function using SINDy and the GRU network.

From Fig. S1, we observe that SINDy approximation largely outperforms the GRU network. The extrapolation of GRU is poor due to the overly strong expressive power of this type of functional. We locate this to be a critical reason that most neural networks fail to capture physics nicely. Additionally, it is not clear for neural networks to estimate the dynamical system from the discrete setup $(t_0, t_1, ..., t_T)$ to a continuous one, while SINDy provides a natural transition into a continuous-time formulation. The SINDy prediction could also remarkably improve generalizability, which could enhance the performance in long-term prediction.

Theoretical justification on the generalization error between SINDy and the neural networks

We further establish the statistical foundation for dynamical system learning. When the underlying dynamical system can be closely described by a linear combination of the library of functions, obtaining a "governing equation" will have huge benefits for long-term extrapolation. yoDue to the nature of forward integration,error accumulates rapidly making an approximate system undesirable for extrapolation. In the following, we formalize this statement by analyzing the Rademacher complexity of SINDy-class and neural networks functional.

Theoretical setup. The system we wish to study has the form that

$$\dot{z}_t = f^*(z), \tag{2}$$

which is an ODE describing the trajectory of a dynamical system in a learned latent space and $f^*(\cdot)$ is the target function.

Suppose we have measurements of $\boldsymbol{Z} = \{\boldsymbol{z}_1, \boldsymbol{z}_2, ..., \boldsymbol{z}_t, \boldsymbol{z}_{t+1}, ..., \boldsymbol{z}_T\}$ with time gap Δt, and we wish to predict $\boldsymbol{z}_{t+1}, \boldsymbol{z}_{t+2}, \ldots, \boldsymbol{z}_T$.

We first define the SINDy-class functional based on a library of functions $\Theta(\cdot)$. Mathematically, $\mathcal{F}_{\text{SINDy}}$ defines as:

$$\mathcal{F}_{\text{SINDy}} := \{\Theta\xi : \xi \in \mathbb{R}^p\}, \tag{3}$$

where all $f \in \mathcal{F}_{\text{SINDy}}$ are functions of a convex (linear) combination of functions in $\Theta(\cdot)$, and all functions in the library are within $\mathcal{L}^2(P)$. The space $\mathcal{L}^2(P)$ refers to the set of square-integrable functions with respect to the probability measure P. An example of $\Theta(z)$ is $[z, z^2, z^3, z^4, \sin(z), \cos(z)]$, and this could represent a dynamical system with the following form

$$\dot{z}_t = a_1 z + a_2 z^2 + a_3 z^3 + a_4 z^4 + a_5 \sin(z) + a_6 \cos(z), \tag{4}$$

where $a_1, a_2, ..., a_6$ are constants. Therefore, the feature matrix $\Theta(\boldsymbol{Z}) \in \mathbb{R}^{n\times p}$.

We note that $\mathcal{F}_{\text{SINDy}}$ is a wide class of functions. Since $\Theta(\cdot)$ covers polynomials and the Fourier series, the functional class $\mathcal{F}_0$ could model the governing effect for all differentiable functions from Taylor's approximation.

Then, we consider the neural network way of learning dynamical systems. Unlike SINDy learning, the neural network collects discrete samples $\boldsymbol{z}_{t-L}, \boldsymbol{z}_2, \ldots, \boldsymbol{z}_t$, and to predict $\boldsymbol{z}_{t+1}$. We consider the k-layer ReLU functional class $\mathcal{F}_{\text{ReLU}}$ to be

$$\mathcal{F}_{\text{ReLU}} := \{\boldsymbol{z} \mapsto \sigma_k\left(\boldsymbol{W}_k \sigma_{k-1}\left(\boldsymbol{W}_{k-1} \cdots \sigma_1\left(\boldsymbol{W}_1 \boldsymbol{z}\right)\right)\right)\}, \tag{5}$$

where $\theta = (\boldsymbol{W}_1, \ldots, \boldsymbol{W}_k)$ with $W_1 \in \mathbb{R}^{p\times d}$, $\boldsymbol{W}_i \in \mathbb{R}^{d\times d}$, $\boldsymbol{W}_k \in \mathbb{R}^{d\times p}$, and $\sigma_i(x) = \max(0, \boldsymbol{z})$ represents the ReLU function. Instead of modeling the temporal derivatives, this training strategy believes that the following dynamics are strongly tied with the history samples, and autoregressively predicts the future dynamics. This is commonly used in neural networks training for dynamical systems predictions.

For both cases, we define the error of dynamical system simulation as

$$\mathcal{E}(\boldsymbol{z}, t) = \boldsymbol{z}(t) - \hat{\boldsymbol{z}}(t). \tag{6}$$

Stability It is important to note that, in the following analysis, we set aside stability issues due to the lack of direct theoretical tools. In practice, we observe that the dynamical systems approximated by neural networks are often exhibit instability. Longer rollouts of a learned dynamical system typically lead to poor performance. This is also reflected in the theory that it is hard to obtain guarantees without both a bounded input domain and bounded target values. There is no clear assurance on how neural networks might perform when getting out of the training input domain (1). To facilitate the current analysis, we assume a stable forward simulation scheme by always projecting the $\hat{\boldsymbol{z}}$ back into the input domain. Fully addressing the stability issue for neural networks will require more extensive study in future work.

The error of SINDy-class. In SINDy setting, the optimal functional $\theta^*(\cdot) \in \mathcal{F}_{\text{SINDy}}$ can be parameterized from the following:

$$\theta^*(\boldsymbol{z}) = \Theta(\boldsymbol{z})\xi^*, \tag{7}$$

where ξ^* is a vector in $\mathbb{R}^p$. The functional learning problem shrinks down to a linear parameter estimation problem, and the empirical risk minimizer is the least-square solution that

$$\hat{\xi} = \left(\Theta(\boldsymbol{Z})^T \Theta(\boldsymbol{Z})\right)^{-1} \Theta(\boldsymbol{Z})^\top \hat{\dot{\boldsymbol{Z}}}_t. \tag{8}$$

Here, we estimate the real $\dot{\boldsymbol{z}}_t$ from the data using numerical differentiation methods via $\hat{\dot{z}}_t$. We assume a linear model for $\hat{\dot{z}}_t$ that

$$\hat{\dot{\boldsymbol{Z}}}_t = \Theta(\boldsymbol{Z})\xi^* + \epsilon, \tag{9}$$

where $\epsilon \sim P$ and P is a noise distribution.

Assumption 1 (Lowest eigenvalue). The lowest eigenvalue of $\Theta(\boldsymbol{Z})^\top\Theta(\boldsymbol{Z})$ is bounded from below by cn, where $c > 0$, and n is the sample size.

Assumption 2 (Gaussian white noise). The noise vectors $\{\epsilon_i\}_{i=1}^n$ are independent and

$$\epsilon_i \sim \mathcal{N}(0, s^2 I).$$

Assumption 3 (Regularity SINDy function library). There exists a compact set $\mathcal{K} \subset \mathbb{R}^d$ such that both trajectories $\{z(t), \hat{z}(t)\}_{t\in[0,T]}$ remain in $\mathcal{K}$. Moreover, the true vector field $f^*(z) := \Theta(z)\xi^*$ is L-Lipschitz on $\mathcal{K}$:

$$\|f^*(z) - f^*(z')\|_2 \leq L\|z - z'\|_2, \qquad \forall z, z' \in \mathcal{K},$$

and the library is uniformly bounded on $\mathcal{K}$:

$$R := \sup_{z\in\mathcal{K}} \|\Theta(z)\|_{\text{op}} < \infty.$$

Assumption 1 is an identifiability requirement on the design matrix, which prevents nearly collinear libraries and ensures least-squares will have effective information. Assumption 2 models random error from sensor collection as independent isotropic Gaussian. Assumption 3 is a finite-time stability condition. For example, the true system cannot exhibit finite-time blow-up, and, additionally, the learned rollout must be smooth, and remain in a bounded region over the time horizon. This guarantees that the chosen library functions are reasonable, and the model class is not severely misspecified.

Theorem 1. *Suppose we have a SINDy-class functional with a library of functions $\Theta(z)$, and we estimate the coefficient vector ξ by $\hat{\xi}$ using least squares. Let ξ^* be the true coefficient vector. Then, under suitable regularity conditions on $\Theta(z)$ and the noise in the data, we have*

$$E_\epsilon||\hat{\xi} - \xi^*||_2 = \mathcal{O}\left(s\sqrt{\frac{p}{n}}\right) \qquad [10]$$

where C is a constant depending on the properties of $\Theta(z)$, s is the standard deviation of the noise, p is the number of functions in the library $\Theta(z)$, and n is the number of data points.

Given Assumption 1 and Assumption 2, we have the error in predicting the dynamical system after time T is bounded by

$$E_\epsilon||\hat{z}(T) - z(T)||_2 \leq \mathcal{O}\left(e^{LT}E||\hat{\xi} - \xi^*||\right) = \mathcal{O}\left(e^{LT}s\sqrt{\frac{p}{n}}\right), \qquad [11]$$

where L is the local Lipschitz constant of the system within the region traversed by the trajectories.

Furthermore, with probability $1 - \delta$, the error in predicting the dynamical system after time T is bounded by

$$||\hat{x}(T) - x(T)||_2 \leq \mathcal{O}\left(e^{LT}s\sqrt{\frac{p}{n}\log\left(\frac{1}{\delta}\right)}\right). \qquad [12]$$

Proof.

$$\begin{aligned}
\mathbb{E}_\epsilon\left[\left\|\hat{\xi} - \xi^*\right\|_2^2\right] &= \mathbb{E}_\epsilon\left[\left\|\left(\Theta(\boldsymbol{Z})^T\Theta(\boldsymbol{Z})\right)^{-1}\Theta(\boldsymbol{Z})^\top\hat{\boldsymbol{z}}_t - \xi^*\right\|_2^2\right] & [13]\\
&= \mathbb{E}_\epsilon\left[\left\|\left(\Theta(\boldsymbol{Z})^T\Theta(\boldsymbol{Z})\right)^{-1}\Theta(\boldsymbol{Z})^\top(\Theta(\boldsymbol{Z})\xi^* + \epsilon) - \xi^*\right\|_2^2\right] & [14]\\
&= \mathbb{E}_\epsilon\left[\left\|\left(\Theta(\boldsymbol{Z})^T\Theta(\boldsymbol{Z})\right)^{-1}\Theta(\boldsymbol{Z})^\top\epsilon\right\|_2^2\right] & [15]\\
&= \mathbb{E}_\epsilon\left[\text{Tr}\left[\left(\Theta(\boldsymbol{Z})^T\Theta(\boldsymbol{Z})\right)^{-1}\Theta(\boldsymbol{Z})^\top\epsilon\epsilon^\top\Theta(\boldsymbol{Z})\left(\Theta(\boldsymbol{Z})^T\Theta(\boldsymbol{Z})\right)^{-1}\right]\right] & [16]\\
&= \text{Tr}\left[\left(\Theta(\boldsymbol{Z})^T\Theta(\boldsymbol{Z})\right)^{-1}\Theta(\boldsymbol{Z})^\top\mathbb{E}_\epsilon\left[\epsilon\epsilon^\top\right]\Theta(\boldsymbol{Z})\left(\Theta(\boldsymbol{Z})^T\Theta(\boldsymbol{Z})\right)^{-1}\right] & [17]\\
\text{from Assumption 2} \quad &= s^2\text{Tr}\left[\left(\Theta(\boldsymbol{Z})^T\Theta(\boldsymbol{Z})\right)^{-1}\Theta(\boldsymbol{Z})^\top\Theta(\boldsymbol{Z})\left(\Theta(\boldsymbol{Z})^T\Theta(\boldsymbol{Z})\right)^{-1}\right] & [18]\\
&= s^2\text{Tr}\left[\left(\Theta(\boldsymbol{Z})^T\Theta(\boldsymbol{Z})\right)^{-1}\right] & [19]
\end{aligned}$$

Under Assumption 1 on $\Theta(z)$, the smallest eigenvalues of $\Theta(\boldsymbol{Z})^T\Theta(\boldsymbol{Z})$ is lower bounded by cn. Then the largest eigenvalue of $\left(\Theta(\boldsymbol{Z})^T\Theta(\boldsymbol{Z})\right)^{-1}$ is bounded above by $\frac{1}{cn}$. Therefore,

$$\text{Tr}\left[\left(\Theta(\boldsymbol{Z})^T\Theta(\boldsymbol{Z})\right)^{-1}\right] \leq \frac{p}{cn} \qquad [20]$$

where p is the number of functions in $\Theta(z)$. Therefore,

$$\mathbb{E}_\epsilon\left[\left\|\hat{\xi} - \xi^*\right\|_2^2\right] \leq \frac{s^2p}{cn} \qquad [21]$$

Taking the square root, we have

$$\mathbb{E}_\epsilon \left[\left\| \hat{\xi} - \xi^* \right\|_2 \right] \leq \frac{s\sqrt{p}}{\sqrt{cn}}. \quad [22]$$

The error is $\mathcal{E}(z,t) = z(t) - \hat{z}(t)$. Taking the derivative with respect to t, we get

$$\begin{aligned} \dot{\mathcal{E}}(t) &= \dot{z}(t) - \dot{\hat{z}}(t) && [23] \\ &= \Theta(z(t))\xi^* - \Theta(\hat{z}(t))\hat{\xi} && [24] \\ &= \underbrace{\left(\Theta(z(t))\xi^* - \Theta(\hat{z}(t))\xi^*\right)}_{= f^*(z(t)) - f^*(\hat{z}(t))} + \underbrace{\Theta(\hat{z}(t))(\xi^* - \hat{\xi})}_{\text{parameter error}}. && [25] \end{aligned}$$

By Assumption 3,

$$\|\dot{\mathcal{E}}(t)\|_2 \leq L\|\mathcal{E}(t)\|_2 + B\|\hat{\xi} - \xi^*\|_2. \quad [26]$$

Applying Grönwall's inequality yields, for all $T \in [0, T]$,

$$\begin{aligned} \|\mathcal{E}(T)\|_2 &\leq e^{LT}\|\mathcal{E}(0)\|_2 + \int_0^T e^{L(T-t)} R \|\hat{\xi} - \xi^*\|_2 \, dt && [27] \\ &= e^{LT}\|\mathcal{E}(0)\|_2 + \frac{R}{L}(e^{LT} - 1)\|\hat{\xi} - \xi^*\|_2 && [28] \end{aligned}$$

where the last line absorbs $(e^{LT} - 1)/L \leq e^{LT}$ into constants.
If $\mathcal{E}(0) = 0$, then

$$\|\hat{z}(T) - z(T)\|_2 = \frac{R}{L}(e^{LT} - 1)\|\hat{\xi} - \xi^*\|_2. \quad [29]$$

Therefore,

$$E_\epsilon ||\hat{z}(T) - z(T)||_2 \leq \mathcal{O}\left(e^{LT} E||\hat{\xi} - \xi^*||_2\right) = \mathcal{O}\left(e^{LT} s\sqrt{\frac{p}{n}}\right), \quad [30]$$

from Eqn. 22 and R is a constant that does not scale with n, p, s, L and T.

To get a high-probability bound, we wish to find a bound on $|| \left(\Theta(\boldsymbol{Z})^\top \Theta(\boldsymbol{Z})\right)^{-1} \Theta(\boldsymbol{Z})^\top \epsilon||_2$. Notice from Assumption 2 that this is a sub-Gaussian random vector. Therefore, for any $\delta > 0$, with probability $1 - \delta$, we have

$$\left\| \left(\Theta(\boldsymbol{Z})^\top \Theta(\boldsymbol{Z})\right)^{-1} \Theta(\boldsymbol{Z})^\top \epsilon \right\|_2 \leq \sqrt{2s^2 p \lambda_{\max}((\Theta(\boldsymbol{Z})^\top \Theta(\boldsymbol{Z}))^{-1}) \log(1/\delta)} \quad [31]$$

From Assumption 1, the smallest eigenvalue of $\Theta(\boldsymbol{Z})^T\Theta(\boldsymbol{Z})$ is bounded below by cn. Therefore, the largest eigenvalue of $\left(\Theta(\boldsymbol{Z})^T\Theta(\boldsymbol{Z})\right)^{-1}$ is bounded above by $\frac{1}{cn}$.

Substituting this into the bound, we get that with probability at least $1 - \delta$,

$$||\hat{\xi} - \xi^*||_2 \leq \sqrt{\frac{2s^2 p}{cn} \log(1/\delta)} = s\sqrt{\frac{2p\log(1/\delta)}{cn}}.$$

Therefore, we have that with probability at least $1 - \delta$,

$$\|\hat{z}(T) - z(T)\|_2 \leq \mathcal{O}\left(e^{LT} s \sqrt{\frac{p \log\left(\frac{1}{\delta}\right)}{n}}\right). \quad [32]$$

□

Although the error bound grows exponentially with e^{LT}, empirical results from SINDy modeling show that the error grows almost linearly within finite-time regime. This behavior can be consistent with the exponential error bound above as L is small for a correctly specified model. The normalized input and smooth basis functions that will make $\Theta(\boldsymbol{z})$ and their derivatives tend to vary smoothly and remain small in magnitude within the input domain. Effectively, $\Theta(\boldsymbol{z})$ will have a very small local Lipschitz constant. Consequently, we frequently observe the error will behave linearly on the order of $\mathcal{O}\left(LTs\sqrt{\frac{p}{n}}\right)$ in finite-time. However, over sufficiently long time horizons, exponential divergence will dominate and is unavoidable, particularly for chaotic systems.

The error of neural networks. In a feed-forward network setting, the optimal functional $\theta^*(\cdot) \in \mathcal{F}_{\text{ReLU}}$ can be parameterized from the following:

$$\theta^*(\boldsymbol{z}) = \sigma_k\left(\boldsymbol{W}_L^*\sigma_{k-1}\left(\boldsymbol{W}_{k-1}^* \cdots \sigma_1\left(\boldsymbol{W}_1^*\boldsymbol{z}\right)\right)\right). \qquad [33]$$

This functional learning problem shrinks down to a parametric learning problem as well. The empirical rish minimizer is defined as

$$\hat{\theta} = \arg\min_{\theta} \frac{1}{n}\sum_{i=1}^{n} \ell(f(\boldsymbol{z}_i), \theta(\boldsymbol{z}_i)), \qquad [34]$$

where $\ell(\cdot)$ is the loss function, and $f(\boldsymbol{z}_i)$ is the target value.

Apart from the SINDy-class, the empirical risk minimizer has to be solved via numerical optimization procedures. Additionally, as the loss landscape is non-convex, two-layer ReLU networks are prone to overfitting, and could perform poorly in extrapolation. Fig. S1 shows how the extrapolation of neural networks can be suboptimal for learning dynamical systems. In the following, we present the error analysis for neural networks in dynamical system learning.

We require the following assumptions for theoretical analysis on neural networks.

Assumption 4 (True dynamics). There exists a compact set $\mathcal{K} \subset \mathbb{R}^d$ such that both trajectories $\{z(t), \hat{z}(t)\}_{t\in[0,T]}$ remain in $\mathcal{K}$. Moreover, the true vector field $f^* : \mathcal{K} \to \mathbb{R}^d$ is L-Lipschitz on $\mathcal{K}$:

$$\|f^*(z) - f^*(z')\|_2 \leq L\|z - z'\|_2, \qquad \forall z, z' \in \mathcal{K}.$$

Finally, the state is uniformly bounded on $\mathcal{K}$:

$$R := \sup_{z\in\mathcal{K}} \|z\|_2 < \infty.$$

Assumption 5 (Neural network model class). Consider the class $\mathcal{F}_{\text{ReLU}}$ of k-layer ReLU networks $f_\theta(\cdot)$ with parameters $\theta = (W_1, \ldots, W_k)$. Assume the weights are uniformly bounded:

$$B := \sup_{f_\theta\in\mathcal{F}_{\text{ReLU}}} \max_{\ell\in\{1,\ldots,k\}} \|W_\ell\|_F < \infty.$$

Moreover, the induced one-step predictor is stable on $\mathcal{K}$ in the sense that

$$f_\theta(\mathcal{K}) \subseteq \mathcal{K}, \qquad \forall f_\theta \in \mathcal{F}_{\text{ReLU}}.$$

Assumption 6 (Bounded loss). The training data consist of i.i.d. pairs $\{(z_0^{(i)}, z_H^{(i)})\}_{i=1}^n$ supported on $\mathcal{K}\times\mathcal{K}$, where $H = T/\Delta t$. Define the H-step rollout predictor

$$f_\theta^{(H)} := \underbrace{f_\theta \circ \cdots \circ f_\theta}_{H \text{ times}},$$

and the normalized squared loss

$$\ell\big(f_\theta^{(H)}(z_0), z_H\big) := \frac{1}{4R^2}\|f_\theta^{(H)}(z_0) - z_H\|_2^2.$$

Under Assumption 4, we have $\ell \in [0, 1]$.

Assumption 4 is similar to Theorem 1 which describes the statistical model. Assumption 5 is a technical assumption that ensures the neural network weights are bounded to avoid unexpected extrapolation. Assumption 6 is used to simplify the constant bound for Big-O analysis.

Theorem 2. *Suppose we have a neural network functional with k layers of ReLU activation functions and parameters $\theta = (\boldsymbol{W}_1, \ldots, \boldsymbol{W}_k)$, which computes functions*

$$f(\boldsymbol{z}; \theta) = \sigma_k\left(\boldsymbol{W}_k\sigma_{k-1}\left(\boldsymbol{W}_{k-1} \cdots \sigma_1\left(\boldsymbol{W}_1\boldsymbol{z}\right)\right)\right). \qquad [35]$$

Suppose we have Assumption 4-7. Let $\hat{f}$ denote the empirical risk minimizer that achieves zero empirical loss and define the H-step rollout $\hat{z}(T) = \hat{f}^{(H)}(z_0)$ and $z(T) = z_H$. Then, with probability at least $1-\delta$, the prediction error after $H = T/\Delta t$ steps satisfies

$$\mathbb{E}\left[\|\hat{z}(T) - z(T)\|_2^2\right] \leq \mathcal{O}\left((\log n)^4 B^{k(H+1)}\sqrt{\frac{k}{n}} + \frac{\log(1/\delta)}{n}\right). \qquad [36]$$

Proof. From the normalized squared loss from Assumption 7:

$$\ell\big(f^{(H)}(z_0), z_H\big) := \frac{1}{4R^2}\|f^{(H)}(z_0) - z_H\|_2^2 \in [0,1]. \tag{37}$$

Let $L(f)$ and $\hat{L}(f)$ denote the population and empirical risks for this loss:

$$L(f) := \mathbb{E}\,\ell\big(f^{(H)}(Z_0), Z_H\big), \qquad \hat{L}(f) := \frac{1}{n}\sum_{i=1}^{n}\ell\big(f^{(H)}(z_0^{(i)}), z_H^{(i)}\big). \tag{38}$$

From Lemma 2, with probability at least $1-\delta$, we have

$$L(f) \leq (1+c)\hat{L}(f) + c(\log n)^4 \mathcal{R}_n(\mathcal{F}_{\mathrm{ReLU}}^{(H)}) + \frac{c\log(1/\delta)}{n}, \tag{39}$$

where $c > 0$ is a universal constant.

We aim to control the generalization error after $H = T/\Delta t$ steps. From Lemma 5, the Rademacher complexity after H compositions is bounded by

$$\mathcal{R}_n(\mathcal{F}_{\mathrm{ReLU}} \circ \cdots \circ \mathcal{F}_{\mathrm{ReLU}}) \lesssim B^{kH}\frac{\sqrt{k}B^k}{\sqrt{n}} = B^{k(H+1)}\sqrt{\frac{k}{n}}. \tag{40}$$

Substituting this bound into Lemma 2 yields, with probability at least $1-\delta$,

$$L(f) \leq (1+c)\hat{L}(f) + c(\log n)^4 B^{k(H+1)}\sqrt{\frac{k}{n}} + \frac{c\log(1/\delta)}{n}. \tag{41}$$

Finally, by the definition of ℓ we have

$$L(f) = \mathbb{E}\left[\frac{1}{4R^2}\|f^{(H)}(Z_0) - Z_H\|_2^2\right], \tag{42}$$

and therefore, multiplying both sides by $4R^2$ (and absorbing constants into $\mathcal{O}(\cdot)$), we obtain the desired bound

$$\mathbb{E}\left[\hat{z}(T) - z(T)\|_2^2\right] \leq \mathcal{O}\left((\log n)^4 B^{k(H+1)}\sqrt{\frac{k}{n}} + \frac{\log(1/\delta)}{n}\right). \tag{43}$$

□

Technical Lemmas for the Proof of Thm. 2. We introduce the following lemma from (2) and rewrite it within our context.

Lemma 1 (Thm. 2.11 in (2))**.** *Let $\sigma : \mathbb{R} \to \mathbb{R}$ be the ReLU activation function. Consider a feed-forward network with k layers of these nonlinearities and parameters $\theta = (\boldsymbol{W}_1, \ldots, \boldsymbol{W}_L)$, which computes functions*

$$f(\boldsymbol{z};\theta) = \sigma_k\left(\boldsymbol{W}_k\sigma_{k-1}\left(\boldsymbol{W}_{k-1}\cdots\sigma_1\left(\boldsymbol{W}_1\boldsymbol{z}\right)\right)\right). \tag{44}$$

Define the class of functions on the unit Euclidean ball in $\mathbb{R}^p$,

$$\mathcal{F}_B = \left\{f(\cdot;\theta) : \|\boldsymbol{W}_i\|_F \leq B\right\}, \tag{45}$$

where $\|\boldsymbol{W}_i\|_F$ is the Frobenius norm of $\boldsymbol{W}_i$. Then, we have the Rademacher complexity of $\mathcal{F}$ is bounded by

$$\mathcal{R}_n(\mathcal{F}_B) \lesssim \frac{\sqrt{k}B^k}{\sqrt{n}}. \tag{46}$$

This result is from (3) and (2). We then introduce the following connection between the Rademacher complexity and the generalization error.

Lemma 2 (Thm. 2.2 in (2))**.** *Let $\mathcal{F}_B$ be a class of neural networks with weights bounded by B, and let $f \in \mathcal{F}_B$. For the mean-squared loss that $\ell(\hat{y}, y) = (\hat{y} - y)^2$, and for any distribution on $\mathcal{X} \times [-1, 1]$ where the output is bounded. With probability at least $1-\delta$, there exists a constant $c > 0$ such that $\forall f \in \mathcal{F}_B$:*

$$L(f) \leq (1+c)\hat{L}(f) + c(\log n)^4\mathcal{R}_n(\mathcal{F}_B) + \frac{c\log(1/\delta)}{n}, \tag{47}$$

where $L(f)$ is the true risk, $\hat{L}(f)$ is the empirical risk, n is the number of data points, and B is a bound on the weights of the neural network.

An important observation here is that, even with a fixed set of parameters, for autoregressive neural networks, the rademacher complexity grows massively with longer time horizons. In other words, if we want to predict T steps ahead, the network size will grow exponentially with T. We use the following lemma called the contraction principle to analyze the expected network growth in this scenario.

Lemma 3. *Let $\mathcal{F}_1$ be a class of functions from $\mathbb{R}^p$ to $\mathbb{R}^q$ with Lipschitz constant α. Let $\mathcal{F}_2$ be a class of functions from $\mathbb{R}^q$ to $\mathbb{R}^r$. Then, the contraction principle describes that the Rademacher complexity of $\mathcal{F}_1 \circ \mathcal{F}_2$ is*

$$R_n(\mathcal{F}_1 \circ \mathcal{F}_2) \leq \alpha \mathcal{R}_n(\mathcal{F}_2). \qquad [48]$$

The proof of this lemma can be found in (4, 5). From this Lemma, we further control the Lipschitz constant for neural network in the following:

Lemma 4 (Proposition 2 in (6))**.** *Suppose we have a neural network functional with k layers of ReLU activation functions and parameters $\theta = (\boldsymbol{W}_1, \ldots, \boldsymbol{W}_L)$, which computes functions*

$$f(\boldsymbol{z}; \theta) = \sigma_k \left(\boldsymbol{W}_k \sigma_{k-1} \left(\boldsymbol{W}_{k-1} \cdots \sigma_1 \left(\boldsymbol{W}_1 \boldsymbol{z}\right)\right)\right). \qquad [49]$$

The Lipschitz constant of the network is bounded by

$$\prod_{i=1}^{k} \|\boldsymbol{W}_i\|_2 \leq B^k, \qquad [50]$$

where $\|\boldsymbol{W}_i\|_2$ is the spectral norm of $\boldsymbol{W}_i$.

Proof. Since the ReLU activation function has a Lipschitz constant of 1, the Lipschitz constant of the i-th layer is bounded by $\|\boldsymbol{W}_i\|_2$. Therefore, from Lemma 3, for a k-layer neural network with weights $\theta = (\boldsymbol{W}_1, \ldots, \boldsymbol{W}_k)$, the Lipschitz constant of the entire network is bounded by the product of the spectral norms of the weight matrices:

$$\mathrm{Lip}(f(\boldsymbol{z}; \theta)) \leq \prod_{i=1}^{k} \|\boldsymbol{W}_i\|_2. \qquad [51]$$

Given that $\|\boldsymbol{W}_i\|_F \leq B$ for all i, and $\|\boldsymbol{W}_i\|_2 \leq \|\boldsymbol{W}_i\|_F$, we have

$$\prod_{i=1}^{k} \|\boldsymbol{W}_i\|_2 \leq \prod_{i=1}^{k} \|\boldsymbol{W}_i\|_F \leq B^k. \qquad [52]$$

This means that the Lipschitz constant of the neural network is bounded by B^k. □

From the Lemma above, we could obtain the Rademacher complexity of neural network prediction up to $H = \frac{T}{\Delta t}$ steps forward.

Lemma 5. *The Rademacher complexity of the neural network functional class after H forward unroll operations for $\mathcal{F}_{ReLU}$ is bounded by*

$$\mathcal{R}_n(\mathcal{F}_{ReLU} \circ \cdots \circ \mathcal{F}_{ReLU}) \lesssim B^{\frac{kT}{\Delta t}} \frac{\sqrt{k} B^k}{\sqrt{n}}. \qquad [53]$$

Proof. From Lemma 4, we know that the Lipschitz constant of the neural network is bounded by B^k. Therefore, the Rademacher complexity of the neural network functional class is bounded by

$$\mathcal{R}_n(\mathcal{F}_{\mathrm{ReLU}}) \lesssim \frac{\sqrt{k} B^k}{\sqrt{n}}. \qquad [54]$$

From Lemma 3, we know that the Lipschitz constant of the network is bounded by B^k. Therefore, the Rademacher complexity of the neural network functional class after H forward unroll operations is bounded by

$$\mathcal{R}_n(\mathcal{F}_{\mathrm{ReLU}} \circ \cdots \circ \mathcal{F}_{\mathrm{ReLU}}) \lesssim B^{\frac{kT}{\Delta t}} \frac{\sqrt{k} B^k}{\sqrt{n}}. \qquad [55]$$

□

Observation on the convex landscape

We demonstrate our observations on the convexity of the loss landscape of SHRED. To visualize the loss landscape in a high-dimensional space, we utilize a popular method (7) which represents the landscape in the following way. Suppose the neural network is parameterized by θ, which includes the weights and biases of all layers. We perturb θ_0 with two random directions $\boldsymbol{r}_x, \boldsymbol{r}_y$ via

$$\theta' = \theta_0 + t \cdot \alpha \boldsymbol{r}_x + t \cdot \alpha \boldsymbol{r}_y, \quad [56]$$

where $\boldsymbol{r}_x, \boldsymbol{r}_y$ are both normalized i.i.d. Gaussian samples. α is the scale of changes and $t \in [0, 1]$ moves from θ_0 towards the linear combination of the two random directions.

In Fig. S2, we visualize the loss landscape of SHRED from different random directions using the methodology described above under various network depths and scales α. Across all subplots, we consistently observe a clear global minimum with a convex landscape. Furthermore, Fig. S3 presents an empirical convexity analysis, showing loss changes along several paths from different initializations. We evaluate convexity using randomly sampled points and find that all of them satisfy the convexity condition. We use a tolerance of 10^{-7} to counter floating-point imprecision.

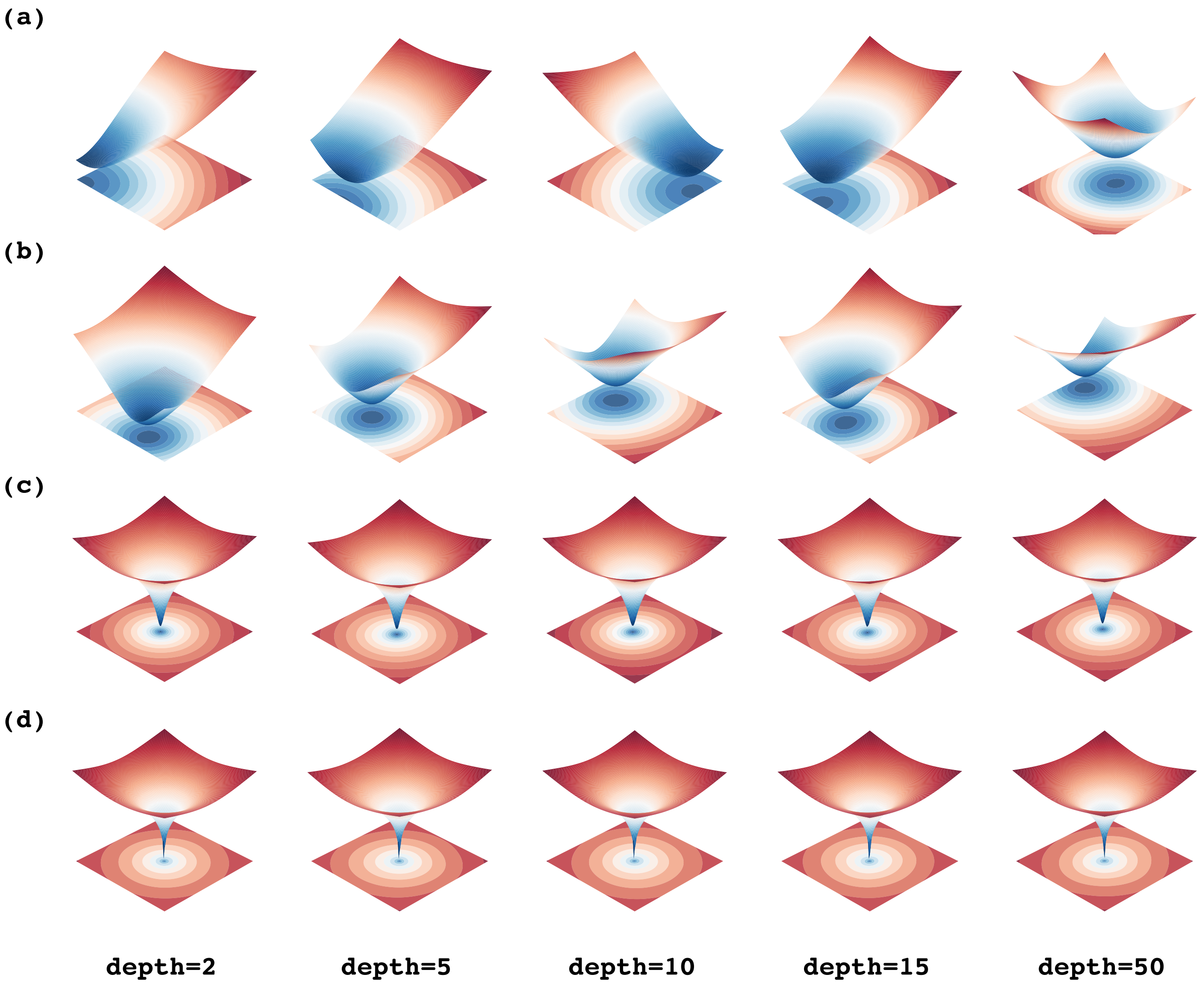

Figure S2. (a) Visualization of loss landscape from parameters ranging from $[-5, 5]$. (b) Visualization of loss landscape from parameters ranging from $[-10, 10]$. (c) Visualization of loss landscape from parameters ranging from $[-100, 100]$. (d) Visualization of loss landscape from parameters ranging from $[-1000, 1000]$.

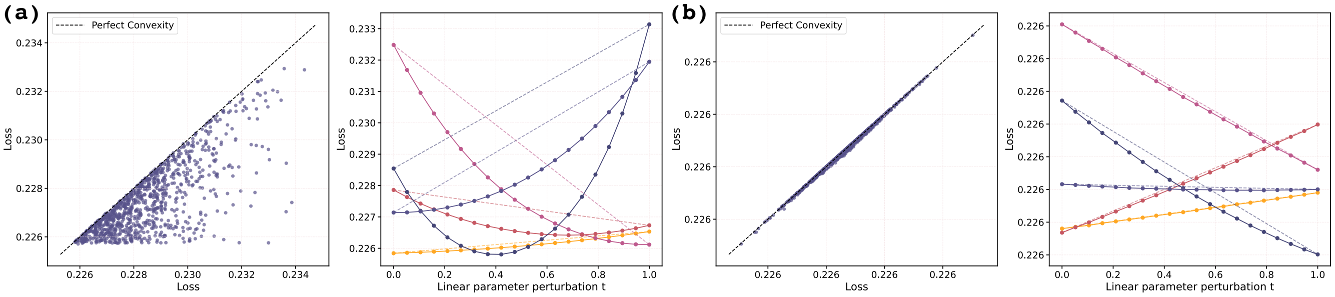

Figure S3. Visualization of loss landscapes for SHRED parameters along random directions, showing how loss values change when moving from the origin toward randomly generated points in parameter space. The figure provides a detailed view of loss behavior at different initial points. **(a)** shows the parameter range $[-100, 100]$ and **(b)** shows the parameter range $[-1, 1]$.

Ablation study

Effect on the recurrent units. In this study, we investigate the effect of different recurrent unit choices on the ability of SINDy-SHRED to learn parsimonious latent dynamics. Although in theory many recurrent architectures could be effective, subtle differences in their modeling strategies could induce different latent spaces.

Recurrent neural network (RNN). The Elman network (8) (often referred to generically as RNN) has the following mathematical formulation,

$$z_t = \tanh\left(x_t W_{iz}^\top + b_{iz} + z_{t-1} W_{zz}^\top + b_{zz}\right), \qquad [57]$$

where z_t is the hidden state at time t, x_t is the input at time t, and h_{t-1} is the hidden state at time $t-1$. The initial hidden state z_0 is set to be 0.

Long short-term memory (LSTM) network. The LSTM network is a recurrent unit that utilizes gating mechanisms to help ameliorate issues with vanishing and exploding gradients that often arise in the training of RNNs. The mathematical formulation of the LSTM is

$$\begin{aligned}
i_t &= \sigma\left(W_{ii} x_t + b_{ii} + W_{zi} z_{t-1} + b_{zi}\right), \\
f_t &= \sigma\left(W_{if} x_t + b_{if} + W_{zf} z_{t-1} + b_{zf}\right), \\
o_t &= \sigma\left(W_{io} x_t + b_{io} + W_{zo} z_{t-1} + b_{zo}\right), \\
g_t &= \tanh\left(W_{ig} x_t + b_{ig} + W_{zg} z_{t-1} + b_{hg}\right), \\
c_t &= f_t \odot c_{t-1} + i_t \odot g_t, \\
z_t &= o_t \odot \tanh(c_t),
\end{aligned} \qquad [58]$$

where i_t, f_t, o_t are the input, forget, and output gates respectively. Here, z_t is the hidden state and c_t is the cell state at time t. Both z_0 and c_0 are set to be 0.

Gated recurrent unit (GRU). The GRU network simplifies LSTM network with the following mathematical formulation:

$$\begin{aligned}
r_t &= \sigma\left(W_{ir} x_t + b_{ir} + W_{zr} z_{t-1} + b_{zr}\right), \\
h_t &= \sigma\left(W_{ih} x_t + b_{ih} + W_{zh} z_{t-1} + b_{zh}\right), \\
n_t &= \tanh\left(W_{in} x_t + b_{in} + r_t \odot \left(W_{zn} z_{t-1} + b_{zn}\right)\right), \\
z_t &= (1 - h_t) \odot n_t + h_t \odot z_{t-1},
\end{aligned} \qquad [59]$$

where r_t is the reset gate and h_t is the update gate. Here, z_t is the hidden state at time t, and z_{t-1} represents the hidden state at time $t-1$. The initial hidden state z_0 is set to be 0.

Empirically, we are interested in choosing the network architecture that permits the smoothest representation of latent trajectories. Here, we define smoothness as latent trajectories that have small state to state fluctuations. Among these three architectures, the RNN network tends to have more spikes, as the change from z_{t-1} to z_t is directly tied to a nonlinear $\tanh(\cdot)$ function. Both LSTM and GRU networks introduce extra gates, which ensure that the hidden states will evolve more smoothly. The GRU network is preferred because only a single latent state is propagated, in contrast to the LSTM which propagates a hidden state and a cell state.

In Fig. S4, we visualize the difference between the three recurrent network architectures using the sea-surface temperature dataset. We train all three networks with the same optimizer parameters and latent state dimension and plot their latent space trajectories on the test set. We see that visually, the GRU produces the smoothest latent trajectory. Across all of our computational examples, we found the best performance using GRUs. Nevertheless, it is possible that other architectures could prove superior for certain dynamical systems.

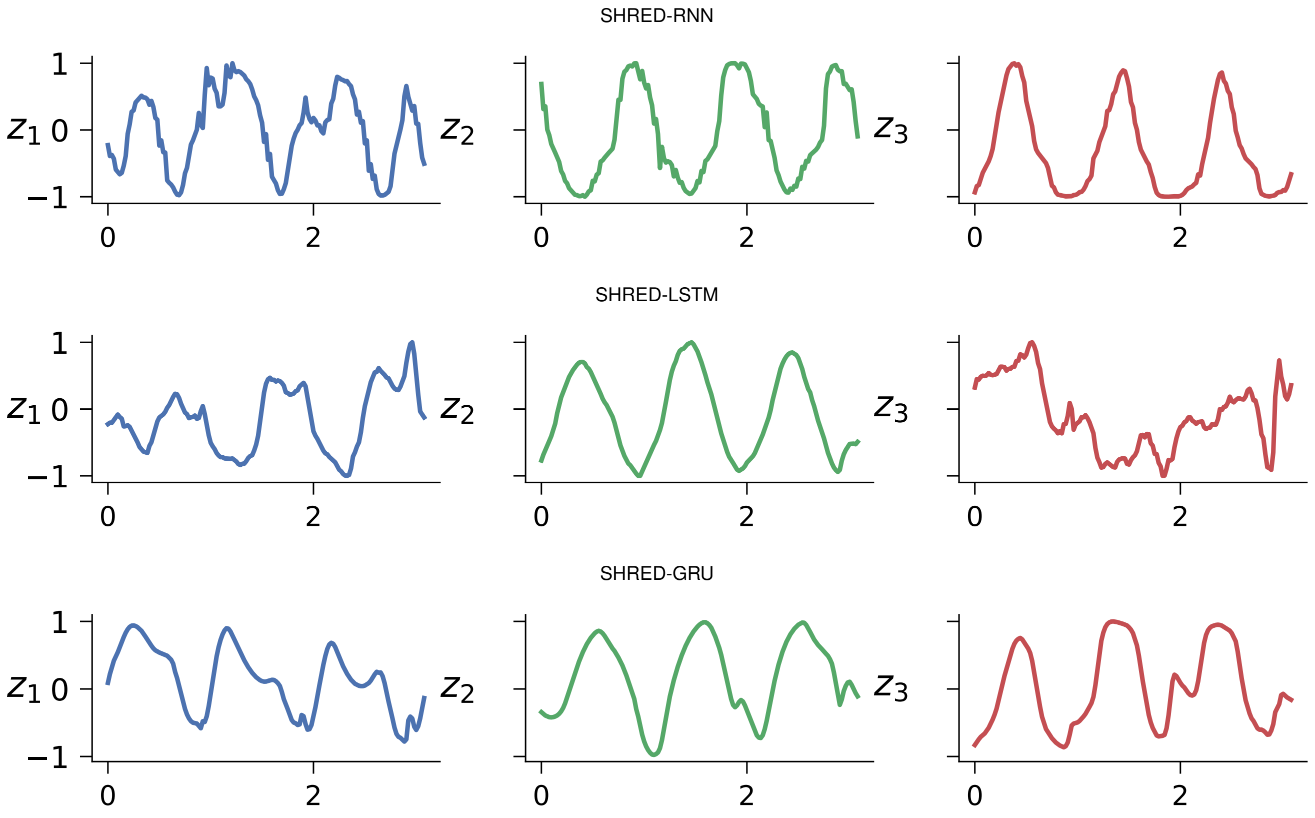

Figure S4. Comparison of latent spaces learned by SHRED (no SINDy regularization) with different recurrent units on the test set. The x-axis represents the number of years in the sea-surface temperature dataset.

	SINDy-SHRED	SINDy	SINDy-AE	CNN-SINDy	SHRED
Model discovery	✓	✓	X	X	X
High dimensionality	✓	X	✓	✓	✓
Interpretable	✓	✓	✓	✓	✓
Short-term prediction	✓	X	✓	✓	✓
Long-term rollouts	✓	X	X	X	X
Reproducibility	✓	✓	X	✓	✓

Table S1. Comparison of different SINDy-based and SHRED-based methods in model discovery, spatiotempora learning, and reproducibility.

A comparison to other baseline methods. In Table S1, SINDy-SHRED is compared to all baseline methods. SINDy algorithm (9) is not designed for high-dimensional problem, which requires a pre-specification of a good coordinate transform. Therefore, it works poorly for both short- and long-term predictions for high-dimensional datasets. With autoencoders, SINDy-autoencoder (10) can work on high-dimensional data. However, because autoencoders impose no constraints on the derived latent space, SINDy-AE generally struggles to find a stable physical system in the latent space that does not diverge during rollout. CNN-SINDy (11) does not jointly solve SINDy and the latent dynamics, which typically results in an unnecessarily complex dynamical system. SHRED (12) is primarily designed as a sensing method, which does not have built-in capability for model discovery and long-term predictions.

Quantitative comparison by training SHRED with and without SINDy loss terms. In what follows, we present a comparison study between SHRED and SINDy-SHRED.

Equation discovery. We visualize the latent spaces of SHRED and SINDy-SHRED in Fig. S5 for the sea-surface temperature dataset. Starting from the same weight initialization, we observe that the derived latent spaces of SHRED and SINDy-SHRED are visually different. For every 50 epochs, we plot the induced latent space by the two models from epoch 50 (light blue) to epoch 1000 (dark green). The latent space of SINDy-SHRED converges to a smoother dynamical system without frequent spiking behavior, demonstrating that SINDy-SHRED does act as a regularizer on the dynamics of the latent space.

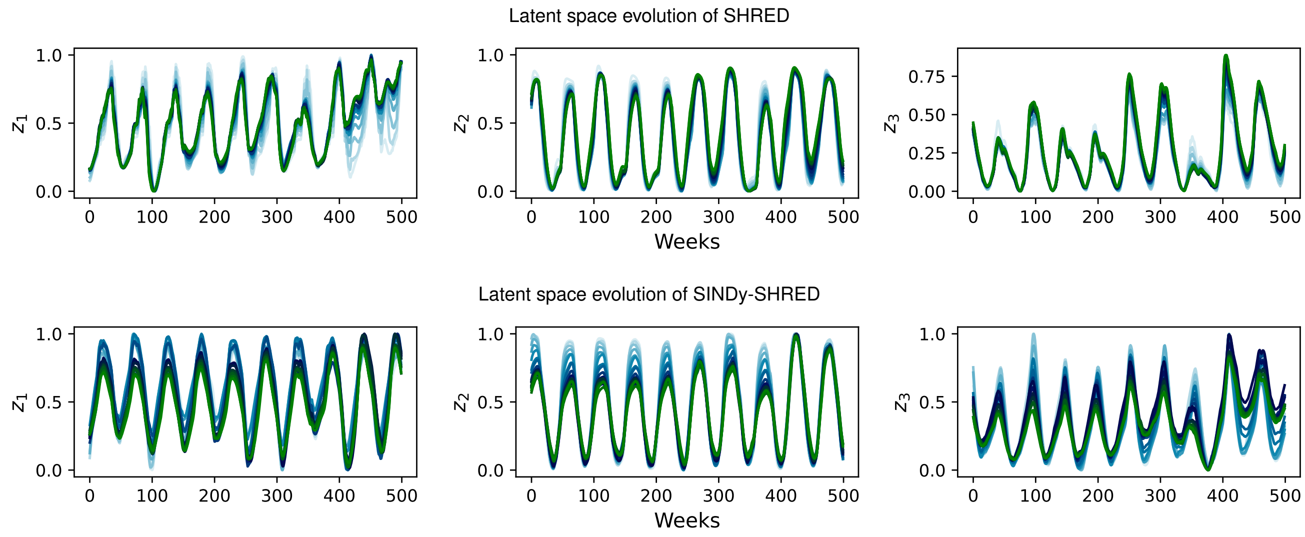

Figure S5. Comparison of latent spaces learned by SHRED (top row) and SINDy-SHRED (top row). The color represents the induced latent space evolution from early time (blue) to the end of training (green).

Additionally, the strength of regularization plays an important role in equation discovery, which is shown in Fig. S6. The latent space trajectories are gradually regularized into an ODE model with an explicit formulation.

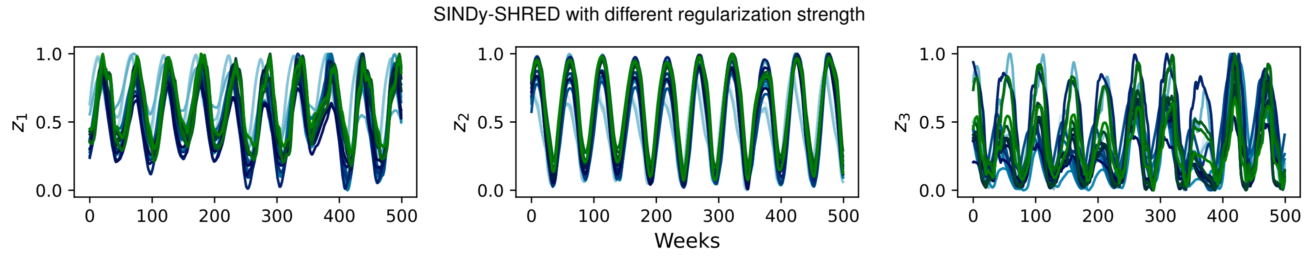

Figure S6. Top row: the latent space evolution with increasingly stronger SINDy regularization parameter, ranging from 0.1 to 10.

In Fig. S7, we visualize the latent space of a SHRED model without SINDy regularization. The result is latent dynamics which fail to be well-described by SINDy model.

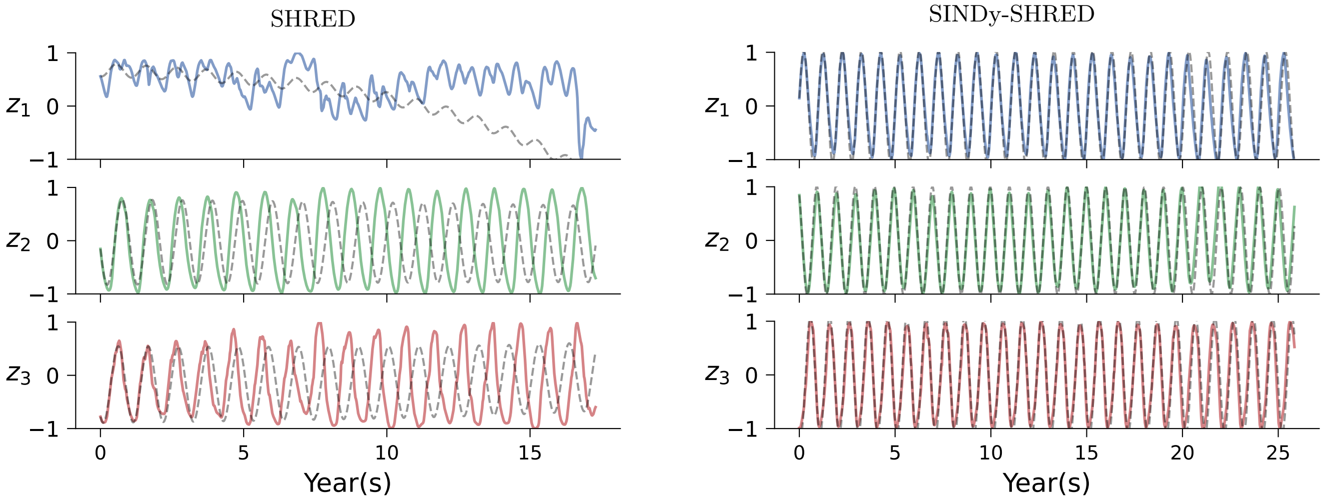

Figure S7. Comparison of the latent space discovery from SHRED and SINDy-SHRED. Without SINDy regularization, SHRED has no guarantee to compress the raw observation into a physically interpretable latent space. SINDy-SHRED finds an accurate ODE model that can be used to achieve long term forecasts.

Predictive power. An additional benefit of the SINDy-SHRED method is increased forecasting poerformance in comparison to SHRED. To enable standard SHRED to perform spatio-temporal forecasting, one strategy mentioned in (12) is to first train a separate GRU network to predict the sparse input sensor measurements. Then, from this trained predictor for the sparse sensors, one can autoregressively forecast the sparse sensor sequence from available data (e.g. in Fig. S8). By treating the predicted sparse sensors as inputs, standard SHRED can produce a prediction of the spatial field.

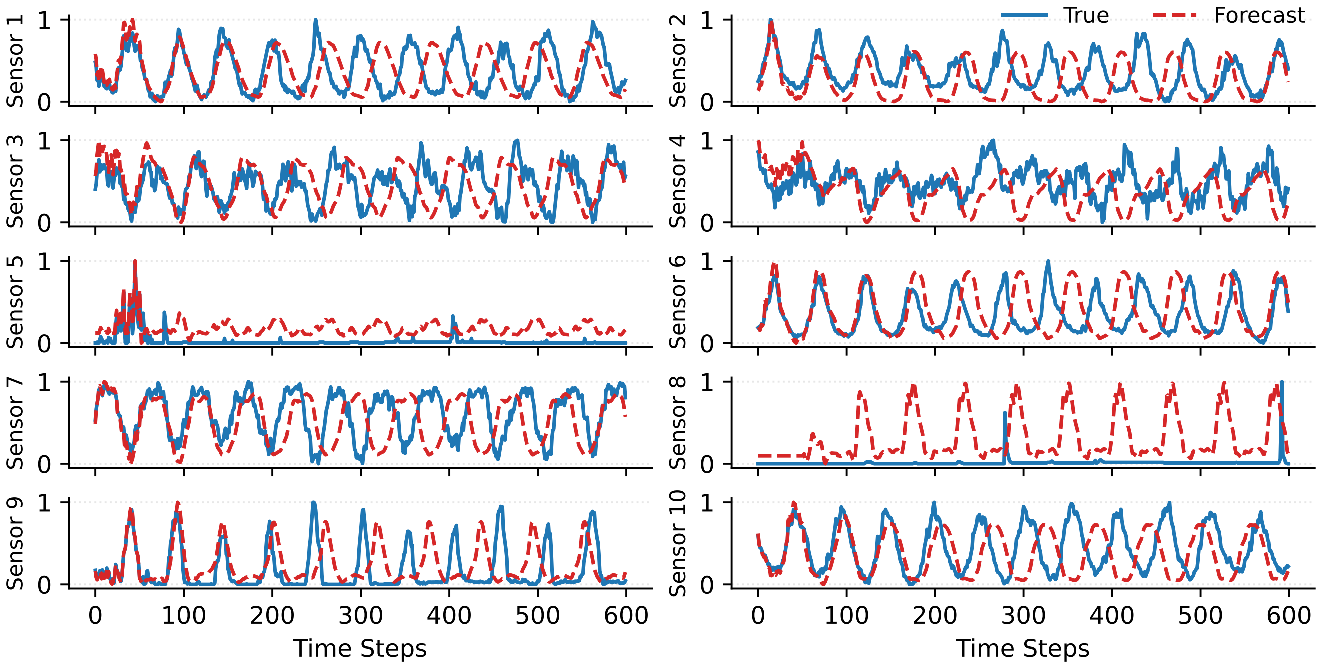

Figure S8. Comparison of long-term prediction between SHRED and SINDy-SHRED. The frame error is evaluated by each frame (left) and the cumulative error is calculated by summing up all previous frame errors (right).

As shown in Fig. S9, we find SINDy-SHRED greatly outperforms standard SHRED in in such predictions. This behavior is in agreement with that inFig. S8, which shows that the long-term rollout prediction from GRU network tends to be inaccurate. Utilizing an explicit ODE model via SINDy-SHRED can stabilize its long-term behavior and reduce the prediction error in general.

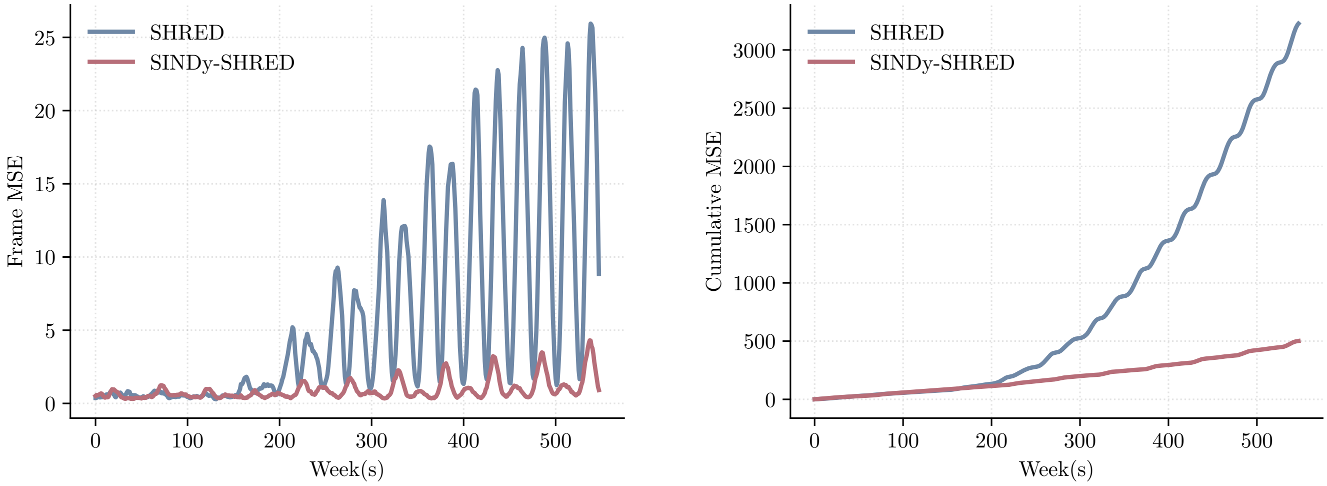

Figure S9. Comparison of long-term prediction between SHRED and SINDy-SHRED. The frame error is evaluated by each frame (left) and the cumulative error is calculated by summing up all previous frame errors (right).

Latent space discoveries from different sensor placements are equivalent under linear transformations. Different sensor placements can lead SINDy-SHRED to recover the same underlying physical dynamics, but expressed in distinct latent coordinate systems related by a linear transformation. This occurs because the governing dynamics are invariant under linear transformations such as rotations and scalings, so the same dynamics may appear with different symbolic representations in different coordinate frames. In practice, varying the sensor placement introduces an implicit prior that biases SINDy-SHRED toward learning the system from a particular geometric perspective.

To illustrate this equivalence, we use sea-surface temperature data as an example. We train SINDy-SHRED with a latent dimension of two (chosen for ease of visualization) while keeping all other training settings fixed. We then compare the raw latent spaces identified from two different sensor configurations to examine how sensor placement affects the learned coordinate representation. As shown in Fig. S10, the raw latent spaces obtained from the two sensor configurations appear different but are equivalent under a linear transformation. We identify this transformation using linear regression to map the latent variables from the first run (latent 1) onto those from the second (latent 2). This result aligns with previous findings in autoencoder-based SINDy (10) (see Supplemental Figure S1), where models trained under different conditions were likewise equivalent up to an affine transformation.

Both discoveries provide equally valid representations of the underlying dynamics. In principle, one could identify the most parsimonious representative within the equivalence class defined by the full group of linear transformations, but this procedure is computationally expensive (13).

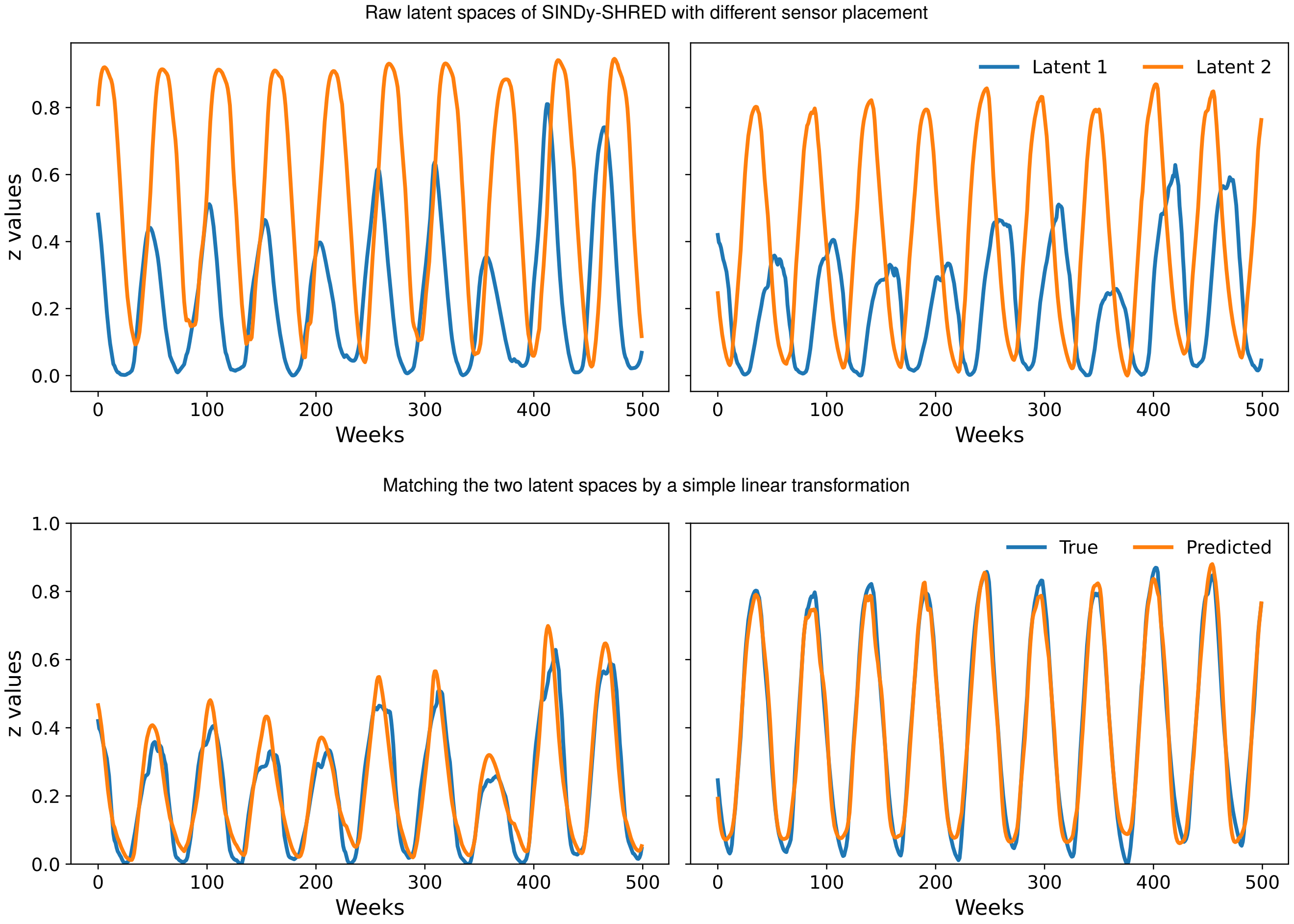

Figure S10. Latent space discovery from the sea-surface temperature data under two different sensor placements. The raw discoveries appear to be different but the two discoveries are close under a linear transformation.

Hyperparameter robustness. The following study supports the empirical observation that the SINDy-SHRED architecture is robust to hyperparameter choices (e.g. learning rate and batch sizes). As shown in Fig. S11, we retrain SINDy-SHRED on the sea-surface temperature dataset using the same set of sensors with different hyperparameter settings. We keep all other settings to be the same in the main SST experiment. Across these ablation trails, we observe that all configurations converge at similar rates and achieve similar final errors.

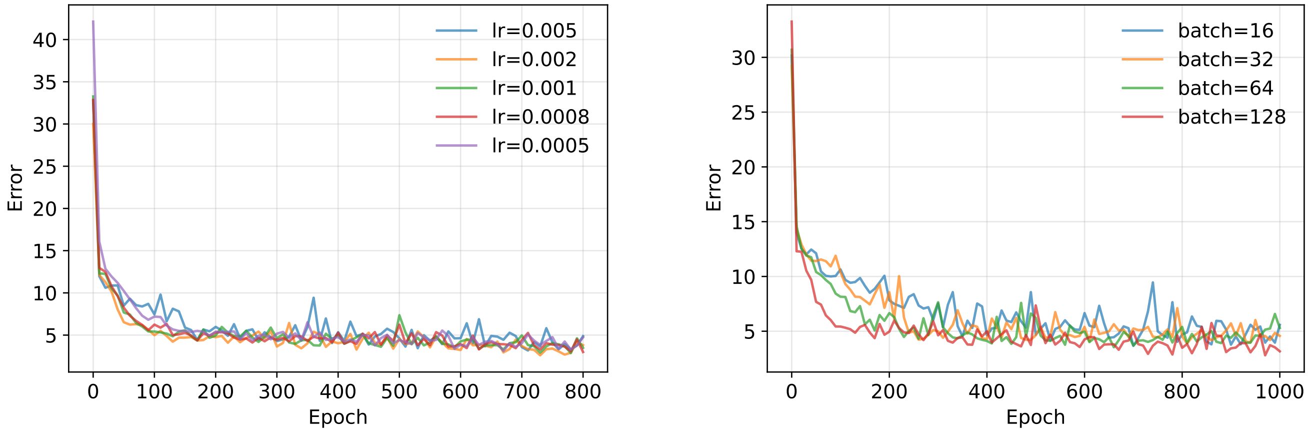

Figure S11. Error decay with training epochs using different hyperparameter settings: learning rates (left) batch sizes (right). All configurations converge to a similar level of training error.

Energy spectrum analysis on the isotropic turbulent flow. Fig. S12 provides a detailed evaluation of the physical quantities predicted by SINDy-SHRED. The energy spectrum is computed on available ground-truth 2D pressure field snapshots and SINDy-SHRED predictions using 2D FFT, summarizing the distribution of energy across spatial scales. SINDy-SHRED effectively preserves the spectral characteristics of the real data. The probability distribution histogram further visualizes the empirical distributions of the predicted and real data. The two distributions largely overlap with each other, showing minor or no distributional shift. We see that the predicted data has a smaller tail than compared to the ground truth, indicating that the learned model smooths out high-frequency content. For reference, in 3D incompressible isotropic turbulence, the pressure spectrum is theoretically expected to scale as $k^{-7/3}$ in the inertial range (14). The presented spectrum is computed from 2D slices which is not directly comparable to the full 3D data ($k^{-7/3}$), but we observe SINDy-SHRED a similar energy spectrum compared to the ground truth.

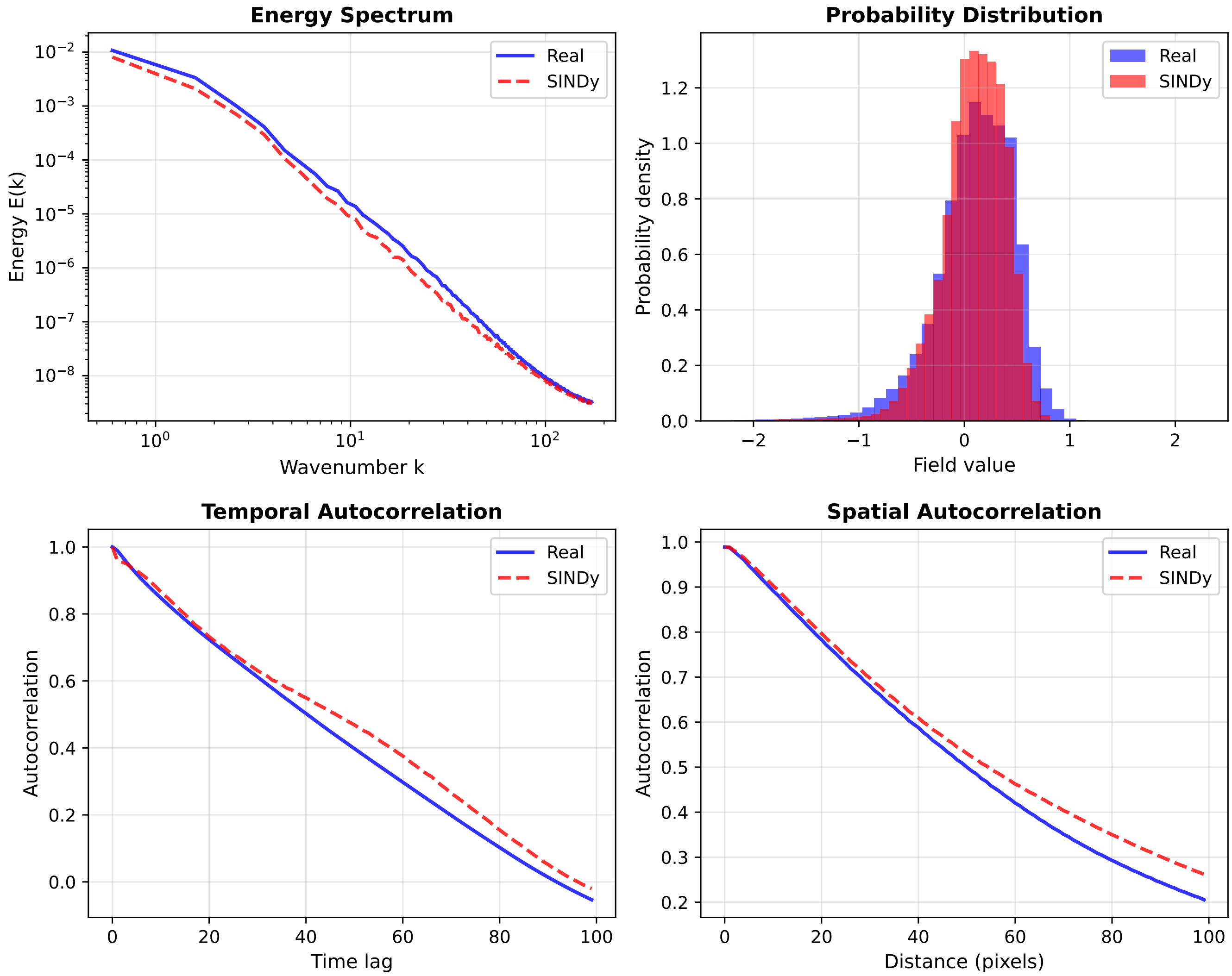

Figure S12. SINDy-SHRED prediction captures important statistical properties for long-term predictions.

Experimental details

The design of SINDy unit. In Fig. S13, we show the design of SINDy unit. Notice how this design is similar to the skip-connection mechanism in ResNet. Here, it models the forward simulation process of ODE integration by having $\boldsymbol{x}_{t+1} = \boldsymbol{x}_i + \Theta(\boldsymbol{x})\Xi\Delta t$.

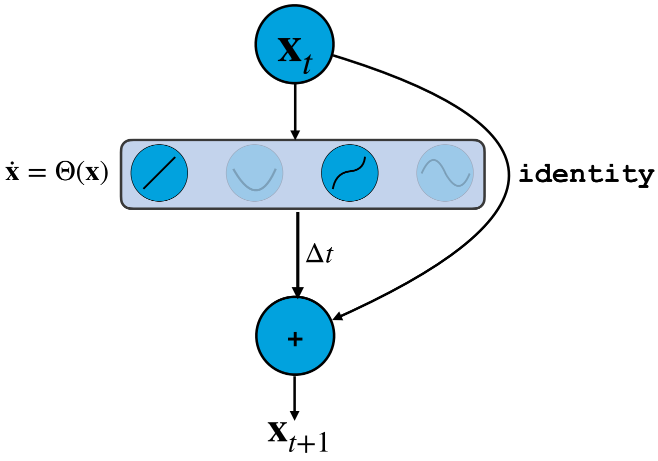

Figure S13. Diagram of the RNN form of SINDy.

Evaluation metrics. We define the following evaluation metrics that are commonly used to assess model's performance.

The **mean squared error** is defined as the L_2 distance between the real target data $\boldsymbol{X}$ to the predicted target $\hat{\boldsymbol{X}}$ that

$$\text{MSE} = \frac{1}{n}\sum_i \left(\boldsymbol{X}_i - \hat{\boldsymbol{X}}_i\right)^2, \qquad [60]$$

where $\boldsymbol{X}$ has n samples.

The **mean absolute error** is defined as the L_1 distance between the real target data $\boldsymbol{X}$ to the predicted target $\hat{\boldsymbol{X}}$ that

$$\text{MAE} = \frac{1}{n}\sum_i |\boldsymbol{X}_i - \hat{\boldsymbol{X}}_i|, \qquad [61]$$

where $\boldsymbol{X}$ has n samples.

Additionally, we define the **relative mean-squared error** for both spatiotemporal data $\boldsymbol{X}$ and latent space trajectory $\boldsymbol{Z}$ that

$$\text{rMSE}(\boldsymbol{X}, \hat{\boldsymbol{X}}) = \frac{\left\|\boldsymbol{X} - \hat{\boldsymbol{X}}\right\|_2^2}{\|\boldsymbol{X}\|_2^2} \qquad [62]$$

$$\text{rMSE}(\boldsymbol{Z}, \hat{\boldsymbol{Z}}) = \frac{\left\|\boldsymbol{Z} - \hat{\boldsymbol{Z}}\right\|_2^2}{\|\boldsymbol{Z}\|_2^2}, \qquad [63]$$

Flow over a cylinder. In the flow over a cylinder experiment, we follow the same settings as in the prior experiments and select the latent dimension to be 4. The dataset is preprocessed from a single video recording of vortex shedding. The forward integration time step is set to $dt = \frac{1}{300}$ corresponding to the frame rate of 30 FPS. We set the batch size at 64 and the learning rate to $5e^{-4}$. The thresholding procedure is executed every 300 epochs with thresholds ranging from $(1e^{-4}, 1e^{-3})$. From this extreme low-data limit, we manually perform data augmentation by reusing the latter part of the video once in the training loop to increase the number of available samples.

Baseline experiment on pendulum.

Autoregressive training. The raw pendulum data are collected from a 14-second GoPro recording. The raw data are present difficulties during training because of their high-dimensionality $(\mathbf{1}080 \times 960)$, so we follow the same preprocessing procedure as in (15) to obtain a set of training data with 390 samples, width 24 and height 27. For most of the models, we apply autoregressive training to help the model achieve better long-term prediction capabilities. From the initial input $\{\boldsymbol{X}_1, \boldsymbol{X}_2, ..., \boldsymbol{X}_L\}$ with lag L, the model autoregressively predicts the next frame $\hat{\boldsymbol{X}}_{L+1}$ and use it as a new input $\{\boldsymbol{X}_2, \boldsymbol{X}_3, ..., \hat{\boldsymbol{X}}_{L+1}\}$. This step will be repeated L times to obtain $\{\hat{\boldsymbol{X}}_{L+1}, \hat{\boldsymbol{X}}_{L+2}, ..., \hat{\boldsymbol{X}}_{2L}\}$. We treat this as the prediction and optimize the loss from this quantity. In the following baseline models, we uniformly set $L = 20$.

SINDy. We adapt the dataset and treat each the original pixel as sensors using SINDy (9). The execution of the sparse regression program is slow on large symbolic matrices, so we only include linear libraries and constants. All tested sparse models from SINDy diverge numerically in forward simulation, so we used a model with threshold 0.0 and ℓ_2 regularization 0.05 which effectively fits the dynamical system with ridge regression solution. Given the initial sensor conditions, the algorithm unrolls the values forward with the discovered ODE.

SHRED. To enable prediction capability of SHRED, we train an additional GRU network from available sparse sensor trajectory to serve as the inputs for SHRED (12). The vanilla SHRED network is set up to have the same latent space, decoder size, and optimizer to SINDy-SHRED.

SINDy-AE. We revise the original SINDy autoencoder framework (10) under the same variational form in SINDy-SHRED. For each input data frame, the encoder compresses the high-dimensional image into a single variable $z_{\text{AE}} \in \mathbb{R}$, and the decoder further reconstructs the original data using the latent variable z_{AE}. The SINDy loss is computed via enforcing a SINDy model on the temporal evolution of the latent variable z_{AE}. We set the learning rate, batch size and training epochs to be the same as SINDy-SHRED training.

ResNet. We use the residual neural network (ResNet) (16) as a standard baseline. We set the input sequence length to 20, and we predict the next frames autoregressively. For ResNet, the first convolutional layer has 64 channels with kernel size 3, stride 1 and padding 1. Then, we repeat the residual block three times with two convolutional layers. We use ReLU as the activation function. After the residual blocks, the output is generated via a convolutional layer with kernel size 1, stride 1, and padding 0. We set the batch size to 8, and we use AdamW optimizer with learning rate $1e^{-3}$, weight decay $1e^{-2}$ for the training of 500 epochs.

SimVP. SimVP (17) is the recent state-of-the-art method for video prediction. This method utilizes ConvNormReLU blocks with a spatio-temporal features translator (i.e. CNN). The ConvNormReLU block has two convolutional layers with kernel size 3, stride 1, and padding 1. After 2D batch normalization and ReLU activation, the final forward pass includes a skip connection unit before output. The encoder first performs a 2D convolution with 2D batch normalization and ReLU activation. Then, three ConvNormReLU blocks will complete the input sequence encoding process. The translator in our implementation is a simple CNN which contains two convolutional layers. The decoder has a similar structure to the encoder by reversing its structure. We similarly set the batch size to 8 with AdamW optimizer for 500 epochs.

ConvLSTM. Convolutional Long Short-Term Memory (18) is a classical baseline for the prediction of video sequence and scientific data (e.g. weather, radar echo, and air quality). The ConvLSTM utilizes features after convolution and performs LSTM modeling on hidden states. The ConvLSTM model has two ConvLSTM cells that have an input 2D convolutional layer with kernel size 3 and padding 1 before the LSTM forward pass. The decoder is a simple 2D convolution with kernel size 1, and zero padding. We similarly set the batch size to 8 with AdamW optimizer for 500 epochs.

PredRNN. PredRNN (19) is a recent spatiotemporal modeling technique that builds on the idea of ConvLSTM. We follow the same network architecture setting as in ConvLSTM and similarly set the batch size to 8 with AdamW optimizer for 500 epochs.

SINDy-SHRED. We select and fix 100 pixels as sensor measurements from the entire 648 dimensional space. We remove non-informative sensors, defined as remaining constant through the entire video. We set the lag to 60. For the setting of network architecture in SINDy-SHRED, we follow the same settings as in the prior experiments but with latent dimension of 1. The timestep of forward integration is set to $dt = \frac{1}{300}$ corresponding to frame rate of the video at 30 FPS. We set the batch size at 8 and the learning rate to $5e^{-4}$. The thresholding procedure is executed every 300 epochs with thresholds ranging from $(0.4, 4.0)$. We include 3 stacked GRU layers, and a two-layer ReLU decoder with 16 and 64 neurons. We use dropout to avoid overfitting with a dropout rate of 0.1. SINDy-SHRED discovers two candidate models.

Sea-surface temperature data. The SST dataset is an observational reanalysis produce provided by NOAA (20). Every snapshot includes a 180×360 grid and the data are preprocessed through interpolation combining in-situ buoy, ship, and satellite observations. The SST dataset from NOAA is directly adapted into the training pipeline of SINDy-SHRED. We set the latent dimension to 3 because we observe only minor impacts on the reconstruction accuracy when the latent dimension is ≥ 4. We include 2 stacked GRU layers and consider the , and a two-layer ReLU decoder with 350 and 400 neurons. For the E-SINDy regularization, we set the polynomial order to be 3 and the ensemble number is 10. In the latent state forward simulation, we use Euler integration with $dt = \frac{1}{520}$, which will generate the prediction of next week via 10 forward integration steps. During training, we apply the AdamW optimizer with a learning rate of $1e^{-3}$ and a weight decay of $1e^{-2}$. The batch size is 128 with 1,000 training epochs. The thresholds for E-SINDy range uniformly from 0.1 to 1.0, and the thresholding procedure will be executed every 100 epochs. We use dropout to avoid overfitting with a dropout rate of 0.1. The training time is within 30 minutes from a single NVIDIA GeForce RTX 2080 Ti.

3D atmospheric ozone concentration. The ozone dataset is synthetic and derived from the GEOS-Chem chemical transport model, which numerically integrates coupled advection-diffusion-reaction equation (21). In SINDy-SHRED training, we set the lag parameter is set to 100. Thus, for each input-output pair, the input consists of the 62.5 day measurements of the selected sensors, while the output is the measurement across the entire 3D domain. In SINDy-SHRED, we follow the same network architecture as in the SST experiment. We set $dt = 0.025$, and the thresholds for E-SINDy range uniformly from 0.015 to 0.15. The thresholding procedure will be executed every 300 epochs, and we apply AdamW optimizer with learning rate $1e^{-3}$.

Isotropic turbulent flow. The isotropic turbulent flow dataset is synthetic and obtained from the Johns Hopkins Turbulence Database (JHTDB) (22). It is generated from the incompressible Navier-Stokes equations using pseudo-spectral method with periodic boundary condition. The internal DNS step is 0.0002. The generation is based upon a 1024^3 grid, and we obtain a 350×350 spatial slice of the original pressure field. In the isotropic flow experiment, due to its complex nature, we select the latent dimension to be 16 with lag 100. The forward integration time step is set to $dt = 0.0002$. We set the batch size to 128 and the learning rate to $5e^{-4}$. The thresholding procedure is executed every 100 epochs with thresholds ranging from $(0.15, 1.5)$. The total training epoch is 300.

Experiment on the 2D Kolmogorov flow

The 2D Kolmogorov flow data is a chaotic turbulent flow generated from the pseudospectral Kolmogorov flow solver (23). The solver numerically solves the divergence-free Navier-Stokes equation:

$$\begin{cases} \nabla \cdot \boldsymbol{u} = 0 \\ \partial_t \boldsymbol{u} + \boldsymbol{u} \cdot \nabla \boldsymbol{u} = -\nabla \boldsymbol{p} + \nu \Delta \boldsymbol{u} + f, \end{cases} \quad [64]$$

where $\boldsymbol{u}$ stands for the velocity field, $\boldsymbol{p}$ stands for the pressure, ν is the kinematic viscosity and f describes an external forcing term. In this case, we consider doubly periodic domain $\Omega = [0, 2\pi)^2$ with periodic boundary conditions in both spatial directions. We specify the external force to be $f(x, y) = \sin(n_f y)\mathbf{e}_1$ where $n_f = 4$ and $\mathbf{e}_1 = [1, 0]^\top$. Setting the Reynolds number to 30, which is within the weakly chaotic regime (24), and the spatial field has resolution 80×80. We simulate the system forward for

180 seconds with $6,000$ available frames. We standardize the data within the range of $(0,1)$ and randomly fix 10 sensors from the 6,400 available spatial locations (0.16%). The lag parameter is set to 360.

For the setting of SINDy-SHRED, we slightly change the neural network setting because the output domain is 2D. Therefore, after the GRU unit, we use two shallow decoders to predict the output of the 2D field. The two decoders are two-layer ReLU networks with 350 and 400 neurons. We set the latent dimension to 3. The time step for forward integration is set to $dt = 0.003$ which corresponds to the FPS during data generation. We set the batch size to 256 and the learning rate to $5e^{-4}$ using the Lion optimizer (25). The thresholding procedure is executed every 100 epoch with the total number of training epochs as 200. The thresholds range from $(0.4, 4)$.

As a chaotic system, the latent space of the Kolmogorov flow is much more complex than all the prior examples we considered. Thus, we further apply seasonal-trend decomposition from the original latent space. We define the representation of the latent state space after decomposition as $(z_1, z_2, z_3, z_4, z_5, z_6)$, where (z_{2i}, z_{2i+1}) is the seasonal trend pair of the original latent space.

$$\begin{cases} \dot{z}_1 &= -0.007z_3 + 0.009z_5, \\ \dot{z}_2 &= -0.207z_4, \\ \dot{z}_3 &= -0.011z_1 - 0.008z_5, \\ \dot{z}_4 &= 0.103z_2, \\ \dot{z}_5 &= -0.012z_1 + 0.006z_3. \\ \dot{z}_6 &= 0.151z_1z_2. \end{cases} \quad [65]$$

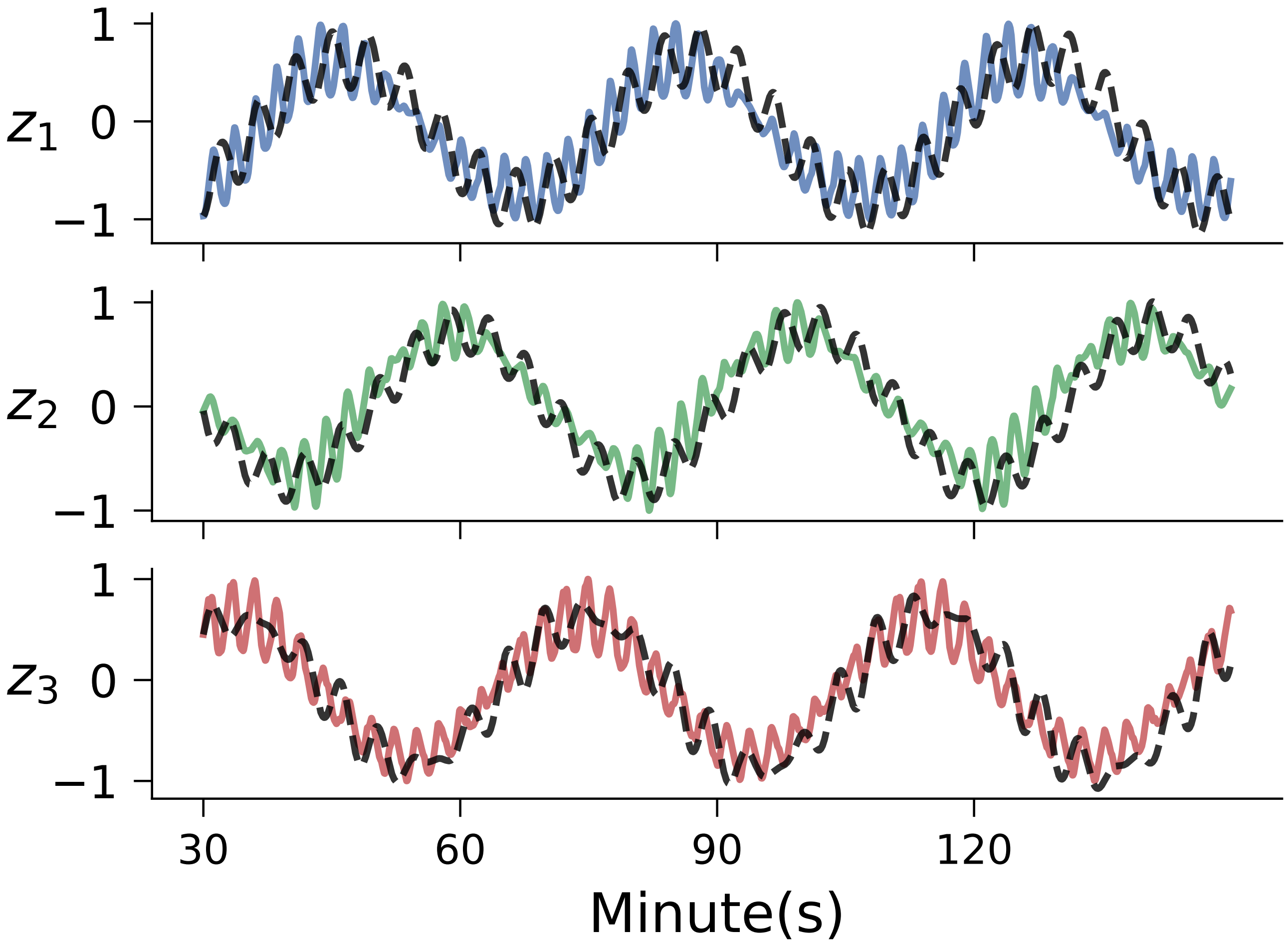

Figure S14. Extrapolation of latent representation in SINDy-SHRED from the discovered dynamical system for the 2D Kolmogorov flow data. Colored: true latent representation. Black: SINDy extrapolation.

In Eqn. 65, we find that z_1, z_3, z_5 are essentially a linear system. z_2, z_4, z_6 capture higher-order effects that are difficult to model without signal separation. We generate the trajectory from the initial condition at time point 0 and perform forward

integration in Fig. S14. As we increase the Reynolds number, the discovery fails to produce robust predictions.

This representation also demonstrates nice predictions for future frames. In Fig. S15, the future prediction has an averaged MSE error of 0.035 for all available data samples. The sensor-level prediction in Fig. S28 further demonstrates the details of the reconstruction.

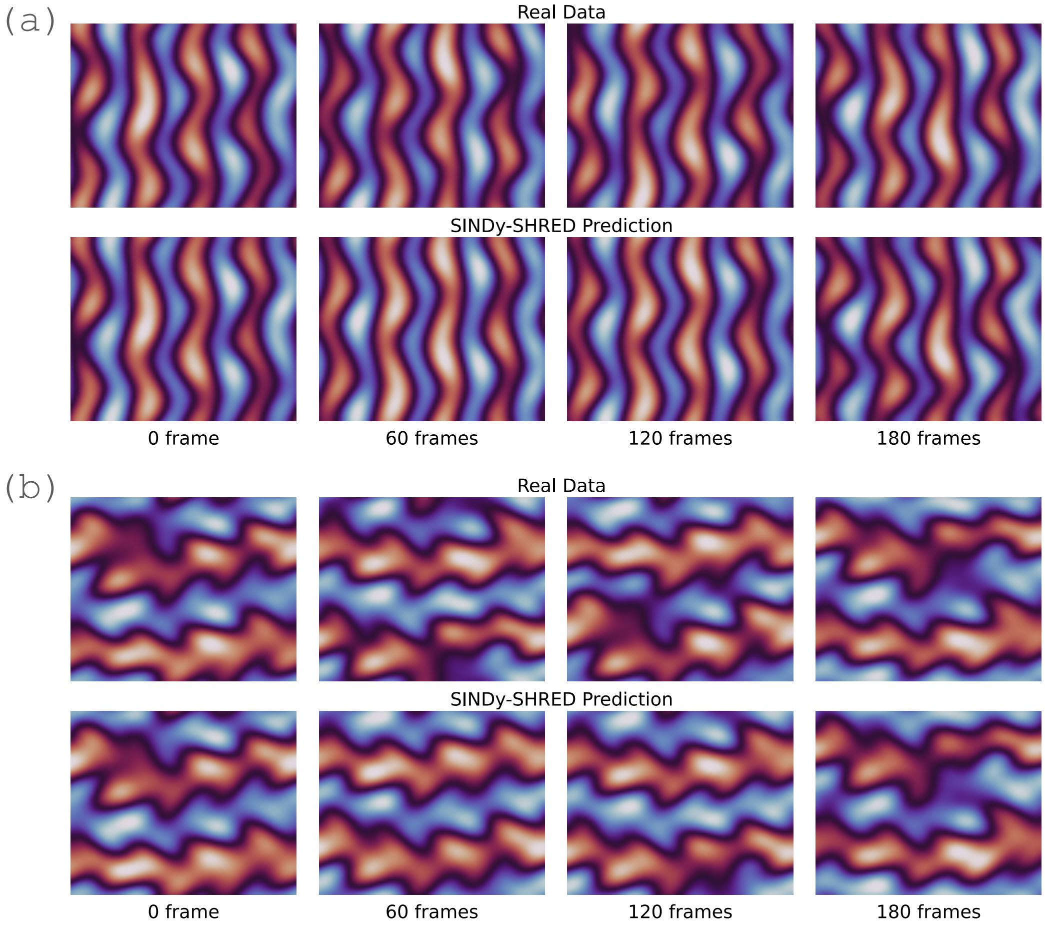

Figure S15. Long-term predictiion via SINDy-SHRED for 2D Kolmogorov flow data.

Sensor level plots of experiments

Sea surface temperature. 3D visualization of SINDy-SHRED

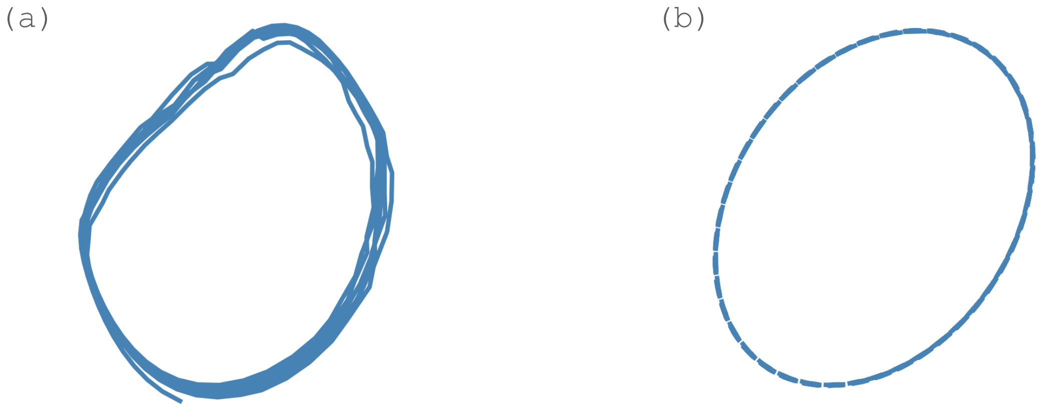

Figure S16. 3D reconstruction of the original latent space and SINDy simulated latent space.

Long-term extrapolation of SINDy-SHRED.

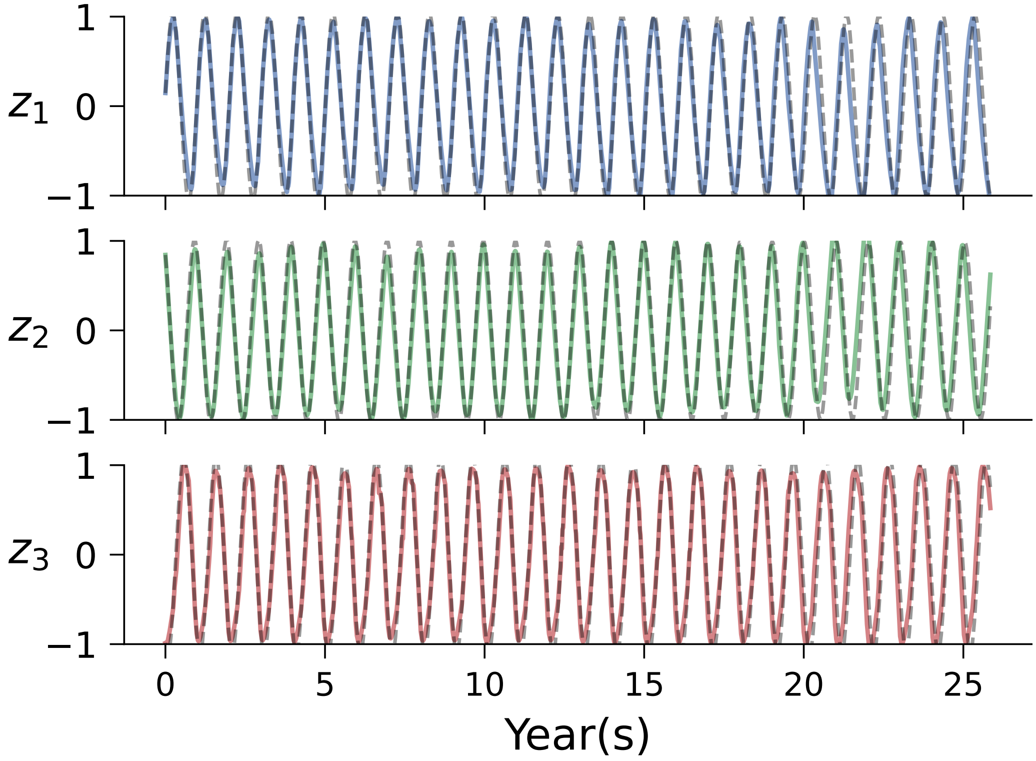

Figure S17. Extrapolation of latent representation in SINDy-SHRED from the discovered dynamical system for SST over the entire 27 years. Colored: true latent representation. Grey: SINDy extrapolation.

Sensor-level prediction on the SST dataset.

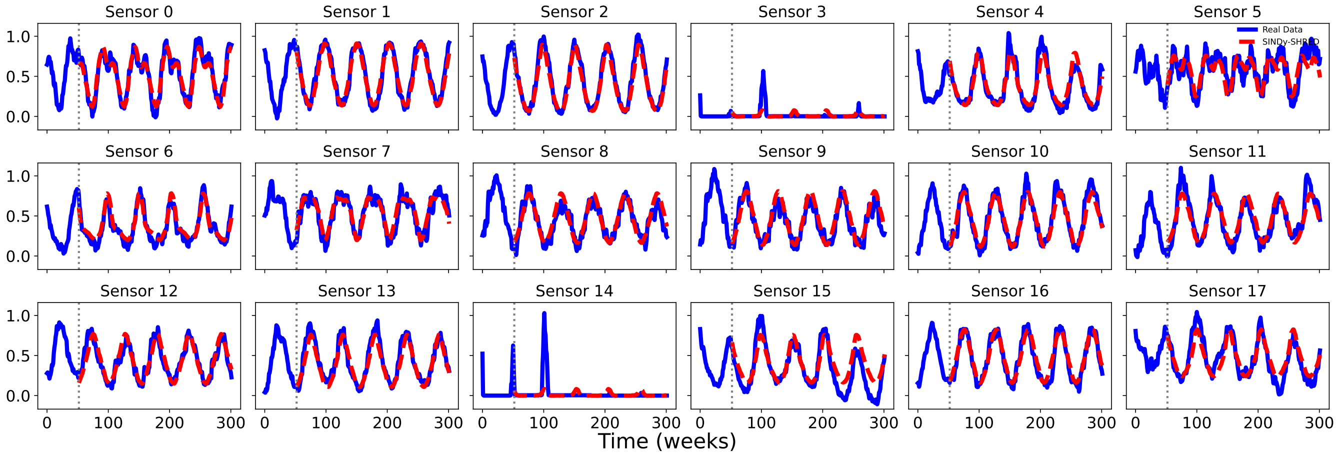

Figure S18. Extrapolation of SINDy-SHRED for sensor-level predictions on the SST data. We randomly picked 18 sensors from spatial locations that are not in the sparse sensor training. The extrapolation shows the SINDy-SHRED prediction for the following 300 weeks.

Ozone data. **Convergence behavior of SINDy-SHRED on the Ozone dataset.**

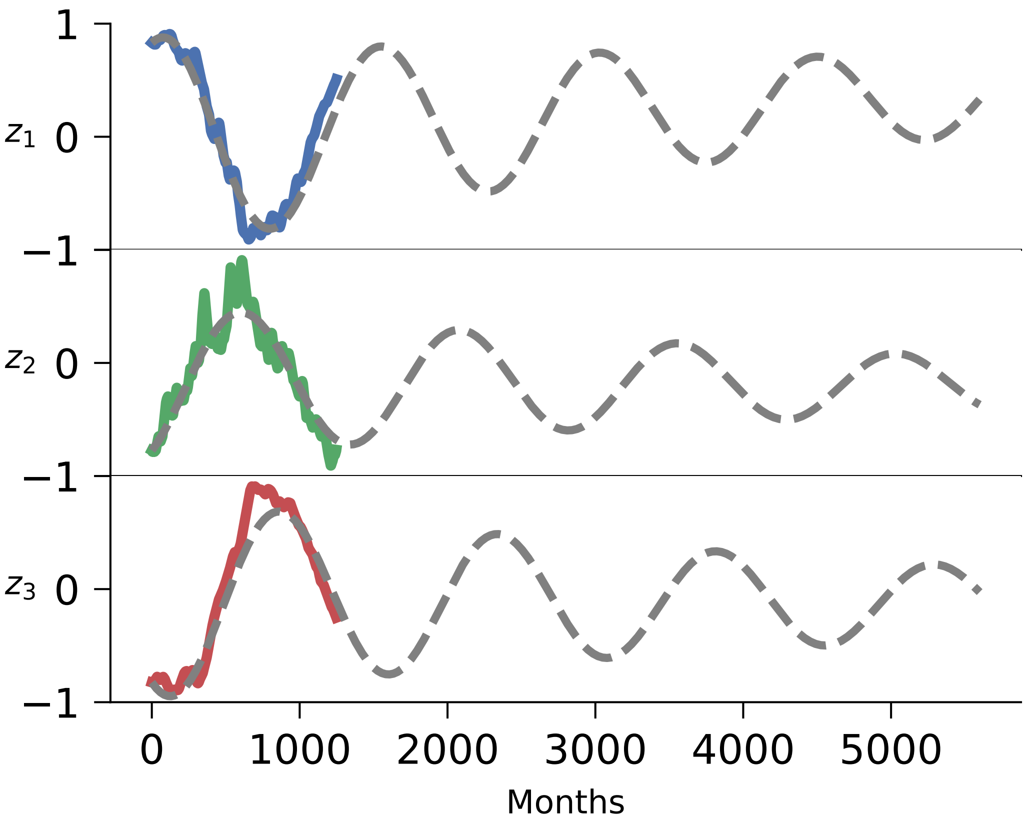

Figure S19. Long term extrapolation of Ozone data. The latent SINDy model presents a convergence behavior towards the mean-field solution.

482 **Sensor-level prediction on the Ozone dataset.**

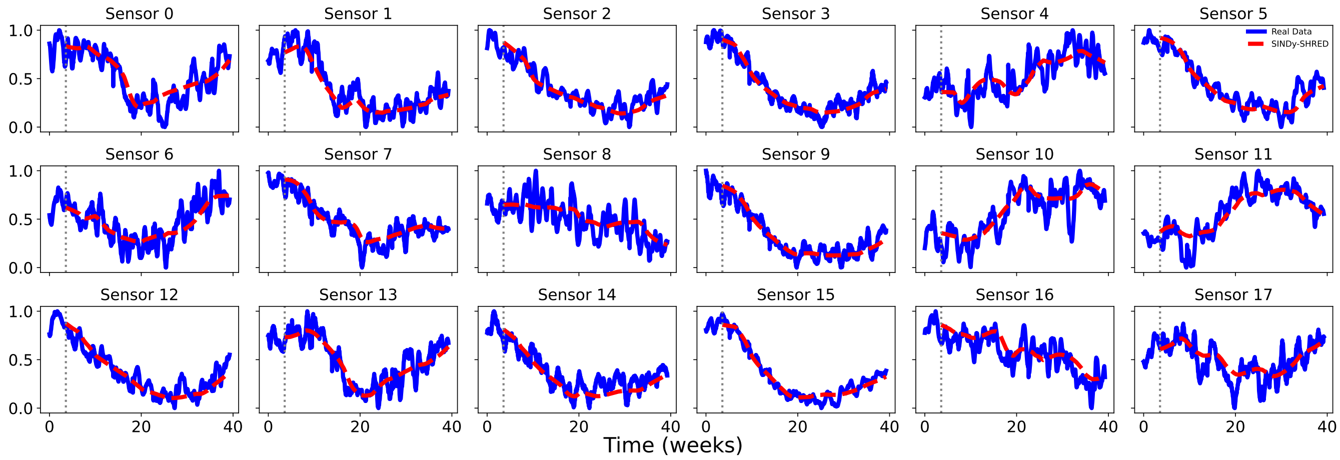

Figure S20. Extrapolation of SINDy-SHRED for sensor-level predictions on the Ozone data. We randomly picked 18 sensors from spatial locations that are not in the sparse sensor training. The extrapolation shows the SINDy-SHRED prediction for the following 40 weeks.

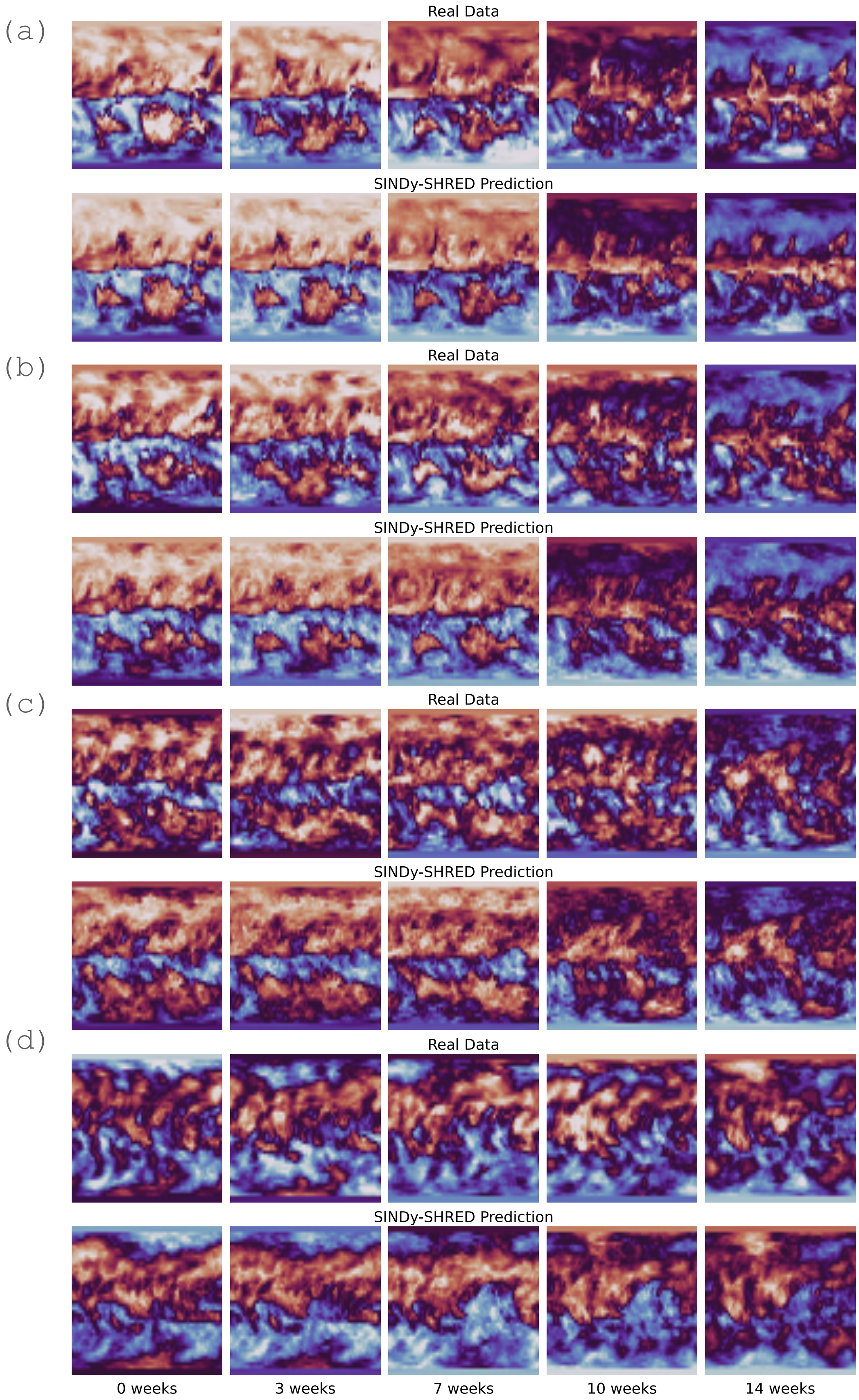

Figure S21. Reconstruction of atmospheric ozone concentration data for different elevation (a) 0 km (b) 4 km (c) 8 km (d) 12 km.

Flow over a cylinder. Latent space of Koopman-SHRED

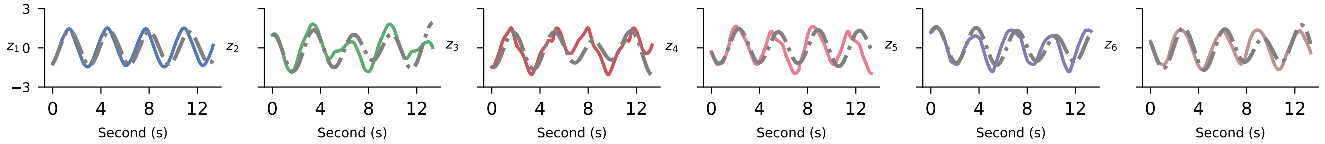

Figure S22. Extrapolation of latent representation in Koopman-SHRED from the discovered dynamical system for flow over a cylinder data. Colored: true latent representation. Grey: SINDy extrapolation.

Sensor-level prediction on the flow over a cylinder dataset.

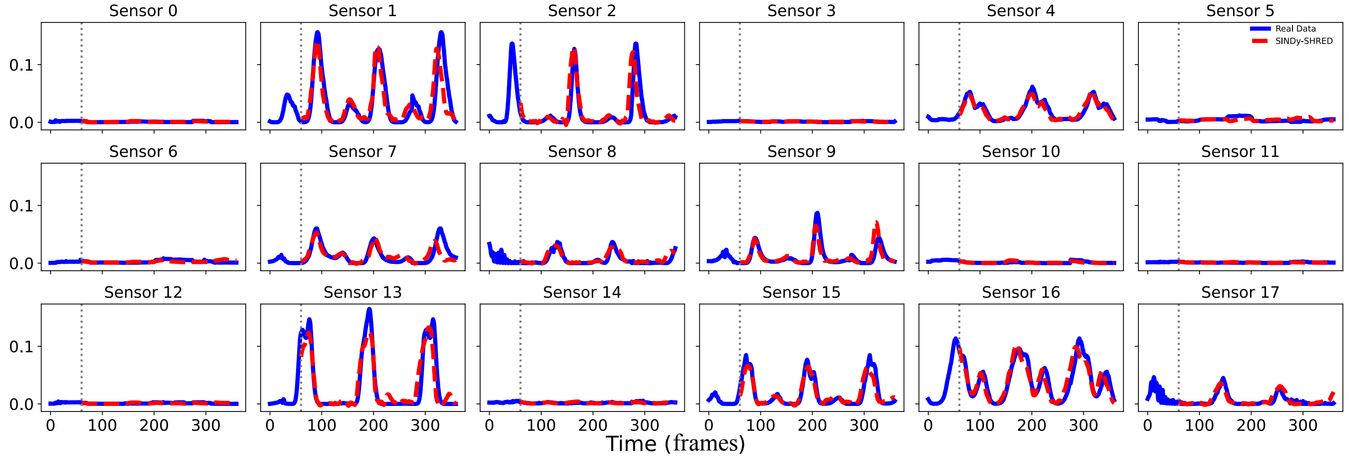

Figure S23. Extrapolation of SINDy-SHRED for sensor-level predictions on the flow over a cylinder data. We randomly picked 18 sensors from spatial locations that are not in the sparse sensor training. The extrapolation shows the SINDy-SHRED prediction for the following 400 frames.

Long-term extrapolation on the flow over a cylinder dataset.

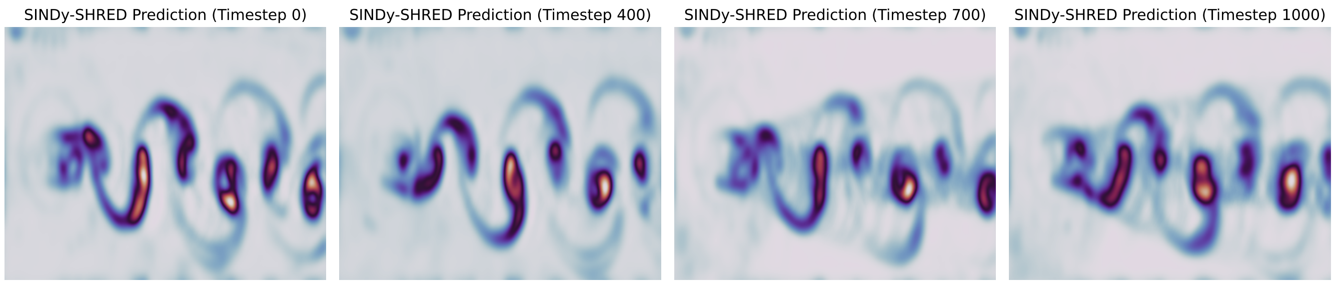

Figure S24. Prediction of the flow over a cylinder data from time step 0 (reconstruction) to 1000 frames. We note this extrapolation is completely out of the dataset. The real data for testing is only available up to 500 frames.

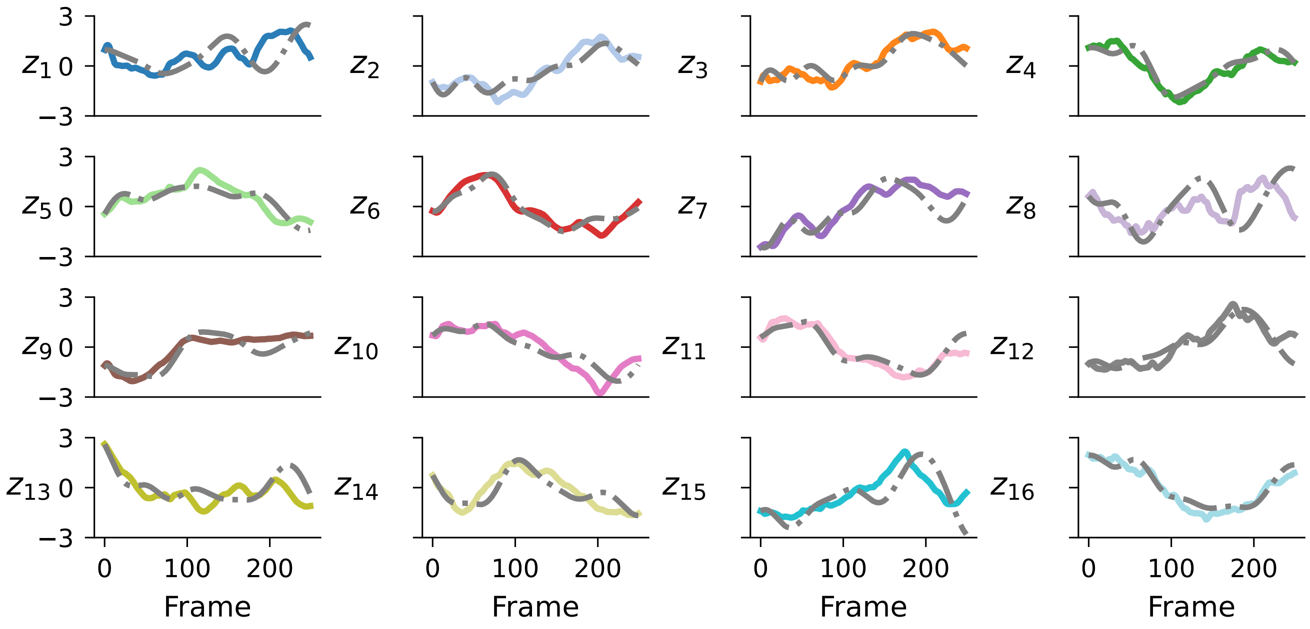

Figure S25. Extrapolation of all 16 latent representation in SINDy-SHRED from the discovered dynamical system for isotropic turbulent flow data for 250 frames. Colored: true latent representation. Grey: SINDy extrapolation.

486 **Isotropic turbulent flow.** Sensor-level prediction on the isotropic turbulent flow dataset.

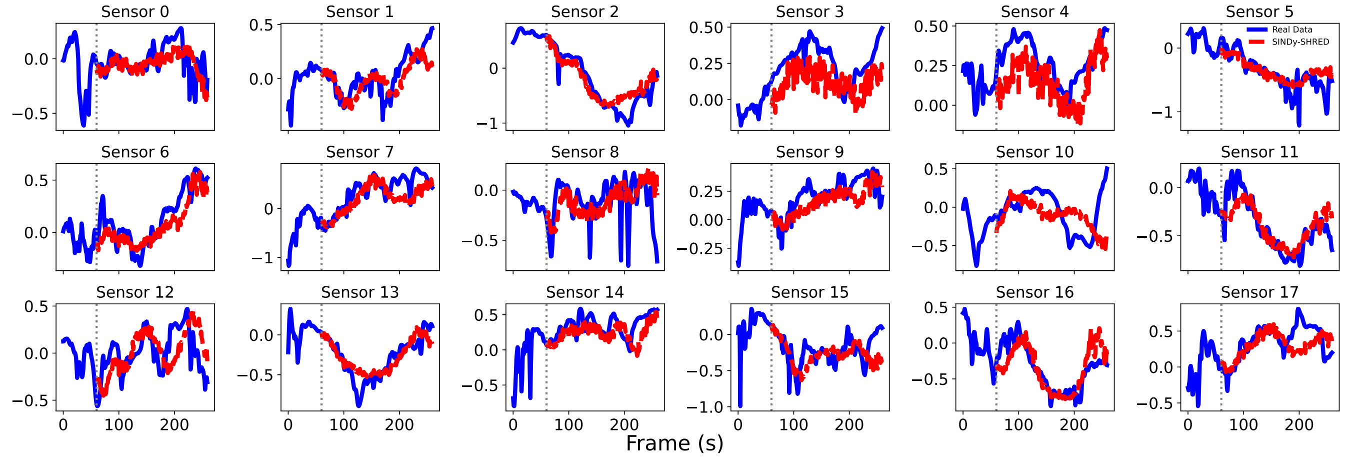

Figure S26. Extrapolation of SINDy-SHRED for sensor-level predictions on the isotropic turbulent flow data. We randomly picked 18 sensors from spatial locations that are not in the sparse sensor training. The extrapolation shows the SINDy-SHRED prediction for the following 250 frames.

Pendulum. Sensor-level prediction on the moving pendulum dataset.

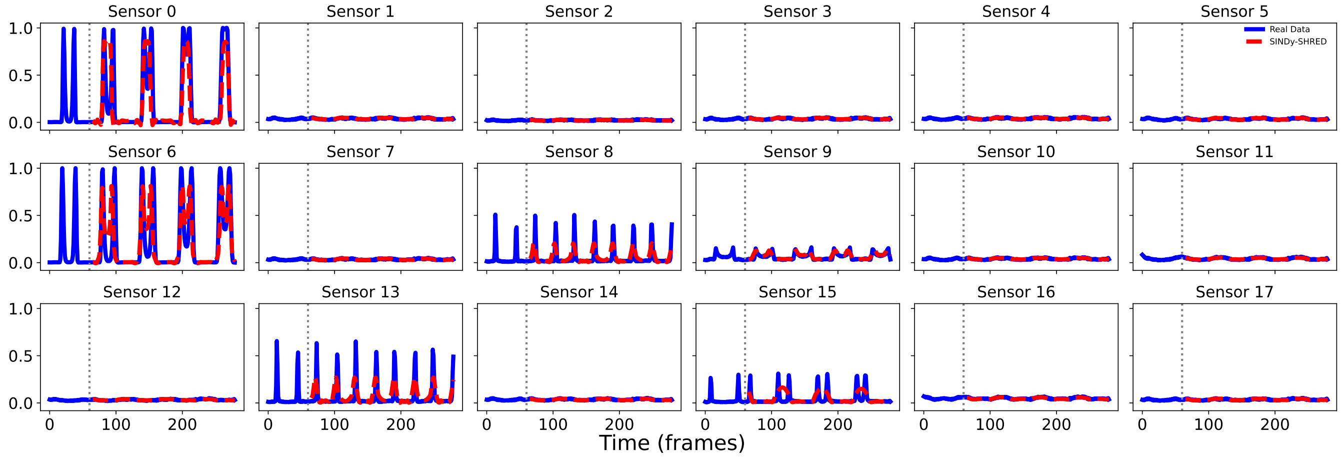

Figure S27. Extrapolation of SINDy-SHRED for sensor-level predictions on the moving pendulum data. We randomly picked 18 sensors from spatial locations that are not in the sparse sensor training. The extrapolation shows the SINDy-SHRED prediction for the following 382 frames.

Kolmogorov flow. Sensor-level prediction on the chaotic 2D Kolmogorov flow dataset.

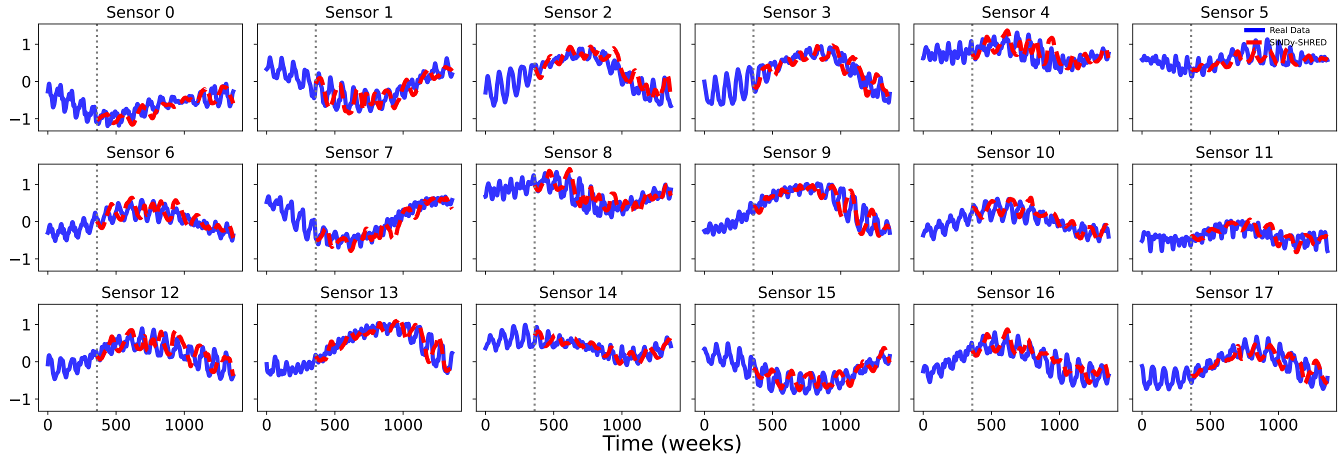

Figure S28. Extrapolation of SINDy-SHRED for sensor-level predictions on the 2D Kolmogorov flow data. We randomly picked 18 sensors from spatial locations that are not in the sparse sensor training. The extrapolation shows the SINDy-SHRED prediction for the following 1500 frames.

Analysis of learned ODEs

Sea-surface temperature. The analytic solution of the dynamical system discovered from the SST data

$$\mathbf{z}(t) = c_1\mathbf{v}_1 e^{(-0.01+6.24i)t} + c_2\mathbf{v_2}e^{(-0.01-6.24i)t} + c_3\mathbf{v}_3 e^{0.02t}, \qquad [66]$$

where $v_1 = \begin{pmatrix} -0.39+0.38i \\ -0.19-0.52i \\ 0.63 \end{pmatrix}$, $v_2 = \begin{pmatrix} -0.39-0.38i \\ -0.19+0.52i \\ 0.63 \end{pmatrix}$, $v_3 = \begin{pmatrix} 0.68 \\ 0.33 \\ 0.65 \end{pmatrix}$, and c_1, c_2, and c_3 depend on the initial condition.

Ozone data. The complete closed form solution to the discovered ODE from Ozone data is given by:

$$\mathbf{z}(t) = T\text{diag}\begin{pmatrix} e^{(-0.003+0.0079i)t} \\ e^{(-0.003+0.0079i)t} \\ -0.003 \end{pmatrix} T^{-1}\mathbf{z}_0 \qquad +T\left(\text{diag}\begin{pmatrix} (-42-111i)e^{(-0.003+0.0079i)t} \\ (-42+111i)e^{(-0.003-0.0079i)t} \\ -333e^{-0.003t} \end{pmatrix}\Bigg|_0^t T^{-1}\begin{pmatrix} -0.002 \\ 0 \\ 0.002 \end{pmatrix}\right), \qquad [67]$$

where $\mathbf{z}_0$ is the state at $t = 0$ and

$$T = \begin{pmatrix} 0.66 & 0.66 & -0.99 \\ -0.30-0.35i & -0.30+0.35i & 0.061 \\ -0.59+0.11i & -0.59-0.11i & 0.16 \end{pmatrix}.$$

Algorithm 1 Latent state space regularization via SINDy

Input: sensor $\boldsymbol{S}_{L+1:T}$, $\boldsymbol{X}_{L+1:T}$, SINDy library $\Theta(\cdot)$, timestep Δt, regularization λ, threshold fequency δ_{thres}, Euler mini-step number k, temporal lag parameter L, learning rate η, total epoch T_{epoch}.

function LatentSpaceSINDy($\boldsymbol{S}_{L+1:T}, \boldsymbol{X}_{L+1:T}, \Delta t, \lambda, \delta_{\text{thres}}, k, \eta, L$) :
for i in $0, 1, \cdots, T_{\text{epoch}}$: **do**
  $\boldsymbol{Z}_T,\ \boldsymbol{Z}_{T+1} = f_{\theta_{\text{GRU}}}(\boldsymbol{S}_{L+1:T-1}),\ f_{\theta_{\text{GRU}}}(\boldsymbol{S}_{L+2:T})$;
  for j in $(0, k-1)$: **do** ▷ SINDy forward simulation
    $\boldsymbol{Z}^{\text{SINDy}}_{T+\frac{j+1}{k}\Delta t} = \boldsymbol{Z}^{\text{SINDy}}_{T+\frac{j}{k}\Delta t} + \Theta(\boldsymbol{Z}^{\text{SINDy}}_{T+\frac{j}{k}\Delta t})\Xi\Delta t$
  end for
  $\hat{\boldsymbol{X}}_{L+2:T} = f_{\theta_D}(\boldsymbol{Z}_{T+1})$ ▷ SHRED reconstruction
  $(\theta_{\text{GRU}}, \Xi, \theta_D) = (\theta_{\text{GRU}}, \Xi, \theta_D) - \eta\nabla\mathcal{L}$ ▷ with $\mathcal{L}$ defined in equation 4
  if $i \mod \delta_{\text{thres}} = 0$ **then**
    $\Xi[|\Xi| < \text{threshold}] = 0$
  end if
end for ▷ Train until converges

Output: GRU network weights θ_{GRU}, SINDy coefficients Ξ, decoder network weights θ_D.

Flow over a cylinder. The complete closed form solution to the discovered dynamical system from the flow over a cylinder data is given by:

$$\mathbf{z}(t) = T\text{diag}\begin{pmatrix} e^{(-0.01+1.52i)t} \\ e^{(-0.01-1.52i)t} \\ e^{(0.11+1.05i)t} \\ e^{(0.11-1.05i)t} \\ e^{-0.20t} \\ 0 \end{pmatrix} T^{-1}\mathbf{z}_0, \quad [68]$$

where $\mathbf{z}_0$ is the state at $t = 0$ and

$$T = \begin{pmatrix} -0.35+0.20i & -0.35-0.20i & 0.028-0.006i & 0.028+0.006i & 0.005 & 0 \\ 0.15-0.39i & 0.15+0.39i & -0.12-0.45i & -0.12+0.45i & 0.68 & 0 \\ -0.10+0.37i & -0.10-0.37i & 0.034+0.57i & 0.034+0.57i & 0.23 & 1 \\ 0.24+0.33i & 0.24-0.33i & 0.013-0.21i & 0.013+0.21i & -0.37 & 0 \\ -0.13-0.38i & -0.13+0.38i & 0.034-0.27i & 0.034+0.27i & -0.55 & 0 \\ 0.44 & 0.44 & 0.58 & 0.58 & 0.21 & 0 \end{pmatrix}.$$

Isotropic turbulence flow. The complete closed-form solution of the discovered latent dynamical system for the isotropic turbulent flow data is the following:

$$\mathbf{z}(t) = T\text{diag}\begin{pmatrix} e^{(0.47+9.39i)t} \\ e^{(0.47+9.39i)t} \\ e^{(0.05+11.90i)t} \\ e^{(0.05+11.90i)t} \\ e^{(0.03+13.42i)t} \\ e^{(0.03+13.42i)t} \\ e^{(-0.04+3.46i)t} \\ e^{(-0.04+3.46i)t} \\ e^{-0.26t} \\ e^{(-0.27+5.44i)t} \\ e^{(-0.27+5.44i)t} \\ e^{(-0.75+8.30i)t} \\ e^{(-0.75+8.30i)t} \\ e^{(-1.37+18.72i)t} \\ e^{(-1.37+18.72i)t} \\ e^{-3.39t} \end{pmatrix} T^{-1}\mathbf{z}_0, \quad [69]$$

where $\mathbf{z}_0$ is the state at $t = 0$ and T contains all eigenvectors.

Algorithm

References

1. Xinwei Shen and Nicolai Meinshausen. Engression: Extrapolation for nonlinear regression? *arXiv preprint arXiv:2307.00835*, 2023.

2. Peter L Bartlett, Andrea Montanari, and Alexander Rakhlin. Deep learning: a statistical viewpoint. *Acta numerica*, 30: 87–201, 2021.
3. Noah Golowich, Alexander Rakhlin, and Ohad Shamir. Size-independent sample complexity of neural networks. In *Conference On Learning Theory*, pages 297–299. PMLR, 2018.
4. Peter L Bartlett and Shahar Mendelson. Rademacher and gaussian complexities: Risk bounds and structural results. *Journal of Machine Learning Research*, 3(Nov):463–482, 2002.
5. Michel Ledoux and Michel Talagrand. *Probability in Banach Spaces: isoperimetry and processes.* Springer Science & Business Media, 2013.
6. Aladin Virmaux and Kevin Scaman. Lipschitz regularity of deep neural networks: analysis and efficient estimation. *Advances in Neural Information Processing Systems*, 31, 2018.
7. Hao Li, Zheng Xu, Gavin Taylor, Christoph Studer, and Tom Goldstein. Visualizing the loss landscape of neural nets. *Advances in neural information processing systems*, 31, 2018.
8. Jeffrey L Elman. Finding structure in time. *Cognitive science*, 14(2):179–211, 1990.
9. Steven L Brunton, Joshua L Proctor, and J Nathan Kutz. Discovering governing equations from data by sparse identification of nonlinear dynamical systems. *Proceedings of the national academy of sciences*, 113(15):3932–3937, 2016.
10. Kathleen Champion, Bethany Lusch, J Nathan Kutz, and Steven L Brunton. Data-driven discovery of coordinates and governing equations. *Proceedings of the National Academy of Sciences*, 116(45):22445–22451, 2019.
11. Kai Fukami, Takaaki Murata, Kai Zhang, and Koji Fukagata. Sparse identification of nonlinear dynamics with low-dimensionalized flow representations. *Journal of Fluid Mechanics*, 926:A10, 2021.
12. Jan P Williams, Olivia Zahn, and J Nathan Kutz. Sensing with shallow recurrent decoder networks. *Proceedings of the Royal Society A*, 480(2298):20240054, 2024.
13. Samuel E Otto, Nicholas Zolman, J Nathan Kutz, and Steven L Brunton. A unified framework to enforce, discover, and promote symmetry in machine learning. *arXiv preprint arXiv:2311.00212*, 2023.
14. Stephen B Pope. Turbulent flows. *Measurement Science and Technology*, 12(11):2020–2021, 2001.
15. L Mars Gao and J Nathan Kutz. Bayesian autoencoders for data-driven discovery of coordinates, governing equations and fundamental constants. *Proceedings of the Royal Society A*, 480(2286):20230506, 2024.
16. Kaiming He, Xiangyu Zhang, Shaoqing Ren, and Jian Sun. Deep residual learning for image recognition. In *Proceedings of the IEEE conference on computer vision and pattern recognition*, pages 770–778, 2016.
17. Zhangyang Gao, Cheng Tan, Lirong Wu, and Stan Z Li. Simvp: Simpler yet better video prediction. In *Proceedings of the IEEE/CVF conference on computer vision and pattern recognition*, pages 3170–3180, 2022.
18. Xingjian Shi, Zhourong Chen, Hao Wang, Dit-Yan Yeung, Wai-Kin Wong, and Wang-chun Woo. Convolutional lstm network: A machine learning approach for precipitation nowcasting. *Advances in neural information processing systems*, 28, 2015.
19. Yunbo Wang, Mingsheng Long, Jianmin Wang, Zhifeng Gao, and Philip S Yu. Predrnn: Recurrent neural networks for predictive learning using spatiotemporal lstms. *Advances in neural information processing systems*, 30, 2017.
20. Richard W Reynolds, Nick A Rayner, Thomas M Smith, Diane C Stokes, and Wanqiu Wang. An improved in situ and satellite sst analysis for climate. *Journal of climate*, 15(13):1609–1625, 2002.
21. Isabelle Bey, Daniel J Jacob, Robert M Yantosca, Jennifer A Logan, Brendan D Field, Arlene M Fiore, Qinbin Li, Honguy Y Liu, Loretta J Mickley, and Martin G Schultz. Global modeling of tropospheric chemistry with assimilated meteorology: Model description and evaluation. *Journal of Geophysical Research: Atmospheres*, 106(D19):23073–23095, 2001.
22. Yi Li, Eric Perlman, Minping Wan, Yunke Yang, Charles Meneveau, Randal Burns, Shiyi Chen, Alexander Szalay, and Gregory Eyink. A public turbulence database cluster and applications to study lagrangian evolution of velocity increments in turbulence. *Journal of Turbulence*, (9):N31, 2008.
23. Claudio Canuto, M Yousuff Hussaini, Alfio Quarteroni, and Thomas A Zang. *Spectral methods: evolution to complex geometries and applications to fluid dynamics.* Springer Science & Business Media, 2007.
24. Gary J Chandler and Rich R Kerswell. Invariant recurrent solutions embedded in a turbulent two-dimensional kolmogorov flow. *Journal of Fluid Mechanics*, 722:554–595, 2013.
25. Xiangning Chen, Chen Liang, Da Huang, Esteban Real, Kaiyuan Wang, Hieu Pham, Xuanyi Dong, Thang Luong, Cho-Jui Hsieh, Yifeng Lu, et al. Symbolic discovery of optimization algorithms. *Advances in neural information processing systems*, 36, 2024.
